# New glycoside hydrolase families of β‐1,2‐glucanases

**DOI:** 10.1002/pro.70147

**Published:** 2025-05-24

**Authors:** Masahiro Nakajima, Nobukiyo Tanaka, Sei Motouchi, Kaito Kobayashi, Hisaka Shimizu, Koichi Abe, Naoya Hosoyamada, Naoya Abara, Naoko Morimoto, Narumi Hiramoto, Ryosuke Nakata, Akira Takashima, Marie Hosoki, Soichiro Suzuki, Kako Shikano, Takahiro Fujimaru, Shiho Imagawa, Yukiya Kawadai, Ziyu Wang, Yoshinao Kitano, Takanori Nihira, Hiroyuki Nakai, Hayao Taguchi

**Affiliations:** ^1^ Department of Applied Biological Science, Faculty of Science and Technology Tokyo University of Science Noda Chiba Japan; ^2^ Artificial Intelligence Research Center National Institute of Advanced Industrial Science and Technology (AIST) Tokyo Japan; ^3^ Department of Biotechnology The University of Tokyo Tokyo Japan; ^4^ Graduate School of Agriculture Kyoto University Kyoto Japan; ^5^ Faculty of Agriculture Niigata University Niigata Niigata Japan; ^6^ Faculty of Engineering Niigata Institute of Technology Kashiwazaki Niigata Japan

**Keywords:** carbohydrate‐active enzyme, glycoside hydrolase family, β‐1,2‐glucan, β‐1,2‐glucooligosaccharide, β‐1,2‐glucanase

## Abstract

β‐1,2‐Glucans are natural glucose polymers produced by bacteria and play important physiological roles, including as symbiotic or pathogenic factors and in osmoregulation. Glycoside hydrolase (GH) families related to β‐1,2‐glucan metabolism (GH144, GH162, and GH189) have recently been created by identification of two β‐1,2‐glucanases and a β‐1,2‐glucanotransferase, respectively. In this study, we further found four phylogenetically new groups with unknown functions (Groups 1–4) by sequence database analysis using enzymes from GH144 and GH162 as queries. Biochemical analysis of representative proteins in these groups revealed that the proteins in Groups 1–3 showed hydrolytic activity specific to β‐1,2‐glucan, while no substrate was found for the Group 4 protein. The kinetic parameters of the enzymes of Groups 1–3 were similar to GH144 and GH162 β‐1,2‐glucanases, indicating that these enzymes were β‐1,2‐glucanases. Optical rotation analysis revealed that the β‐1,2‐glucanases followed an anomer‐inverting mechanism. Structural analysis of the proteins in Groups 1–4 revealed that they possess (α/α)_6_‐barrel folds similar to those of GH144, GH162, and GH189 enzymes. Comparison of spatial positions of predicted acidic catalytic residues suggested that Groups 1–3 and GH144 had the same reaction mechanism. Overall, phylogenetic, biochemical, and structural analyses revealed that Groups 1–3 are new GH families, GH192, GH193, and GH194, respectively, and that the three families belong to clan GH‐S (clan GH, classification based on structural similarity) as GH144 and GH162.

## 1 INTRODUCTION

Carbohydrates are used not only as energy sources, but also for the storage of polysaccharides and in cell skeletons, including in the cell walls of plants and fungi (Popper et al., 2011; Ruiz‐Herrera & Ortiz‐Castellanos, 2019; Zeeman et al., 2010). Indigestible carbohydrates known as prebiotics influence the microbiome in the large intestine by enhancing the growth of beneficial bacteria, which is important for immuno‐stimulation (Lenardon et al., 2010; Nishimoto & Kitaoka, 2007; Noss et al., 2013). The wide variety of the physiological functions of carbohydrates in organisms derives from the complexity of the carbohydrate structures. It is estimated that over 1 billion different structures are theoretically possible for a hexasaccharide (Laine, 1994). In response to such complexity, the enzymes associated with carbohydrate synthesis and degradation have evolved to have diverse amino acid sequences and tertiary structures.

Such groups of enzymes are classified into families in the Carbohydrate‐Active enZYmes (CAZy) database (http://www.cazy.org/Home.html) primarily based on the amino acid sequences (Coutinho et al., 2003; Drula et al., 2022; Henrissat et al., 2008; Levasseur et al., 2013) and are called “CAZymes.” These families are categorized into glycoside hydrolase (GH) families, glycosyltransferase (GT) families, carbohydrate esterase (CE) families, polysaccharide lyase (PL) families, and auxiliary activity (AA) families (AA families contain redox‐active enzymes) (Levasseur et al., 2013) mainly based on the types of reactions that the enzymes catalyze. Enzymes in the GH families are the most abundant in the CAZy database, and the number of identified GH families continues to increase and exceeded 180 as of January 2025, suggesting the importance of the GH family enzymes. However, considering the complexity of carbohydrate structures, it is presumed that many “CAZymes” have not been identified yet.

β‐1,2‐Glucans are polysaccharides composed of glucose (Glc), which are found in nature mainly in cyclic forms. Although β‐1,2‐glucans are thought to be rare compared with the other glucans, such as cellulose (β‐1,4‐linkage) and laminarin (β‐1,3‐linkage), β‐1,2‐glucans are involved in the bacterial infection of animal and plant cells and in hypo‐osmotic adaptation (Arellano‐Reynoso et al., 2005; Briones et al., 2001; Javvadi et al., 2018; Rigano et al., 2007). Recently, it has been reported that β‐1,2‐glucotriose, decomposed products of β‐1,2‐glucans, showed a pattern‐triggered immune response in plants by producing reactive oxygen species (Fuertes‐Rabanal et al., 2024). A β‐1,2‐glucan was first found as an extracellular polysaccharide from *Rhizobium radiobacter* (formerly *Agrobacterium tuberculosis*), a plant pathogen forming crown galls on plant roots in 1940 (McIntire et al., 1940, 1942). This polysaccharide was later found to be a β‐1,2‐glucan polymer with a cyclic form (Putman et al., 1950; Zevenhuizen & Scholten‐Koerselman, 1979). Enzymes for its synthesis were independently identified as cyclic β‐1,2‐glucan synthases (CGSs) from *Brucella abortus*, *Sinorhizobium meliloti*, and *R. radiobacter* (Castro et al., 1996; Iannino et al., 1998; Nakajima, 2023; Zorreguieta et al., 1985; Zorreguieta & Ugalde, 1986). The overall reaction mechanism of CGSs has been studied biochemically using a CGS from *B. abortus* (Ciocchini et al., 2006, 2007).

In contrast to the enzymes for the synthesis of β‐1,2‐glucans, there had been no sequence‐identified β‐1,2‐glucan‐degrading enzymes until 1,2‐β‐oligoglucan phosphorylase (SOGP, “S” is derived from “sophorooligosaccharide,” an alternative name of β‐1,2‐glucooligosaccharide; see Note 1, Supporting Information S5 for abbreviation) was found in *Listeria innocua* in 2014 (Nakajima et al., 2014). Then, a method for the large‐scale production of β‐1,2‐glucans was established by taking advantage of SOGPs (Abe et al., 2015; Kobayashi et al., 2019; Nakajima et al., 2021). Further investigation of other β‐1,2‐glucan‐degrading enzymes has been performed using the produced glucans. endo‐β‐1,2‐Glucanases (SGLs) that release β‐1,2‐glucooligosaccharides (Sop_*n*_s, where *n* is the degree of polymerization [DP]) from β‐1,2‐glucans were identified from a bacterium and a fungus (Kitahata & Edagawa, 1987; Mendoza & Amemura, 1983; Reese et al., 1961) (see Note 1, Supporting Information S5 for abbreviation). These enzymes from the bacterium and the fungus were classified into the new GH families (GH144 and GH162, respectively) (Abe et al., 2017; Tanaka et al., 2019). Recently, the middle domain of CGS from *Thermoanaerobacter italicus*, a thermophilic bacterium (TiCGS) alone was found to catalyze a transglycosylation reaction. The finding of the transglycosylation domain of TiCGS (TiCGS_Tg_) led to the creation of GH189 (Tanaka et al., 2024). Exploration of SGL and SOGP gene clusters resulted in findings of various β‐1,2‐glucans‐associated enzymes with new functions and structures (Abe et al., 2018; Ishiguro et al., 2017; Kobayashi et al., 2022; Nakajima et al., 2016, 2017; Shimizu et al., 2018). However, considering the wide distribution of β‐1,2‐glucan in nature, understanding of β‐1,2‐glucans‐associated enzymes is far from obtaining the whole picture of the distribution of the related enzymes.

SGLs from *Chitinophaga pinensis* (CpSGL) and *Talaromyces funiculosus* (TfSGL), founding members of GH144 and GH162, respectively, show only approximately 10% amino acid sequence identity (Abe et al., 2017; Tanaka et al., 2019). However, CpSGL and TfSGL share a similar overall domain fold, indicating a relationship between the molecular evolution of these families. GH144 and GH162 are categorized into a new GH clan, clan GH‐S, according to classification based on structural similarity (Tanaka et al., 2024). Homology search using CpSGL and TfSGL as queries revealed that several groups were further found around the GH144, GH162, and GH189 families. These newly found groups, along with GH144, GH162, and GH189, formed a huge phylogenetic group. In the present study, the huge group is called “SGL clan.” We characterized homologs in the newly found groups (new GH families) biochemically and structurally and found the complicated molecular evolution associated with changes in reaction mechanisms.

## 2 RESULTS

### 2.1 Phylogenetic analysis of the SGL clan

**FIGURE 1:**
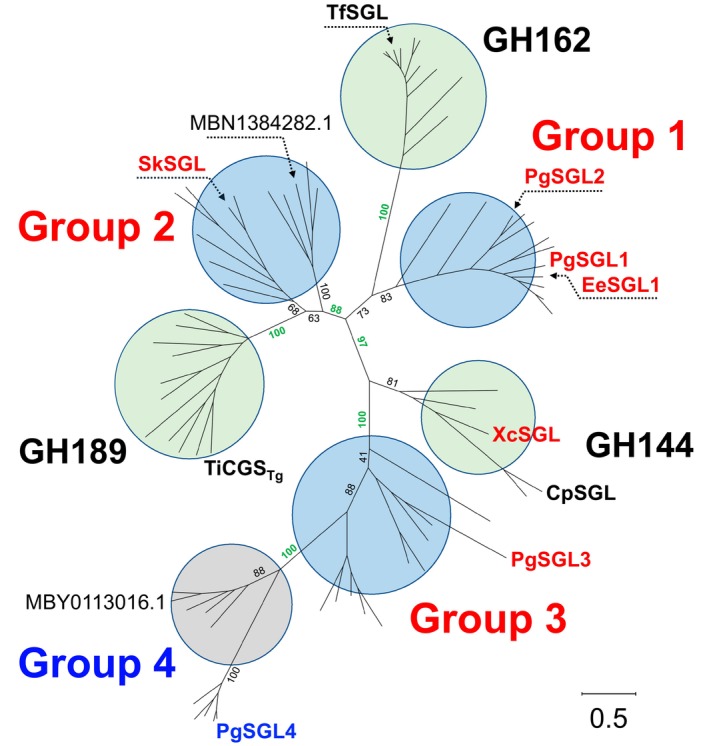
Phylogenetic tree of proteins in the β‐1,2‐glucanase (SGL) clan with bootstrap confidence levels. The proteins used in this study are labeled in the tree. TfSGL, CpSGL, and TiCGS_Tg_ with known biochemical functions are shown in bold black letters. The enzymes with biochemical functions that were elucidated in the present study are shown in bold red letters. PgSGL4 is shown in bold blue letters as an unknown function protein after biochemical investigation. Known glycoside hydrolase (GH) families (GH144, GH162, and GH189) and newly established GH families are shown as light green and light blue circles, respectively. A group with an unknown function is indicated by the light gray circle. Bootstrap confidence levels are shown as percentages at the main branches to distinguish each group. The values important for showing the boundary between groups are shown in bold green numbers. A scale bar indicating the phylogenetic distance is shown at the bottom right. Multiple sequence alignment was performed by MUSCLE (Edgar, 2004) and the phylogenetic tree was created using MEGA11 (Tamura et al., 2021).

To understand the evolutionary relationships between GH144, GH162, and GH189, a PSI‐BLAST search was performed using CpSGL (GH144), TfSGL (GH162), and TiCGS_Tg_ (GH189) as queries. Many collected homologs showed very low amino acid sequence identity with CpSGL, TfSGL, and TiCGS_TG_. Because these homologs do not belong to families GH144, GH162, or GH189, PSI‐BLAST searching was repeated until new homologs could no longer be obtained. To decrease the number of homologs appropriately for further analysis, homologs were extracted so as not to show more than a certain amino acid sequence identity with others; the identity cut‐off values were set so that the number of homologs did not exceed 250 in each group, which was classified provisionally based on amino acid sequence identity and preliminary phylogenetic analysis (see Section 4, Data S1, S2 for details). After 5–12 homologs were selected sparsely in each group, a phylogenetic tree with bootstrap confidence was constructed to investigate the phylogenetic relationships between the selected homologs. Consequently, four groups of proteins of unknown function were visualized around GH144, GH162, and GH189, and these groups were named Groups 1–4 (Figure 1, Data S3, S4). The collection of Groups 1–4, GH144, GH162, and GH189 forms the “SGL clan” as defined above. Amino acid sequence identities between the representative homologs used for the biochemical and structural analyses described below are shown in Figure S1A. Most of the values were very low (<20%). Based on bootstrap values (≥88%), reliable separations were found at the nodes marked with bold green numbering in Figure 1. For example, the value “100” to the right of GH189 indicates that GH189 was distinguished completely from the other families and groups. The combination of these separations suggests that the SGL clan is divided into individual groups and families, as shown in Figure 1.

GH189 and GH144 are one of the largest groups in the SGL clan based on the numbers of homolog hits. GH144 and GH162 are much larger families than registered in the CAZy database as of Jan 2025. While the GH162 proteins are distributed almost exclusively in eukaryotes, the homologs in the SGL clan, except for GH162, are distributed widely in bacteria, including uncultured bacteria. Although the species were not clearly separated by the phylogenetic groups, Group 2 proteins were mainly distributed in Gram‐positive bacteria. Groups 1 and 3 contained proteins from many marine bacteria, for example, isolated from marine sediment and mudflat environments (Storesund & Øvreås, 2013; Vitorino & Lage, 2022; Wiegand et al., 2020). Group 1 was a smaller group than Groups 2 and 3, and Group 4 was the smallest in the SGL clan based on the number of collected homologs (see Section 4 for detail).

### 2.2 Initial biochemical characterization of representative homologs from different new groups

In this study, we used PgSGL1, PgSGL2 from *Photobacterium gaetbulicola* and EeSGL1 from *Endozoicomonas elysicola* as Group 1 proteins; SkSGL from *Sanguibacter keddieii*, a Gram‐positive bacterium (Ivanova et al., 2009), as a Group 2 protein; PgSGL3 and PgSGL4 from *P. gaetbulicola* as Groups 3 and 4 proteins, respectively. GH144 protein from *Xanthomonas campestris* pv. *campestris* (XcSGL) was also used (see Note 2, Supporting Information S5, Section 4, Figure S2, Table S1 for detail). Because hydrolytic activities toward β‐1,2‐glucans were detected for PgSGL1, PgSGL2, PgSGL3, EeSGL1, SkSGLc, and XcSGL, the general properties of these enzymes were investigated using β‐1,2‐glucan alditols (see Section 4 for details) as substrates. While many of the enzymes showed optimal pH values around neutral pH, the optimal pH values of PgSGL3 and XcSGL were 8.5 and 5.0, respectively, which suggests that the optimal pH value varied within the SGL clan (Figures S3–S8). These enzymes were stable at pH ranges including neutral pH, which was consistent with the results for the optimal pH values. The optimal temperatures were 30–50°C, and the enzymes were stable at least up to 30°C. The assay for PgSGL2 was performed in the presence of NaCl because PgSGL2 lost its activity without NaCl. The activity of PgSGL2 increased with increasing concentrations of NaCl in the reaction solution up to 500 mM (Figure S9A). To maintain the maximum activity of PgSGL2, at least 250 mM NaCl was needed in the PgSGL2 solution (Figure S9B). PgSGL4 did not show activity toward β‐1,2‐glucans (DP121) nor Sop_2–5_ at pH 7.0 (data not shown). Size‐exclusion chromatography was performed using PgSGL1, PgSGL3, and SkSGLc. PgSGL1 and PgSGL3, single‐domain enzymes, were eluted at the time corresponding to 42 kDa, while the elution time of SkSGLc corresponded to 140 kDa (Figure S10). These results indicated that PgSGL1 and PgSGL3 were monomeric enzymes and SkSGL was a dimeric enzyme. The N‐ and C‐terminal domains in SkSGL may be involved in the dimer formation.

### 2.3 Characteristics of the SGLs in the new groups

**FIGURE 2:**
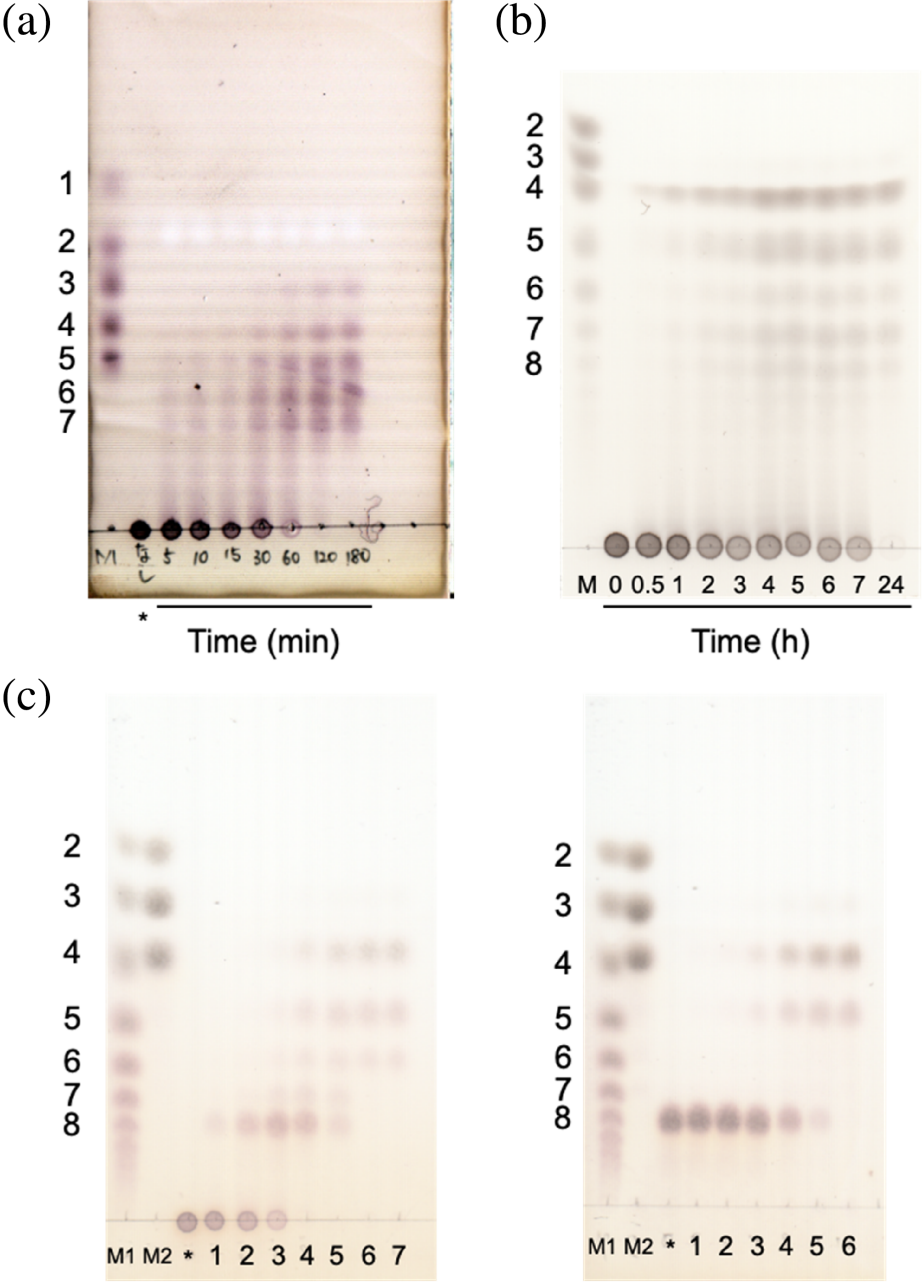
Action pattern analysis using TLC. (a) EeSGL1 (Group 1); (b) SkSGLc (Group 2); (c) PgSGL3 (Group 3). Asterisks indicate the reaction mixture without enzymes. Lanes M, M1, and M2 show the markers. (a) The reaction times are written directly on the TLC plate. Lane M, 0.2 μL of solution containing 0.5% each of Glc, Sop_2_, Sop_3_, Sop_4_, and Sop_5_. The reaction solutions (0.5 μL) were spotted on the plate. (b) Lane M, 0.5% Sop_*n*_s mixture prepared as described in Section 4. A 1‐μL aliquot of a marker or sample was spotted on the plate. (c) Lane M1, 1% Sop_*n*_s mixture prepared as described in Section 4. Lane M2, a mixture of Sop_2_ (10 mM), Sop_3_ (7.5 mM), and Sop_4_ (5 mM) was spotted. A 0.7‐μL aliquot of a marker or sample was spotted on the plate. Lanes 1–5, the reactions were performed with 0.025, 0.05, 0.1, 0.25, and 0.5 mg/mL PgSGL3, respectively, at 30°C for 20 min. Lanes 6–7 (left), the reactions were performed with 1 mg/mL PgSGL3 at 30°C for 20 and 220 min, respectively. Lane 6 (right), the reaction was performed with 1 mg/mL PgSGL3 at 30°C for 30 min.

**FIGURE 3:**
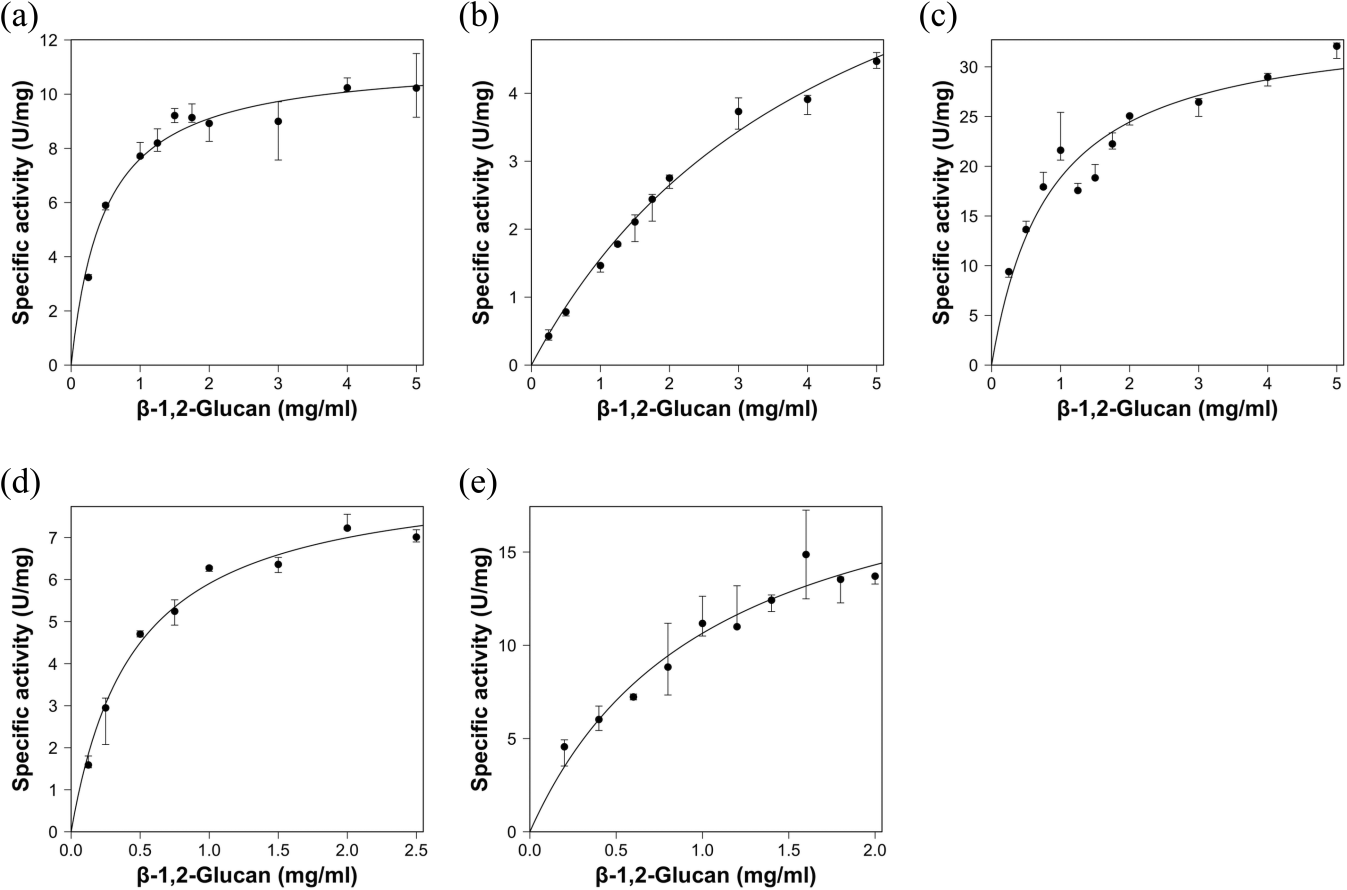
Kinetic analysis of the enzymes in the β‐1,2‐glucanase (SGL) clan. Group 1, (a) PgSGL1, (b) PgSGL2, (c) EeSGL1; Group 2, (d) SkSGL; and Group 3, (e) PgSGL3. Medians are plotted as closed circles. The other data in the triplicate experiments are shown as bars. In the case EeSGL1, two data points (1.25 and 1.5 mg/mL substrate) were eliminated from the graph because they were obviously outliers.

The substrate specificities of the six enzymes toward various polysaccharides were investigated. All the enzymes were specific for β‐1,2‐glucans (Table 1). The reaction patterns of these enzymes were then investigated using β‐1,2‐glucans as substrates by thin layer chromatography (TLC) analysis. EeSGL1 (Group 1) produced Sop_3–7_ endolytically (Figure 2a), as did PgSGL1 and PgSGL2 (data not shown). SkSGLc (Group 2) released Sop_4_ mainly in the initial phase of the reaction and produced Sop_4–8_ as the final products (Figure 2b). Interestingly, PgSGL3 (Group 3) produced Sop_8_ predominantly in the initial stage of the reaction, and then Sop_8_ was further hydrolyzed (Figure 2c, left). When Sop_8_ was used as a substrate, Sop_8_ was hydrolyzed to Sop_3–5_ (Figure 2c, right). All the enzymes were found to be endolytic enzymes, as is CpSGL (Abe et al., 2017). Overall, in the SGL clan, the substrate specificity was the same, but the degradation patterns were different. Kinetic analysis of PgSGL1, PgSGL2, PgSGL3, EeSGL1, and SkSGLc was performed (Figure 3). All examined enzymes showed sufficiently large *V*_max_ values and sufficiently small *K*_m_ values as GH enzymes (Table 2). The five enzymes (Groups 1–3) were clearly identified as SGLs. XcSGL can be regarded as an SGL because of its narrow specificity for β‐1,2‐glucans and a similar level of specific activity toward β‐1,2‐glucans as the other SGLs.

**TABLE 1:** Substrate specificity of enzymes in the β‐1,2‐glucanase (SGL) clan.

Substrate	Final concentration (%)	Substrate specificity (U/mg)					
		Group1			Group 2	Group 3	GH144
		PgSGL1^e^	PgSGL2	EeSGL1^e^	SkSGLc	PgSGL3a, b^e^	XcSGL
β‐1,2‐Glucan alditol	0.2	8.44 (0.92)^a^	3.13 (0.41)	25.4 (3.0)	12.3 (1.0)	8.96 (0.85)	3.85 (0.14)
CM‐cellulose	0.1	N.D.^c^	N.D.	N.D.	N.D.	N.D.	N.D.
Barley β‐glucan	0.2	N.D.	N.D.	N.D.	N.D.	N.D.	N.D.
Glucomannan	0.05 (0.1)^b^	N.D.	N.D.	N.D.	N.D.	N.D.	N.D.
Tamarind xyloglucan	0.2	N.D.	N.D.	N.D.	N.D.	N.D.	N.D.
Lichenan	0.0125 (0.01)	N.D.	N.D.	N.D.	N.D.	N.D.	N.D.
Laminarin	0.0125 (0.01)	N.D.	N.D.	N.D.	N.D.	N.D.	N.D.
CM‐curdlan	0.05	N.D.	N.D.	N.D.	N.D.	N.D.	N.D.
Pustulan	0.05	N.D.	N.D.	N.D.	N.D.	N.D.	N.D.
Arabinogalactan	0.006 (0.01)	N.D.	N.D.	N.D.	N.D.	N.D.	N.D.
Arabinan	0.1 (0.2)	N.D.	N.D.	N.D.	N.D.	N.D.	N.D.
Polygalacturonic acid	0.0025 (0.01)	N.D.	N.D.	N.D.	N.D.	N.D.	N.D.
Xylan (Beechwood)	0.1	N.E.^d^	N.D.	N.E.	N.E.	N.E.	N.E.
Soluble starch	0.02	N.D.	<0.3%	N.D.	N.D.	N.D.	N.D.

^a^ The values in the parentheses after the specific activity represent the largest difference between the median value and the other data in triplicate experiments as errors.

^b^ The values in the parentheses after the final concentrations represent the substrate concentrations for the assay of EeSGL1.

^c^ N.D. indicates that the relative activity was lower than 0.2% of the activity toward β‐1,2‐glucan alditols.

^d^ N.E.: not examined.

^e^ Hydrolytic activities toward *p*‐nitrophenyl‐β‐glucopyranoside were investigated preliminarily only for PgSGL1, EeSGL1, and PgSGL3. No hydrolytic activity was detected (data not shown).

**TABLE 2:** Kinetic parameters of the homologs in the β‐1,2‐glucanase (SGL) clan.

	*V*_max_ (U/mg protein)	*K*_m_ (mg substrate/mL)	*V*_max_/*K*_m_ (U/mg protein mL/mg substrate)
This study			
(Group 1) PgSGL1	11.3 ± 0.4	0.484 ± 0.068	23.3 ± 2.6
PgSGL2	8.58 ± 0.71	4.48 ± 0.61	1.92 ± 0.11
EeSGL1	34.7 ± 2.5	0.842 ± 0.182	41.3 ± 6.4
(Group 2) SkSGLc	8.59 ± 0.34	0.455 ± 0.058	18.9 ± 1.8
(Group 3) PgSGL3	21.8 ± 2.5	1.05 ± 0.26	20.8 ± 2.9
References^a^			
(GH144) CpSGL^b^, ^c^	57 ± 4.7	0.83 ± 0.18	81 ± 12
(GH162) TfSGL^b^	33 ± 1.1	0.18 ± 0.02	180 ± 11
(GH186) OpgD from *Escherichia coli* ^b^	28.7 ± 2.0	8.6 ± 1.1	3.37 ± 0.198

^a^ The kinetic parameters of CpSGL, TfSGL, and OpgD from *E. coli* are cited from Abe et al. (2017), Tanaka et al. (2019) and Motouchi et al. (2023), respectively.

^b^ Kinetic parameters were evaluated using β‐1,2‐glucan alditol (DP25) for CpSGL and TfSGL, and β‐1,2‐glucan alditol (DP121) for OpgD from *E. coli*.

^c^ Assay was performed using the 4‐hydroxybenzhydrazide method (Nakajima et al., 2012).

**FIGURE 4:**
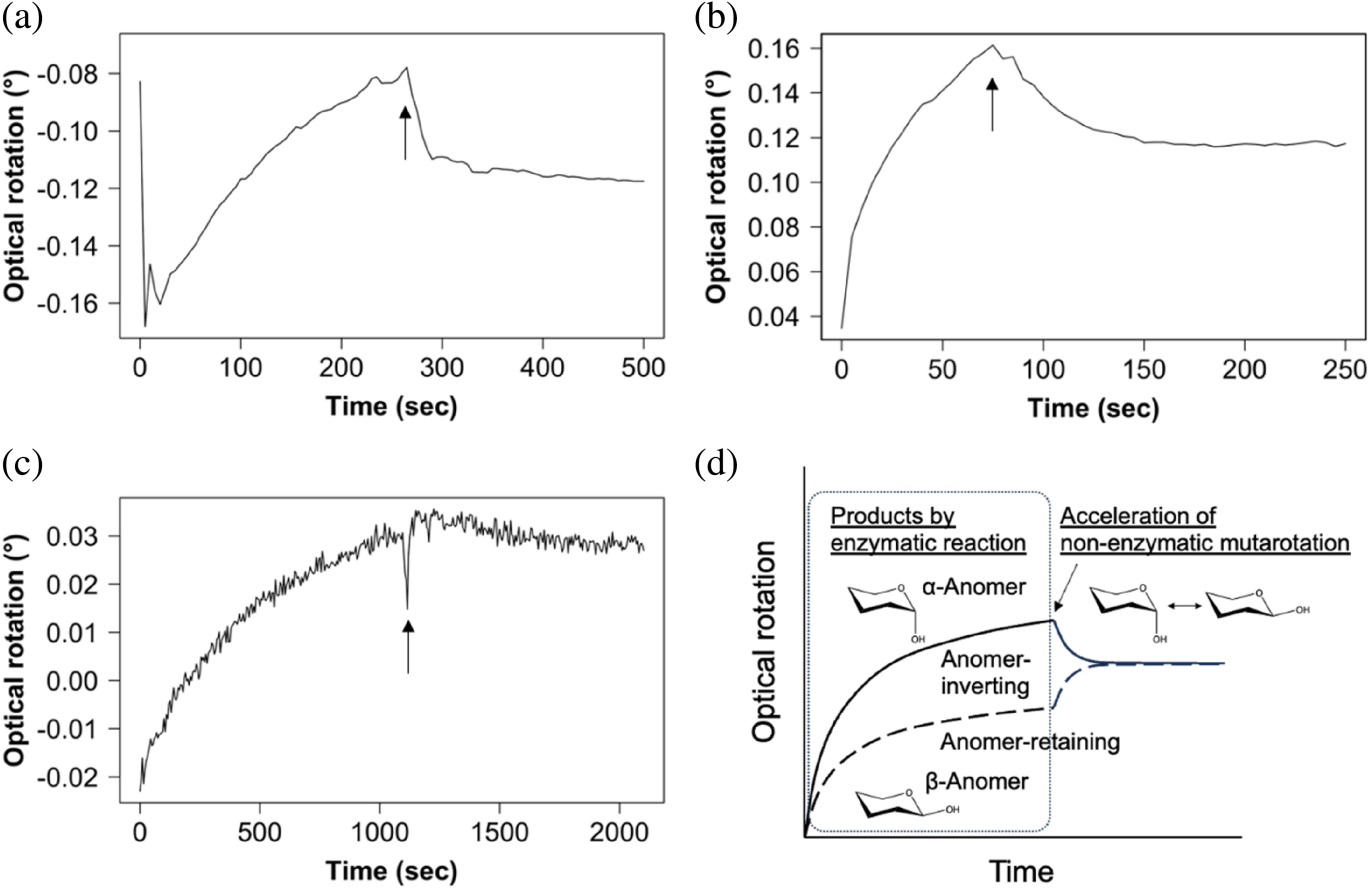
Time course of optical rotations during the hydrolysis of β‐1,2‐glucans (SGL). (a) PgSGL1 (Group 1), (b) PgSGL3 (Group 3), and (c) SkSGL (Group 2). The arrows indicate the time that aqueous ammonia was added to the reaction mixtures. (d) The assay principle of polarimetric analysis when using SGLs. A pattern shown as a solid line was found in CpSGL and TfSGL, anomer‐inverting SGLs (Abe et al., 2017; Tanaka et al., 2019). A dashed line represents a putative pattern of an anomer‐retaining SGL. During the enzymatic reaction boxed with a dotted line, products with either one anomer are produced. Representation of products with α‐ and β‐anomers is simplified. An arrow represents a time when aqueous ammonia is added. The alkalized solution stops the enzymatic reaction and accelerates non‐enzymatic mutarotation to reach equilibrium between anomers rapidly.

To determine the reaction mechanisms of the enzymes in Groups 1–3, that is, anomer‐retaining or anomer‐inverting, the time course of the optical rotation during the hydrolysis of β‐1,2‐glucans by PgSGL1, SkSGLn (see Section 4), and PgSGL3 was analyzed. This analysis is important to understand the fundamental characteristics that are shared in the new phylogenetic groups. The three enzymes (PgSGL1, PgSGL3, and SkSGLn) showed the same patterns as TfSGL and CpSGL (Abe et al., 2017; Tanaka et al., 2019), indicating that these three enzymes follow an anomer‐inverting mechanism (Figure 4).

### 2.4 Comparison of overall structures within the SGL clan

**FIGURE 5:**
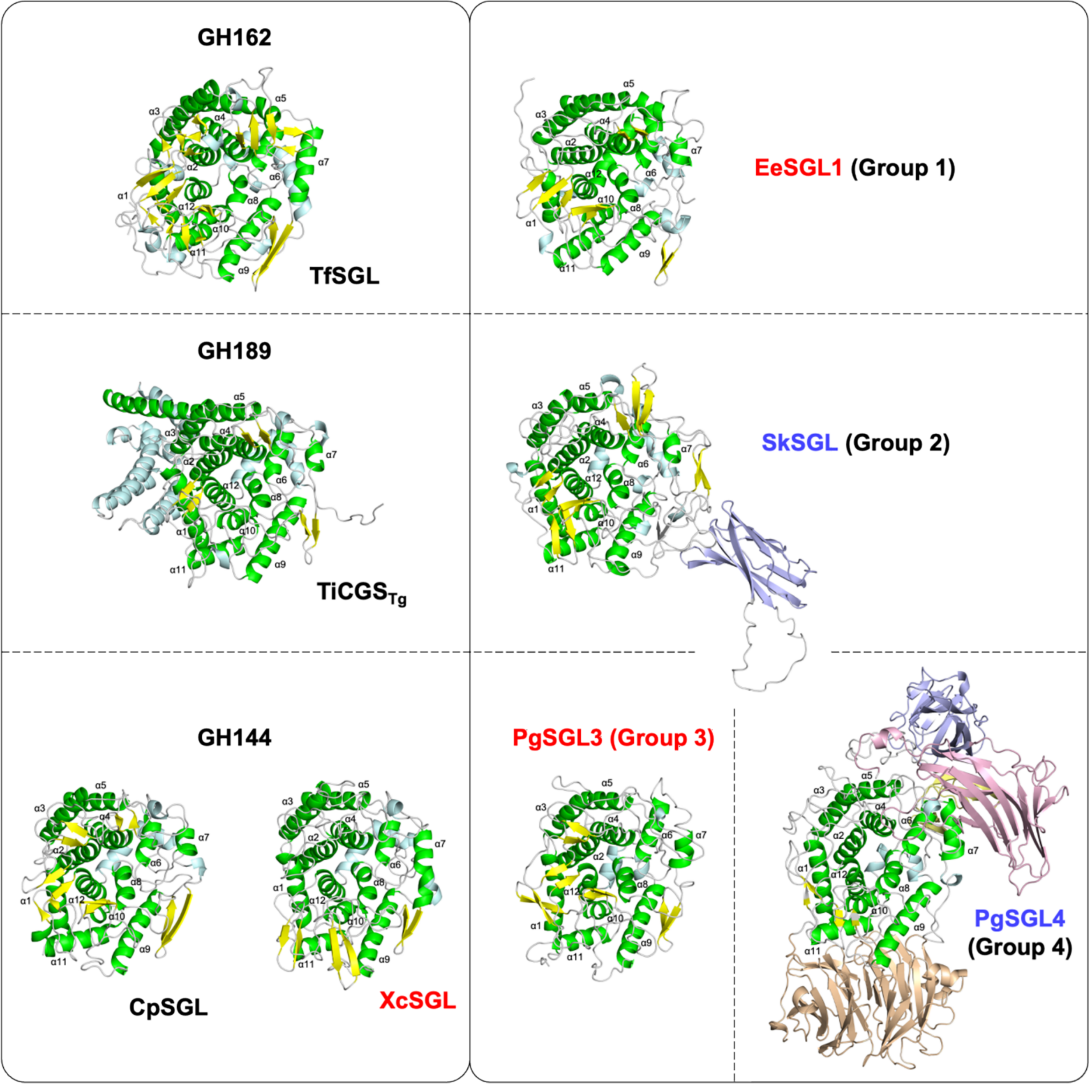
Overall structures of Groups 1–4, GH144, GH162, and GH189. Overall structures are shown as cartoons. α‐Helices that compose (α/α)_6_‐barrel domains and the other ones are colored in green and pale cyan, respectively. β‐Strands in (α/α)_6_‐barrel domains are shown in yellow. Additional domains are distinguished in light brown and light purple. Known glycoside hydrolase (GH) families and Groups 1–4 are enclosed on the right and left sides, respectively. Families and Groups are separated with dashed lines. The solved structures in this study and the predicted structures are labeled with red and blue letters, respectively.

Ligand‐free structures of EeSGL1 and PgSGL3 were obtained with 2.4 and 1.2 Å resolution, respectively (Table S2). A structure of the complex with Sop_7_ was obtained with 2.5 Å resolution using the catalytic domain of XcSGL (Table S2). The C‐terminal additional region of XcSGL is annotated as a disordered region according to the UniProt database (Table 3). Overall, the structures of these enzymes are composed of a single (α/α)_6_‐barrel domain, similar to CpSGL and TfSGL (Abe et al., 2017; Tanaka et al., 2019) (Figures 5 and S11). SkSGL and the catalytic domain of PgSGL4 were predicted using AlphaFold Colab based on AlphaFold v2.1.0 (https://colab.research.google.com/github/deepmind/alphafold/blob/main/notebooks/AlphaFold.ipynb) (Jumper et al., 2021). A predicted structure of PgSGL4 (AlphaFold Protein Structure Database [https://alphafold.ebi.ac.uk/], AF‐A0A0C5WQL5‐F1) (Varadi et al., 2022) was used as an overall structure. The conserved region in SkSGL also forms a single (α/α)_6_‐barrel domain (Figure 5, Table 3). The C‐terminal additional region forms an Ig‐like domain with a disordered region. This domain is unique to SkSGL in Group 2. PgSGL4 also has a single (α/α)_6_‐barrel domain along with an N‐terminal six‐bladed β‐propeller domain and a motif composed of β‐strand and two putative carbohydrate‐binding domains at the C‐terminus (Figure 5, Table 3).

**TABLE 3:** Basic information of the target proteins.

	*N*‐terminal signal peptide (a.a.)	Number of residues (a.a.)	Domain organization^d^
PgSGL1	None	440	(α/α)_6_‐barrel
PgSGL2	None	454	(α/α)_6_‐barrel
EeSGL1	1–22^a^	438	(α/α)_6_‐barrel
PgSGL3	1–21	440	(α/α)_6_‐barrel
SkSGLc^b^	1–32 (28–59)^c^	724 (751)^c^	36–508 (63–535)^c^, (α/α)_6_‐barrel 520–631 (547–658)^c^, Ig‐like
XcSGL	1–27 (13–39)^c^	545 (557)^c^	55–477 (67–489)^c^, (α/α)_6_‐barrel 491–545 (503–557)^c^, disordered
PgSGL4	1–24	1142	43–344, six‐bladed β‐propeller^e^ 345–739, (α/α)_6_‐barrel 740–801, motif composed of β‐strands 830–966, putative carbohydrate‐binding domain 967–1142, putative carbohydrate‐binding domain

^a^ SignalP4.1 was used to predict an N‐terminal signal peptide.

^b^ Because of a sequence error (deletion of the cytosine at position 123) in the database, the corrected sequence was used.

^c^ A signal peptide region was obscure when the first methionine was adopted as an amino acid encoded by a start codon. Thus, the second methionine was adopted instead. The numbering starts at the second methionine. The numbers in parentheses indicate the residue numbers when the first methionine was adopted as a start codon.

^d^ The whole regions except *N*‐terminal signal peptides were used for expression. The regions of domains are shown in residue numbers. Domain organization is annotated basically according to the UniProt database (https://www.uniprot.org/) (Bateman et al., 2021) and InterPro (https://www.ebi.ac.uk/interpro/) (Jones et al., 2014; Paysan‐Lafosse et al., 2023).

^e^ Annotation of the domain is confirmed visually.

The root‐mean‐square deviation (RMSD) values between the proteins in the groups of the SGL clan are <3.5 Å according to a homology search using the DALI server (http://ekhidna2.biocenter.helsinki.fi/dali/) (Holm, 2020, 2022) (Figure S2B), suggesting that the overall structures of the proteins within the clan are similar. In particular, the overall appearances of the catalytic domains in phylogenetically close groups (GH162 and Group 1; GH189 and Group 2; GH144 and Group 3) are similar (Figure 5). Although Group 4 is phylogenetically far from GH144, the overall shape of the (α/α)_6_‐barrel domain in PgSGL4 is also similar to those in CpSGL and XcSGL (GH144 enzymes).

**FIGURE 6:**
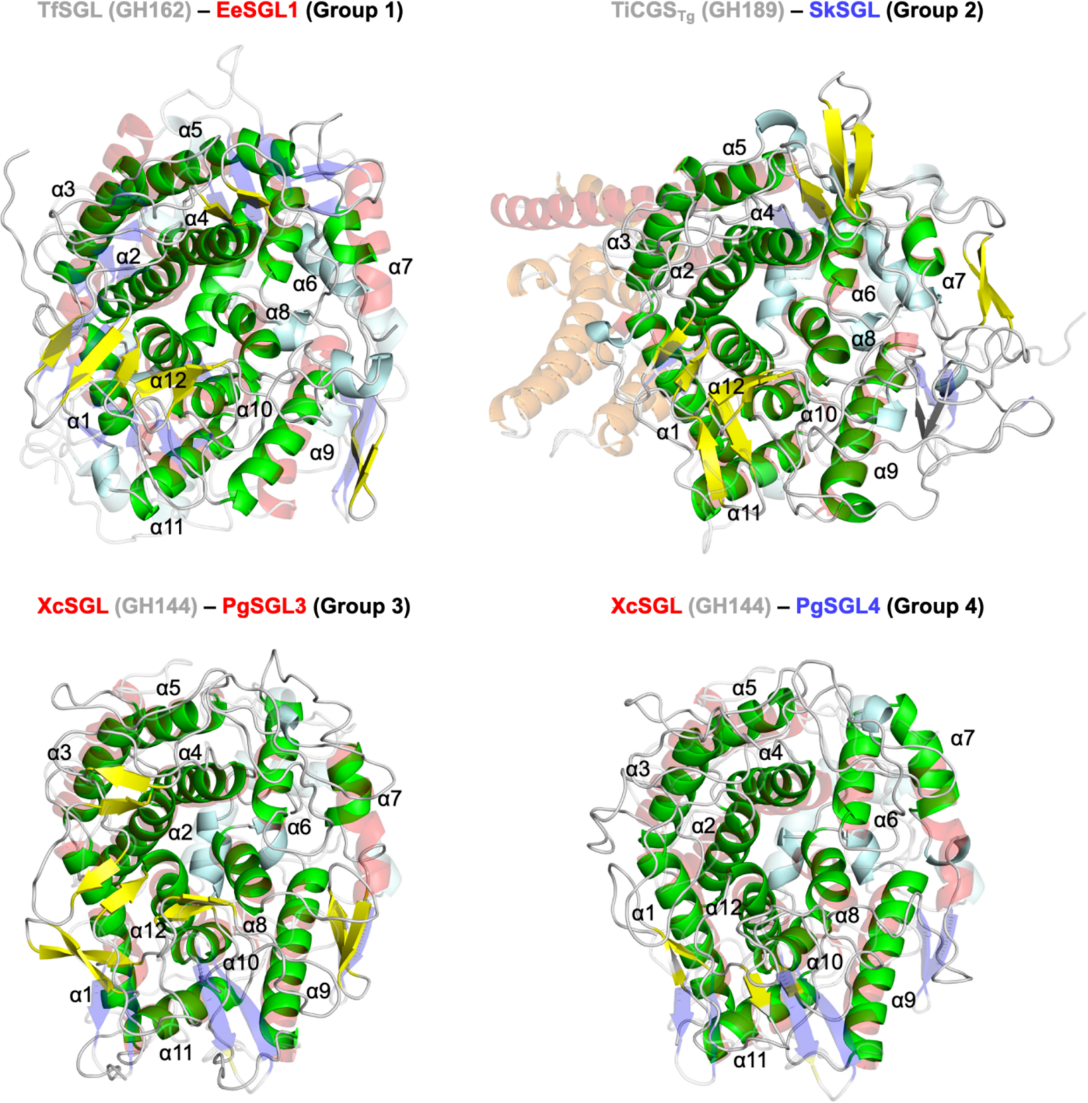
Comparison of overall structures. The structures solved experimentally in this study, the predicted structures, and the structures that were registered in Protein Data Bank previously are labeled with red, blue, and black letters, respectively. The known families (GH144, GH162 and GH189) are labeled in gray. α‐Helices composing (α/α)_6_‐barrel domains are numbered with α1 to α12 from N‐termini. XcSGL, TfSGL, and TiCGS_Tg_ are shown as semi‐transparent cartoons. α‐Helices (α1–α12) and the other α‐helices in these enzymes are shown in red and pale cyan, respectively. β‐Strands and loops are shown in blue and white, respectively. α‐Helices (α1–α12), the other α‐helices, β‐sheets, and loops are shown as green, pale cyan, yellow, and white cartoons, respectively.

To understand the differences in the overall structures in detail, (α/α)_6_‐barrel domains were superimposed in pairs (Figure 5). Although the positions of α‐helices are similar between TfSGL (GH162) and EeSGL1 (Group 1), differences in orientations (α3 and α9) and lengths (α7 and α12) are apparent visually (Figure 6). Positions and orientations of α‐helices are almost conserved between TiCGS (GH189) and SkSGL (Group 2). However, several remarkable differences were found. TiCGS has a particularly long α5 helix and several additional α‐helices between α4 and α5. This feature is conserved only within GH189 and clearly distinguishes Group 2 from GH189. In the case of XcSGL (GH144) and PgSGL3 (Group 3), the lengths, orientations, and locations of α‐helices are similar, except for the lengths of helix α7. The comparison between XcSGL (GH144) and PgSGL4 (Group 4) can be described in almost the same way as that between GH144 and Group 3, although the deviation of the α‐helices appears to be slightly greater than that between GH144 and Group 3. Other superimposed pairs are shown in Figure S11. Unlike the other combinations, TfSGL (GH162) looks rather different from the proteins in Groups 2–4 in the positions of the α‐helices, which is consistent with the RMSD values shown in Figure S11. This may be related to the fact that almost all the GH162 proteins are of eukaryotic origin.

### 2.5 Complex structures of the SGLs in the SGL clan

**FIGURE 7:**
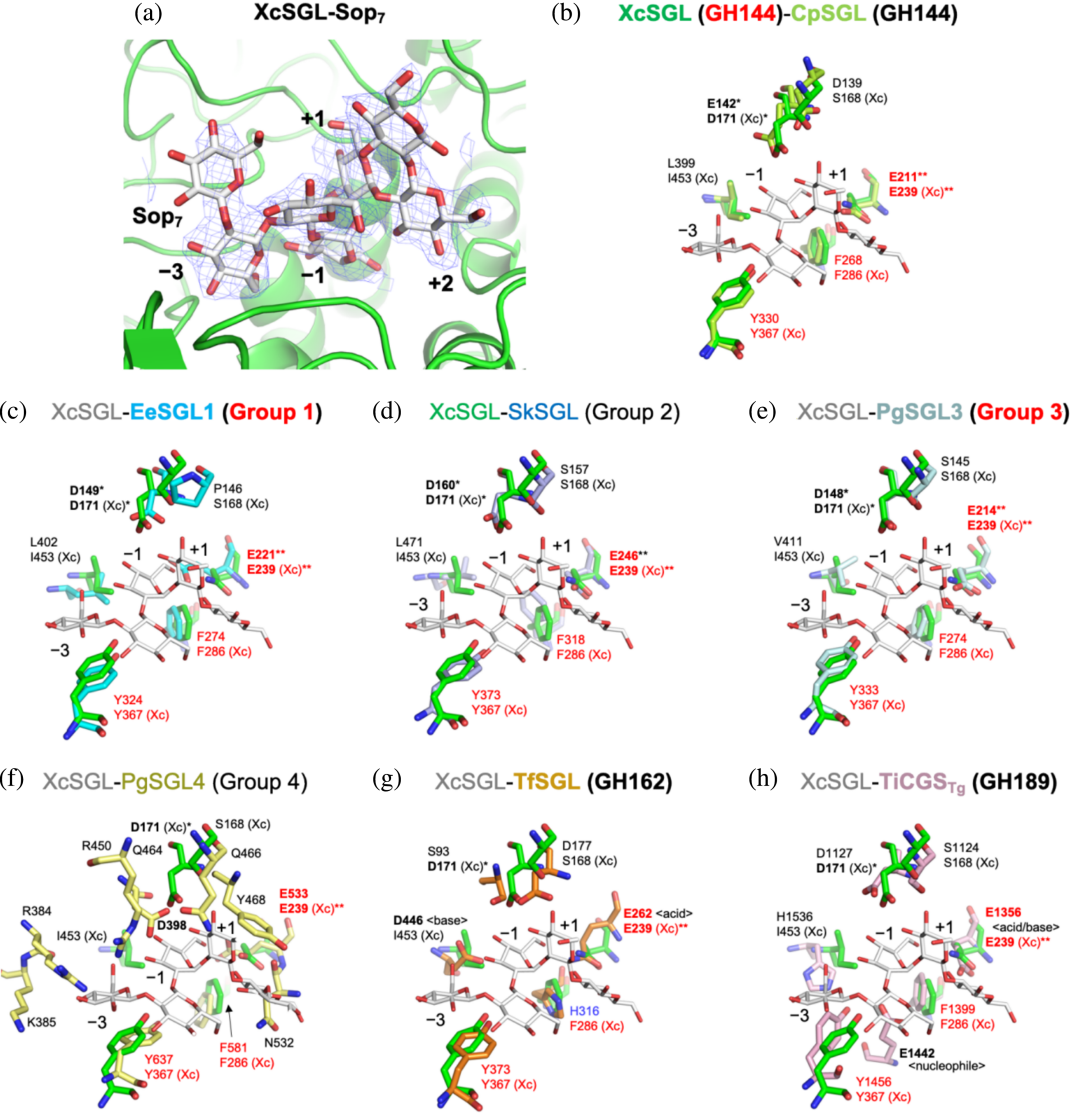
Comparison of the substrate pockets of β‐1,2‐glucanase (SGLs) in the SGL clan. (a) The complex structure of XcSGL with Sop_7_. The *F*_o_ − *F*_c_ omit map for Sop_7_ is shown at the 2.0*σ* contour level and is represented as a blue mesh. (b–h) XcSGL (Protein Data Bank [PDB] ID, 8XUL) is superimposed with CpSGL (PDB ID, 5GZK) (b), EeSGL1 (PDB ID, 8XUJ) (c), SkSGL (prediction) (d), PgSGL3 (PDB ID, 8XUK) (e), PgSGL4 (prediction) (f), TfSGL (PDB ID, 6IMU) (g), and TiCGS_Tg_ (PDB ID, 8WY1) (h). Experimentally solved structures are labeled in bold, except for XcSGL in (c–h), and with bold red GH or group names. Sop_7_ molecules are represented as white sticks. The colors for each protein are as follows: XcSGL, green; CpSGL, light green; EeSGL1, cyan; SkSGL, light purple; PgSGL3, pale cyan; PgSGL4, pale yellow; TfSGL, orange; and TiCGS_Tg_, light pink. The residue numbers of XcSGL and SkSGL are based on the starting methionine residue defined in this study (see Table 3 for detail). (f) PgSGL4 was aligned with CpSGL by pair fitting using E211, F268, and Y330 in CpSGL. (g) TfSGL was aligned with CpSGL using ligands. Sop_7_ in TfSGL is shown as a thin orange stick. The residues that define the SGL clan (E239, F286, and Y367 in the case of XcSGL) are colored red. The H316 in TfSGL is colored blue. Catalytic residues and candidates for catalytic residues are shown in bold letters. (Xc) represents the residues in XcSGL. Functions of catalytic residues identified in a previous studies (Tanaka et al., 2019, 2024) are shown in angle brackets. Each candidate general base suggested in this study is labeled with one asterisk. Each general acid proposed in this study is labeled with two asterisks.

Among the SGL clan, we obtained a complex structure of GH144 XcSGL E239Q mutant (a catalytic residue mutant) with a Sop_7_ molecule (Figure 7a). The fourth Glc unit from the non‐reducing end forms a skew boat (^1^ *S*_3_) conformation, suggesting that this Glc unit is located at subsite −1. The conformation of the Sop_7_ molecule is superimposed with that of TfSGL and both enzymes show similar shapes of the substrate pockets (Figure S12). Although the substrate‐binding structures of EeSGL1, SkSGL, and PgSGL3 could not be determined experimentally, the shapes of the substrate pockets in these enzymes are similar to those of TfSGL and XcSGL in that the Sop_7_ molecules in TfSGL and XcSGL are accommodated in the pockets without obvious steric hindrance. Therefore, we generated substrate‐binding structures computationally using molecular dynamics (MD) simulations (Figure S13). In the final structures of the MD simulations with Sop_8_ as a substrate, the substrate is well‐fitted to the pockets of EeSGL1 and PgSGL3 without appreciable structural changes. The same is true for SkSGL predicted by AlphaFold2. In EeSGL1 and SkSGL, the 3‐OH group of the Glc moiety at subsite +2 is oriented to the scissile bond oxygen atom. A proton in the carboxy group of the glutamic acid residue (EeSGL1, E221; SkSGL, E246) is located in the vicinity of the 3‐OH group (Figure S14A,B). These orientations are appropriate for the reaction. Contrarily, such appropriate orientations were not obtained in PgSGL3 during the MD simulation (Figure S14C). This might be because of a lack of simulation time or some difference in a substrate conformation required for the reaction between PgSGL3 and the other SGLs.

### 2.6 Conservation of residues among the SGL clan

The conservation of residues in each group was investigated using ConSurf (Ashkenazy et al., 2016; Landau et al., 2005). The residues forming the substrate pockets are highly conserved in each group, except Group 4 (Figure S15), suggesting that the substrate specificities and reaction mechanisms are the same in each group. However, the residues involved in substrate recognition are diversified across the groups in the SGL clan (Figure S16). According to the structure of PgSGL4 predicted by AlphaFold2 (Jumper et al., 2021), the substrate pocket of PgSGL4 is smaller than those of the other enzymes investigated in the present study and is too small to accommodate Sop_7_, unlike in the complexes of Sop_7_ with XcSGL and TfSGL (Figures S12 and S15). This observation is consistent with the absence of any activity of PgSGL4 toward β‐1,2‐glucans.

### 2.7 Mutational analysis of candidates for catalytic residues

In order to identify the catalytic residues in the newly found Groups, E221 and D149 in EeSGL1 (Group 1), and E214 and D148 in PgSGL3 (Group 3) were selected as candidate residues (see Discussion for detail). All four mutants (E221Q and D148N in EeSGL1, E214Q and D149N in PgSGL3) showed considerably decreased hydrolytic activity toward β‐1,2‐glucan (less than 0.1% of the specific activity of the wild‐type). This result suggests that the four residues are catalytic residues.

## 3 DISCUSSION

### 3.1 Conserved residues defining the SGL clan

**FIGURE 8:**
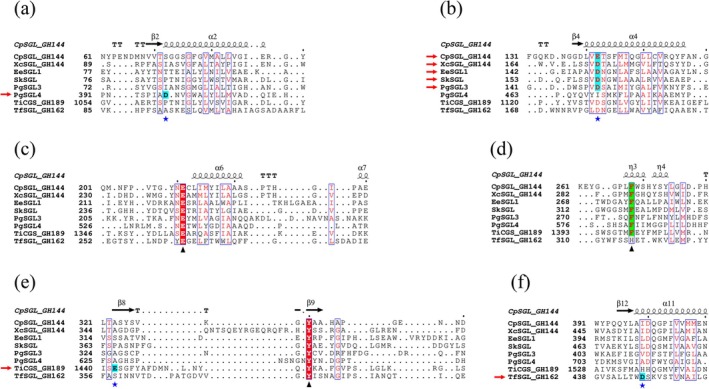
Multiple sequence alignment of the β‐1,2‐glucanase (SGL)‐clan proteins. Black triangles represent highly conserved residues in the SGL clan, except that the phenylalanine is replaced with a histidine in GH162. The phenylalanine residues indicated by the triangle in (d) are also highlighted in green. The black triangle in (c) represents the position of a nucleophile in TiCGS_Tg_ and (candidate) base catalysts for the other proteins. Residues where blue stars and red arrows intersect are candidates for acid catalysts. These residues are also highlighted in cyan. Multiple alignment was performed using the SALIGN server (Braberg et al., 2012). The ESPript 3.0 server (Robert & Gouet, 2014) was used for visualization of the alignment.

A combination of multiple sequence alignments and superimposition of tertiary structures highlighted the important residues shared throughout the SGL clan (Figures 7 and 8, Table S3). These residues are E239, Y367, and F286 in XcSGL (GH144) as indicated with black triangles in Figure 8. E239 forming bifurcated hydrogen bonds with a Glc unit at subsite +2 corresponds to E262 in TfSGL (GH162), which is an acid catalyst. Y367 forms a stacking interaction with a Glc moiety at subsite −3. F286 is a residue located close to a potential nucleophilic water. Although F286 is substituted with a histidine residue in GH162 (H316 in TfSGL) (Tanaka et al., 2019), GH162 is the only family including proteins mostly from eukaryotes. Thus, F286 is conserved among homologs from prokaryotes in the SGL clan. Overall, the residues corresponding to E239, Y367, and F286 in XcSGL can be used to define proteins of the SGL clan. In addition, such conservation is adopted in Group 4 although its biochemical function is unknown.

These facts suggest that the binding of a substrate at both subsites −3 and +2 is required for the reaction. The two subsites probably contribute to the compensation of disadvantage in binding due to the distortion of a Glc unit at subsite −1. In addition, a Glc unit at subsite −2 is poorly recognized in XcSGL and CpSGL (Figure 7a,b) (Abe et al., 2017). Such binding mode also suggests that the SGLs in this study cannot release a Glc unit from the non‐reducing end exolytically.

### 3.2 General acid in the SGL clan

The similarities and differences in the reaction mechanisms between Groups 1–3 and previously reported families (GH144, GH162, and GH189) are important to understand molecular evolution in the SGL clan. Generally, two acidic amino acids participate in reactions as catalytic residues in GH enzymes. Two catalytic residues in anomer‐inverting enzymes are a general acid and a general base. A general acid residue interacts with a glycosidic bond oxygen atom directly, and a general base interacts with a nucleophilic water directly in a typical anomer‐inverting enzyme. However, the new group enzymes in the SGL clan are unlikely to follow the typical mechanisms. Thus, we focus on general acid and general base for discussion here and the next section, respectively.

**FIGURE 9:**
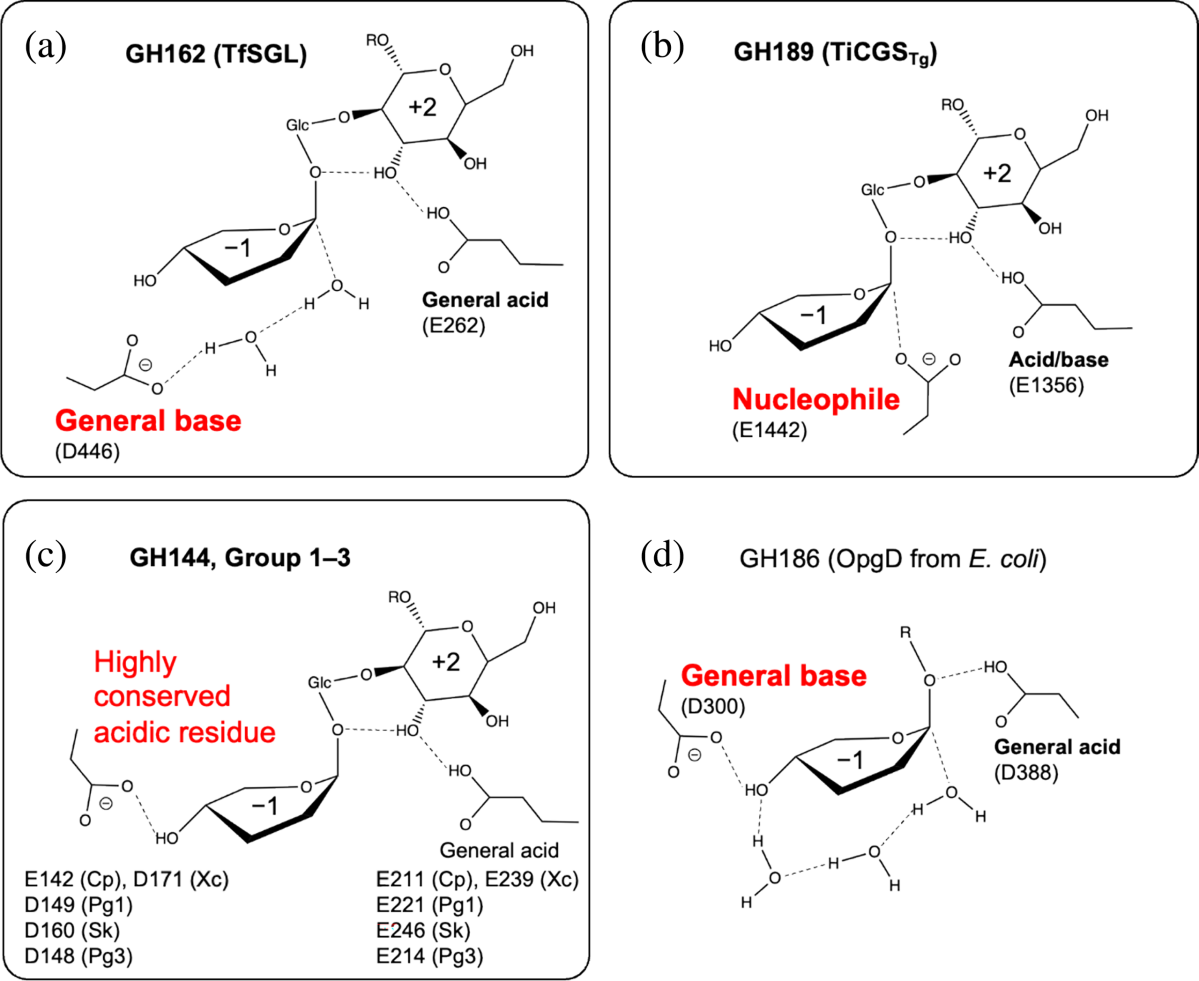
Reaction mechanisms of the β‐1,2‐glucanase (SGL) clan. Reaction mechanisms are shown schematically. (a–d) show different reaction pathways to attack the anomeric carbon at subsite −1. A nucleophile and (potential) general bases are shown in red letters to indicate the diversity in the SGL clan. The determined catalytic residues are shown in bold letters. Glucose (Glc) moieties at subsite +1 in the SGL clan are indicated by Glc. R represents a β‐1,2‐glucan moiety. Glc moieties at subsite −2 and beyond are omitted. Some substituents of the Glc moiety at subsite −1 are also omitted. (a, b) The reaction mechanisms of TiCGS_Tg_ (a) and TfSGL (b). (c) Perspective of the reaction mechanisms of GH144 and Groups 1–3. Cp, Xc, Pg1, Sk, and Pg3 are short representations of CpSGL (GH144), XcSGL (GH144), PgSGL1 (Group 1), SkSGL (Group 2) and PgSGL3 (Group 3). (d) The reaction mechanism of OpgD from *Escherichia coli*. Groups in the SGL clan are bordered with solid lines (a–c).

Among anomer‐inverting SGLs in the SGL clan, the reaction route has been identified only in TfSGL (GH162) (Figure 9a) (Tanaka et al., 2019). This is a substrate‐mediated route that the 3‐OH group at subsite +2 participates in. Although the reaction route of TiCGS_Tg_ (GH189) has also been identified as shown in Figure 9b, TiCGS_Tg_ is an anomer‐retaining enzyme (Tanaka et al., 2024). E262 in TfSGL (GH162), which is identified as a general acid catalyst, is conserved sequentially and spatially in the other groups in the SGL clan as described in the above Discussion section (Figures 7, 8, and 9a, Table S3). This residue provides a proton to the oxygen atom in the glycosidic scissile bond through the 3‐hydroxy group of the Glc moiety at subsite +2 (Tanaka et al., 2019) (Figures 7g and 9a). In EeSGL1 (Group 1) and PgSGL3 (Group 3), the corresponding residues are E221 and E214, respectively (Figure 7c,e), and both the E221Q mutant (EeSGL1) and E214Q mutant (PgSGL3) had considerably decreased hydrolytic activity as described above. Although mutational analysis of E246 in SkSGL is not performed, the hydrogen atom of the 3‐hydroxy group of the Glc moiety at subsite +2 faces the glycosidic bond oxygen atom in SkSGL as in the case of EeSGL1, according to the MD simulations (Figure S14), which suggests that the reaction route from E246 is appropriate for the catalytic reaction in SkSGL. Substitution of the corresponding residues in CpSGL, TfSGL, and TiCGS_Tg_ (E211Q, E262Q, and E1356Q, respectively) also drastically reduces the catalytic activity (Abe et al., 2017; Tanaka et al., 2019, 2024). Thus, E221 (EeSGL1, Group 1), E246 (SkSGL, Group 2) and E214 (PgSGL3, Group 3) are considered to be general acid catalysts.

### 3.3 Comparison of general base within the SGL clan

In typical anomer‐inverting enzymes, a general base directly attracts a proton from a nucleophilic water molecule attacking an anomeric carbon atom at subsite −1. However, there are cases in which a general base is obscure, such as in GH6, which has an anomer‐inverting mechanism. In GH6, aspartic acid residues interacting directly with a nucleophilic water are highly conserved. The Grotthuss mechanism, proton transfer by a water network, was first proposed for GH families in the GH6 cellobiohydrolase from *Trichoderma reesei* (Koivula et al., 2002). Nevertheless, the reaction mechanism of GH6 is still controversial because the water network in the enzyme is open to solvent, which is considered to be unsuitable for transferring a proton to a specific position. Recently, it was proposed that an arginine residue paired with the aspartic acid residue participates in the reaction mechanism, although the evidence is preliminary (Tachioka et al., 2024).

In the case of the SGLs found in this study, no acidic residue potentially acting on a nucleophilic water molecule is found. Thus, candidate general base residues are discussed based on knowledge of reaction mechanisms of TfSGL (GH162) and CpSGL (GH144). Arginine residues are not discussed in this study because no arginine residue that can participate in a reaction route is found in the regions.

D446 acts as a general base in TfSGL (GH162) and this residue corresponds to a hydrophobic residue in XcSGL and CpSGL (GH144) (Figures 7b,g and 8f), indicating that the positions of the general base catalysts are different between TfSGL and CpSGL (Tanaka et al., 2018, 2019). However, there are several acidic amino acid residues as candidates for the general base catalyst (Abe et al., 2017). The mutation of D135, D139, and E142 in CpSGL drastically decreases the hydrolytic activity, while the mutation of D400 retains sufficient hydrolytic activity (Abe et al., 2017), although D400 is conserved in many of the groups in the SGL clan (Figures 8b,f and S16). D135 is located far from subsite −1, as judged from superimposition of the structure with the XcSGL‐Sop_7_ complex (Figure 7a,b), and is not conserved in the clan (Figure 8b). D139 is also substituted for non‐acidic residues in the other groups in the clan. In contrast, E142 is conserved as an acidic residue in all the enzymes, except for PgSGL4 (Group 4) and TfSGL (GH162) (Figure 8b).

The residues corresponding to E142 in CpSGL are D149 in EeSGL1 (Group 1) and D148 in PgSGL3 (Group 3) (Figure 8b). Both the D149N mutant (EeSGL1) and D148N mutant (PgSGL3) showed drastically decreased activity as described above. Although mutational analysis of D160 in SkSGL is not performed, Sop_7_ is docked in SkSGL in almost the same conformation as EeSGL1 (Figures 7c,d, S13AB, and S16). These results suggested that the residues corresponding to E142 of CpSGL are the most plausible candidates for the general base in GH144 and Groups 1–3.

Recently, GH186 SGL from *Escherichia coli* was found to have a unique anomer‐inverting reaction mechanism mediated by the 4‐OH group at subsite −1 and three water molecules (including a nucleophilic water) (Motouchi et al., 2023) (Figure 9d). GH144 and Groups 1–3 SGLs possess acidic amino acids at the same position as the general base in GH186 SGL from *E. coli* (Figure 9c,d). This observation provides the possibility that GH144 and Groups 1–3 SGLs follow a similar reaction mechanism as GH186 SGL. However, the side chains of hydrophobic residues (I453 and F286 in the case of XcSGL, Figure 7b–e) at the bottom of the Glc unit at subsite −1 are likely to be unfavorable for the access of water molecules. Actually, no water molecule is observed at the corresponding space in the complex structure of XcSGL‐Sop_7_ (Figure 7a), suggesting that a water molecule is not fixed at a specific position. These observations suggest that there could be unknown plausible reaction routes for GH144 and Group 1–3 SGLs. Similarity in structural environments of catalytic pockets in GH144 and Groups 1–3 SGLs also suggests that these groups share the same reaction mechanism. In any case, the acidic residues that are candidates for acid catalysts of GH144 and Groups 1–3 SGLs are located differently from those of TfSGL (GH162) and TiCGS_Tg_ (GH189) (Figures 7c–e,g,h, 8b,e,f, and 9a–c).

### 3.4 Comparison of family classification with the case of other GH families

In this study, we found four groups of sequences with previously unknown functions that were related to GH144, GH162, and GH189. The biochemical functions were determined in three of the four newly identified groups. The three groups with identified functions are believed to share a reaction mechanism with GH144 but not with GH162 or GH189. Multiple sequence alignment revealed that only three residues were mostly conserved in the SGL clan. The amino acid sequence identities in the clan were mostly less than 20%, and Groups 1–4 (Figure S5) were placed irregularly in the phylogenetic tree in terms of the reaction mechanisms (Figure 1).

An example of several GH families forming a clan is found as clan GH‐Q containing the GH94, GH149, and GH161 families of glycoside phosphorylases (Kuhaudomlarp et al., 2018; Kuhaudomlarp, Pergolizzi, et al., 2019; Kuhaudomlarp, Stevenson, et al., 2019). In this superfamily, the catalytic residues, inorganic phosphate recognition residues, and subsite −1 recognition residues are conserved (Kuhaudomlarp, Stevenson, et al., 2019), suggesting that the reaction mechanism is the same for all the members of clan GH‐Q. In addition, the amino acid sequence identities in these three families are 16%–20%. Another recent example of a potential clan is GH109 and GH188 enzymes, NAD^+^‐dependent enzymes with Rossmann folds as catalytic domains (Kaur et al., 2023; Teze et al., 2020). The amino acid sequence identity and RMSD between them (Protein Data Bank [PDB] IDs 6T2B and 8QC2) are 15% and 2.22 Å, respectively. Catalytic histidine residues are conserved between them. Based on amino acid sequence identity and RMSD values, Groups 1 and 3 in the present study should be classified into separate new GH families. The relationship between Group 2 and GH189 should be discussed in structural terms because the amino acid sequence identity between Group 2 and GH189 is somewhat higher than in the examples discussed above.

The overall structures of representative proteins of Group 2 and GH189 share the same fold with high similarity (Figures 5 and 6). GH144, GH162, and Groups 1–3 share the same anomer‐inverting mechanism, and potential general acid residues are conserved among them (Figures 4 and 7, 8, 9). Based on these findings, Groups 1–3 are classified into clan GH‐S. However, GH189 follows the anomer‐retaining mechanism, which is different from the GH families that possess SGLs. Thus, GH189 is excluded from clan GH‐S (personal communication from the Glycogenomics team responsible for the CAZy classification/database). Group 2 is clearly separated from GH189 in terms of family classification.

That GH97 has different reaction mechanisms should be taken into consideration when discussing the classification of GH families. GH97 possesses anomer‐inverting α‐glucosidases and anomer‐retaining α‐galactosidases. BT_3703 is a GH97 α‐glucosidase whose structure complexed with a substrate is available. Among structurally characterized α‐galactosidases of GH97, BT_3661 is the least similar to BT_3703. When comparing the catalytic domains alone, the amino acid sequence identity and RMSD between BT_3703 and BT_3661 are 28% and 1.58 Å, respectively. These values are a little higher and lower, respectively, than those in the case of Group 2 (SkSGL) and GH189 (TiCGS_Tg_) described in the present work.

BT_3703 has residue E532 as a general acid and E439 and E508 as general bases for activation of a nucleophilic water (Kitamura et al., 2008). Although it is unique that two acidic residues participate in interaction with a nucleophilic water, the enzyme is typical in that the residues interact directly with the nucleophilic water. In BT_3661, a potential acid/base catalyst (E463) is well‐superimposed with the general acid in BT_3703. A potential nucleophile in BT3661 is D408 because a corresponding residue, D415, is defined as a nucleophile in BT_1871, a well‐characterized α‐galactosidase (Okuyama et al., 2009). D408 is located at the position where the nucleophilic water in BT_3703 resides when BT_3661 and BT_3703 are superimposed. Although no structure complexed with a substrate is available for a GH97 α‐galactosidase, nucleophilic attack in GH97 α‐galactosidases is a typical reaction mechanism of GH enzymes.

Altogether, the two reaction mechanisms in GH97 are typical retaining and inverting mechanisms. The reaction of SkSGL cannot be via a typical inverting mechanism, although the mechanism has not been fully unveiled. Thus, Group 2 (SkSGL) and GH189 (with a typical anomer‐retaining mechanism in terms of the nucleophile) do not represent a pair of typical retaining and inverting mechanisms. This is clearly different from the case of GH97. Overall, Groups 1–3 with functions unveiled in this study define new GH families, GH192, GH193, and GH194, respectively.

### 3.5 Concluding remarks

The extensive distribution of SGLs found in this study suggests the importance of β‐1,2‐glucans and related enzymes in nature. Phylogenetic groups with the same reaction mechanism in the SGL clan are dispersed irregularly and most substrate recognition residues are substituted between the families. These facts suggest that complicated and unique molecular evolution has occurred in this clan. The identification of this clan showcases the extensive diversity of “CAZymes”.

## 4 MATERIALS AND METHODS

### 4.1 Materials

Glucomannan, polygalacturonic acid, carboxymethyl curdlan, lichenan, arabinogalactan, barley β‐glucan, tamarind xyloglucan, arabinan, and beechwood xylan were purchased from Neogen (MI, USA). Laminarin and carboxymethyl cellulose were purchased from Merck (NJ, USA). Soluble starch and pustulan were purchased from FUJIFILM Wako Pure Chemical Corporation (Osaka, Japan) and CarbioChem (CA, USA), respectively.

β‐1,2‐Glucans with an estimated average DP25 calculated by nuclear magnetic resonance (Nakajima et al., 2014) and average DP121 and DP17.7 calculated from the number average molecular weight (Kobayashi et al., 2019; Motouchi et al., 2023; Nakajima et al., 2021) (β‐1,2‐Glucans [DP25], [DP121], and [DP17.7]) were prepared as reported previously. β‐1,2‐Glucans (DP121) used for the colorimetric assay were treated with NaBH_4_ (β‐1,2‐glucan alditols) to reduce the colorization derived from the anomeric hydroxy groups at the reducing end of the polysaccharide (Kobayashi et al., 2018).

### 4.2 Sequence analysis

The amino acid sequences of TfSGL (GH162) and CpSGL (GH144) were used as queries for a PSI‐BLAST search to collect 1000 and 5000 homologs, respectively. PgSGL1, PgSGL3, SkSGL, and CGSs were included in this collection. Then, a BLASTP search was performed using PgSGL1, PgSGL3, SkSGL (residues 72–541 a.a.), CGS from *B. abortus* (residues 1009–1515 a.a.; GenBank accession number, ACD71661.1), and a homolog from *Phycisphaerae bacterium* (GenBank accession number, MBN1513595.1) with the settings to collect up to 5000 homologs for the SkSGL and CGS groups and up to 1000 homologs for the other groups. Because the CGS group contained too many homologs in the database for the search, several organisms with many species were eliminated as targets for the search. The homolog from *P. bacterium* was used as a query because this homolog is located at the far side from CpSGL according to a phylogenetic tree analysis of GH144 (Abe et al., 2017). Homologs were collected until no new group was obtained. During this collection, the PgSGL4 group, a small group, was found. The number of homolog hits found initially by BLASTP using a query in each group was approximately 5000 for GH144 CpSGL, 4100 for GH144 (MBN1513595.1), 430 for GH162 TfSGL, 1000 for SkSGL, 330 for the SkSGL group (MBN1384282.1), 480 for PgSGL3, 140 for PgSGL1, and 4200 for CGS. Because the GH144 and CGS groups are large, the GH144 proteins and GT84 family proteins (intact CGSs) in the CAZy database (Drula et al., 2022; Levasseur et al., 2013) were also collected (1604 and 1930 homologs, respectively). Then, the duplicated sequences were removed from all the collected sequences. Multiple sequence alignment was performed against each group using Clustal Omega to extract homologs sparsely from each group (Larkin et al., 2007). The transglycosylation domain and catalytic domain were extracted for GH189 and Group 4, respectively. Then, homologs showing less than 65%–95% amino acid sequence identity with the other homologs in their groups were extracted. The cut‐off values were 95%, 95%, 66%, 65%, 55%, 95%, and 68% for the groups of PgSGL1, PgSGL3, SkSGL, SkSGL group (MBN1384282.1), CGS, GH162, GH144, and the homolog from *P. bacterium*, respectively. The latter two groups were combined into the GH144 group. Because the newly found PgSGL4 group was small, all the homologs in this group were used for preliminary phylogenetic analysis. Eleven homologs in the PgSGL3 group were transferred to the PgSGL4 group, and one homolog in the SkSGL group was transferred to the PgSGL1 group based on the phylogenetic positions. Finally, 20–220 homologs were obtained from each group (PgSGL1, 42; PgSGL3, 148; SkSGL, 186; PgSGL4, 20; CGS, 144; GH144, 212; and GH162, 168). All the extracted homologs containing the samples used as queries were aligned by multiple alignment using MUSCLE (Edgar, 2004) and a preliminary phylogenetic tree was prepared based on the alignment by MEGA11 (Jones et al., 1992; Tamura et al., 2021) (Data S1 and S2). To simplify the tree, 5–12 homologs, including the samples used in the present study, were selected sparsely in each family or group. Then, the tree was constructed based on the maximum‐likelihood method using 100 bootstraps, which was visualized by MEGA11 (Jones et al., 1992; Tamura et al., 2021) (see Data S3 and S4 for the original data). To investigate conservation of the substrate recognition and catalytic residues in the SGL clan, structure‐based multiple sequence alignment was performed using the SALIGN server (https://modbase.compbio.ucsf.edu/salign/) (Braberg et al., 2012). This alignment was visualized using the ESPript 3.0 server (http://espript.ibcp.fr/ESPript/ESPript/) (Robert & Gouet, 2014).

### 4.3 Cloning, expression, and purification

The genomic DNA of *P. gaetbulicola* (DSM 26887), *E. elysicola* (DSM 22380), and *X. campestris* pv. *campestris* (DSM 3586) was purchased from the Leibniz Institute DSMZ (Leibniz, Germany) and *S. keddieii* ST‐74 (ATCC 51767) was purchased from the American Type Culture Collection (VT, USA). Genes coding PgSGL1 (Kyoto Encyclopedia of Genes and Genomes (KEGG) locus tag, H744_1c0224), PgSGL2 (KEGG locus tag, H744_2c01936), PgSGL3 (KEGG locus tag, H744_1c0222), PgSGL4 (KEGG locus tag, H744_1c0194), EeSGL1 (National Center for Biotechnology Information accession number, WP_026258326.1), SkSGL (KEGG locus tag, Sked_30460), and XcSGL (KEGG locus tag, XCC2207) were amplified by polymerase chain reaction (PCR) using KOD plus (TOYOBO, Osaka, Japan) or PrimeSTAR Max (Takara Bio, Shiga, Japan) for the DNA polymerase and the primer pairs. All the primers used in this study were designed not to include N‐terminal signal peptides, as shown in Table S4. The N‐terminal signal peptides were predicted using SignlaP5.0 or SignalP4.1 (Almagro Armenteros et al., 2019; Petersen et al., 2011). Each second methionine residue (in the database for XcSGL or based on the sequenced nucleotide sequence for SkSGL) was used as a starting residue for searching a signal peptide. Residue numbers are counted from the starting residues in XcSGL and SkSGL throughout the manuscript. The amplified PCR products were digested by the restriction enzymes shown in Table S4. Vectors (pCold I and pET30a) were also digested by the corresponding restriction enzymes suitable for ligation. The digested PCR products of PgSGL1, PgSGL2, and EeSGL1 were inserted into pCold I (Takara, Shiga, Japan) to fuse a His_6_‐tag at the N‐terminus. The digested PCR products of PgSGL3 and XcSGL, and SkSGLc were inserted into pET30a and pET24a (Merck), respectively, to fuse a His_6_‐tag at the C‐terminus. Ligation high ver. 2 (TOYOBO, Osaka, Japan) was used for the insertion.

Mutations used in this study were introduced using PrimeSTAR Max according to the manufacturer's instructions using the primer pairs shown in Table S4. The region coding the putative N‐terminal signal peptide of SkSGL was removed using PrimeSTAR Max and the primer pair (SkSGL Δsignal Fw and Rv). To transfer the C‐terminal His_6_‐tag of SkSGLc to the N‐terminus, the gene coding SkSGLc and the pCold I vector were amplified using KOD One (TOYOBO) and the primer pairs (SkSGL pCold Fw and Rv, and pCold I Fw and Rv, respectively). The former amplified PCR product was inserted into pCold I (the latter amplified PCR product) by recombination using the SLiCE method (Motohashi, 2015). The amplified PCR product of the gene coding PgSGL4 was inserted into pCold I by the same method as for SkSGLn. The resulting products were transferred into JM109 or XL1‐blue, and then the plasmids were extracted, and the target DNA sequences were checked as described above. This SkSGL fused with a His_6_‐tag at the N‐terminus was called SkSGLn.

The constructed plasmids were transferred into *E. coli* BL21(DE3). The recombinant BL21(DE3) were cultured in Luria‐Bertani medium to produce target proteins. The strains with the target gene inserted into pET30a were cultured in medium containing kanamycin (30 μg/mL) at 37°C with vigorous agitation (120 rpm) until the OD_600_ value exceeded approximately 0.8 and then isopropyl β‐d‐1‐thiogalactopyranoside (IPTG) was added to a final concentration of 0.1 mM. The cells were further cultured at 20°C overnight. The strains with the target gene inserted into pCold I were cultured in medium containing ampicillin (100 μg/mL) at 37°C with vigorous agitation (120 rpm) until the OD_600_ value exceeded approximately 0.6. Then, the cells were left at 10–15°C for 30 min. IPTG was added to a final concentration of 0.1 mM and the cells were agitated again at 10–15°C overnight. After the cells were collected by centrifugation at 6000 × *g* for >5 min, the cells were suspended in an appropriate volume of Tris–HCl (pH 7.5) for SkSGLc and SkSGLn or 30–50 mM 3‐morpholinopropanesulfonic acid (MOPS) (pH 7.0–7.5) for the other proteins. The suspended cells were disrupted by sonication and the supernatants were collected by centrifugation at 33000 × *g* for >10 min. The supernatants, except for SkSGLc, were purified by the HisTrap™ FF crude column (Cytiva). Each sample was loaded onto a column equilibrated with an equilibration buffer composed of 20–50 mM MOPS (pH 7.5) (EeSGL1 and PgSGL3) or Tris–HCl (pH 7.5) (SkSGLn, XcSGL, PgSGL1 and PgSGL2) and 200–500 mM NaCl. The column was washed with the equilibration buffer containing 10–15 mM imidazole until almost all the unbound compounds were eluted. The samples were eluted with 50 mL of a linear gradient of up to 500 mM imidazole. For SkSGLc, other chromatography methods were used because the protein did not bind to a HisTrap™ FF crude column (5 mL; Cytiva, MA, USA). The cells were suspended with 50 mM Tris–HCl (pH 7.5). The supernatant was loaded onto tandemly connected HiTrap™ DEAE FF (5 mL; Cytiva) columns equilibrated with 20 mM Tris–HCl (pH 7.5). After the unbound compounds were almost all eluted with the buffer, the target protein was eluted with 100 mL of the buffer with a linear gradient of 0–1 M NaCl at a flow rate of 3 mL/min. The fractions containing the target protein were collected and mixed with an equivalent volume of 20 mM Tris–HCl (pH 7.5) containing 60% saturation ammonium sulfate. After the solution was centrifuged at 33000 × *g* for 20 min, the supernatant was loaded onto a HiTrap™ Butyl HP column (5 mL, Cytiva) equilibrated with 20 mM Tris–HCl (pH 7.5) containing 30% saturation ammonium sulfate. The samples were eluted with a linear gradient of 30%–0% saturation ammonium sulfate. The fractions containing the target protein were collected. The activity in the fractionated samples was detected by TLC.

For crystallization of the XcSGL E239Q mutant (a catalytic residue mutant), the enzyme was purified by nickel affinity chromatography and further purified by hydrophobic interaction chromatography. The fractionated eluates from the HisTrap™ FF column were mixed with an equivalent volume of 50 mM MOPS (pH 7.0) containing 50% saturation ammonium sulfate. The sample was loaded onto a HiTrap™ Butyl column equilibrated with 50 mM MOPS (pH 7.0) containing 25% saturation ammonium sulfate. The sample was eluted with a linear gradient of 25%–0% saturation ammonium sulfate. The fractions containing the target protein were pooled and dialyzed against 5 mM MOPS (pH 7.0). For PgSGL3 and EeSGL1, the extra purification steps were not used.

The homogeneity of all the target proteins was checked using sodium dodecyl sulfate‐polyacrylamide gel electrophoresis (Figure S17). DynaMarker Protein MultiColorIII (BioDynamics Laboratory Inc., Japan) was used for protein standards. Each target protein was buffered and concentrated in 5 mM MOPS (pH 7.0) by ultrafiltration with AmiconUltra (Millipore, MA, USA) or VivaSpin (Sartorius, Germany) with molecular weight cut‐off values of 10,000 or 30,000 at less than 4000 × *g*. For PgSGL2, a buffer containing 5 mM MOPS (pH 7.0) and 150 mM NaCl was used. The protein concentrations were calculated using the theoretical molecular masses and the extinction coefficients at an absorbance of 280 nm according to Pace et al. (1995) and are summarized in Table S5.

### 4.4 Temperature and pH profiles

To investigate the optimum pH for each enzyme, 0.2% β‐1,2‐glucan alditols was incubated with an appropriate concentration of each enzyme in 50 mM buffer (containing 500 mM NaCl in the case of PgSGL2) at 30°C. To investigate the optimal temperature, 0.2% β‐1,2‐glucan alditols was incubated with an appropriate concentration of each enzyme in the assay buffer (50 mM bicine pH 8.5 for PgSGL1; 50 mM 2‐morpholinoethanesulfonic acid (MES) [pH 6.5] containing 500 mM NaCl for PgSGL2; 50 mM MOPS pH 7.0 for EeSGL1; 50 mM cacodylate pH 6.5 for PgSGL3; 50 mM HEPES, pH 8.0 for SkSGLc; and 50 mM sodium acetate, pH 5.0 for XcSGL). To examine the pH stability, PgSGL1 (10.9 μg/mL), PgSGL2 (0.284 mg/mL), EeSGL1 (4 μg/mL), PgSGL3 (8 μg/mL), and SkSGL (5.2 μg/mL) were incubated with 50 mM buffer solutions (5 mM for EeSGL1 and PgSGL3) at 30°C for 1 h. Then, the enzymatic reaction was performed in the presence of 0.2% β‐1,2‐glucan alditols, an appropriate concentration of each enzyme, and the corresponding assay buffer. To examine the temperature stability, PgSGL1 (0.0109 mg/mL), PgSGL2 (0.284 mg/mL), EeSGL1 (4 μg/mL), PgSGL3 (8 μg/mL), and SkSGL (5.2 μg/mL) were incubated in the corresponding assay buffer (except that 5 mM MOPS [pH 7.0] containing 500 mM NaCl was used for PgSGL2) at various temperatures for 1 h. Then, each enzyme diluted to an appropriate concentration was incubated in the substrate solution containing 0.2% β‐1,2‐glucan alditols and the corresponding assay buffer at 30°C. The incubation times for the enzymatic reactions were 10 min for PgSGL2, PgSGL3, and EeSGL1; 20 min for PgSGL1; and 30 min for SkSGL and XcSGL. The reaction products were colorized by the 3‐methyl‐2‐benzo‐thiazolinone hydrazone (MBTH) method described below. The assays were performed in triplicate.

### 4.5 Size‐exclusion chromatography

Superdex™ 200 (HiLoad 16/60, Cytiva) was equilibrated with 50 mM Tris–HCl (pH 8.0) containing 0.15 M NaCl, and each sample (0.8 mg of EeSGL1, 500 μL; 0.2 mg of PgSGL3, 500 μL) or a marker mixture (500 μL) was loaded onto the column at a flow rate of 0.5 mL/min. Ovalbumin (43 kDa), conalbumin (75 kDa), aldolase (158 kDa), ferritin (440 kDa), and thyroglobulin (669 kDa) were used as protein markers. Blue dextran 2000 was also added to the protein markers to determine the void volume of the column. The amounts of ferritin and aldolase were 0.1 and 1.5 mg, respectively, and the amounts of the other marker proteins and blue dextran 2000 were 0.5 mg. For SkSGLc, 50 mM Tris–HCl (pH 8.0) was replaced with 20 mM Tris–HCl (pH 7.5). Elution of the samples was performed at a flow rate of 0.3 mg/mL. The molecular weights of EeSGL1, PgSGL3, and SkSGLc were calculated using Equation (1).

(1) $$K_{\mathrm{av}}=\frac{V_{e}-V_{o}}{V_{t}-V_{o}},$$

where *K*_av_ is the gel‐phase distribution coefficient; *V*_e_ is the volume required to elute each protein; *V*_o_ is the volume required to elute blue dextran 2000; and *V*_t_ is the bed volume of the column.

### 4.6 Substrate specificity and kinetic analysis

Enzymatic reactions were performed in the assay buffer containing each substrate at 30°C for the times described above. The assays were performed in triplicate using the assay buffers described in the Temperature and pH profiles section. The relative activity when 0.2% β‐1,2‐glucan alditols was used as the substrate was determined for each candidate catalytic residue mutant. For kinetic analysis, the median values were plotted and fitted with a Michaelis–Menten equation (Equation 2) using R.

(2) $$\;\frac{v}{{\left[E\right]}_{T}}=\frac{k_{\mathrm{cat}}\left[S\right]}{K_{m}+\left[S\right]},$$

where *v* is the initial velocity; [*E*] is the enzyme concentration; *k*_cat_ is the turnover number; [*S*] is the β‐1,2‐glucan concentration; and *K*_m_ is the Michaelis constant.

### 4.7 MBTH method

The reducing power in the reaction mixtures was determined using the MBTH method (Kobayashi et al., 2018). The reaction solution (20 μL), a solution containing 1 mg/mL dithiothreitol and 3.0 mg/mL MBTH (20 μL) and 0.5 M NaOH (20 μL) were mixed, and then the mixture was heated at 80°C for 30 min. After the mixture was cooled to room temperature, 40 μL of a solution containing 0.5% FeNH_4_(SO_4_)_2_ · 2H_2_O, 0.5% sulfamic acid, and 0.25 M HCl, and 100 μL water was added. The mixtures (175 μL) were poured into a 96‐well microplate (EIA/RIA plate, 96‐well half area, Corning, NY, USA) and then the absorbance at 620 nm was measured. Sop_2_ was used as a standard for assaying the activity toward β‐1,2‐glucan alditols, and Glc was used instead of the other substrates.

### 4.8 TLC analysis

The components of the reaction mixtures of EeSGL1, PgSGL3, and SkSGLc were 50 mM MOPS (pH 7.0), 0.2% β‐1,2‐glucan (DP121), and 4 μg/mL of EeSGL1; 20 mM MOPS (pH 7.0), 0.2% β‐1,2‐glucan (DP121) or Sop_8_, and 0.025–1 mg/mL PgSGL3; and 20 mM MOPS (pH 7.5), 0.2% β‐1,2‐glucan alditols, and 8.6 μg/mL of SkSGLc, respectively. The reactions were performed at 30°C for 20 min (PgSGL3) or an appropriate time as shown in Figure 2 (EeSGL1 and SkSGLc). The reaction was stopped by heating the sample at 80°C for PgSGL3 or 100°C for the other enzymes, for 5 min. The reaction mixtures and markers were spotted onto TLC Silica Gel 60 F_254_ (Merck) plates. After the plates were developed with 75% acetonitrile twice or more, the plates were soaked in a 5% (w/v) sulfuric acid/methanol solution. Spots were visualized by heating the plates in an oven. The Sop_*n*_s marker used for lane M1 of PgSGL3 was prepared by incubating a 1% mixture of Sop_2–5_, which are the reaction products of CpSGL, with SOGP from *L. innocua* in the presence of 1 mM sodium phosphate (Motouchi et al., 2023). The Sop_*n*_s marker used for SkSGLc was prepared by incubating a 0.5% mixture of Sop_3–7_, which are the reaction products by SGL from *Chloroflexus aurantiacus* (Chy400_4164), with SOGP from *Enterococcus italicus* in the presence of 25 mM sodium phosphate (Kobayashi et al., 2019; Nakajima et al., 2021).

### 4.9 Polarimetric analysis of the reaction products

To determine the reaction mechanisms of PgSGL1, PgSGL3, and SkSGL, the time course of the degree of optical rotation in the reaction mixture was monitored. Approximately 10 mL of a substrate solution containing 2% β‐1,2‐glucan (DP25) (Abe et al., 2017; Tanaka et al., 2019) and 50 mM bicine (pH 8.5) was first warmed and then poured into a cylindrical glass cell (100 mm × ø 10.5 mm). The sample was placed in a JASCO P‐2200 polarimeter (Jasco, Japan) until the monitored value stabilized. Then, the reaction was started by adding PgSGL1 solution (200 μL, 40.4 mg/mL) at room temperature. When the reaction velocity began to slow down, droplets of 35% aqueous ammonia were added to enhance the mutarotation of the anomers. For PgSGL3, 2.5 mM MOPS (pH 6.5) buffer was used for the substrate solution, and 300 μL of 9.6 mg/mL PgSGL3 was added. For SkSGL, 1.6 mL of substrate solution containing 1% β‐1,2‐glucan (DP25) and 21 mM HEPES (pH 8.0) was poured into a cylindrical glass cell (100 mm × ø 3.5 mm) because it was difficult to obtain a large amount of SkSGLn or SkSGLc, and the hydrolytic activity of SkSGL decreased in the presence of a high substrate concentration. SkSGLn solution (400 μL, 0.91 mg/mL) was added to the substrate solution. The principle of the assay is shown in Figure 4d.

### 4.10 Crystallography

The initial screening of the crystallization conditions for the XcSGL E239Q mutant, PgSGL3, and EeSGL1 was performed using JCSG‐plus™ HT‐96 and PACT premier™ HT‐96 (Molecular Dimensions, UK). After 1 μL of protein solution (10 mg/mL) was mixed with 1 μL of reservoir solution on a sitting plate (96‐well CrystalQuick plates, Greiner Bio‐One, Germany), the plate was incubated at 20°C. The crystallization conditions were optimized from the results of the initial screening by the hanging drop vapor diffusion method using VDX plates (Hampton Research, CA, USA). Finally, crystals of the XcSGL E239Q mutant were prepared by incubation of a mixture of 11.9 mg/mL of the mutant (1 μL) and 1 μL of reservoir solution composed of 20 mM NaNO_3_, 4.5% (w/v) polyethylene glycol (PEG) 3350, and 0.1M bis‐tris propane (pH 5.5) at 20°C. For EeSGL1, the crystals obtained from the initial screening were used for data collection. The reservoir component was 0.2 M potassium thiocyanate, 0.1 M bis‐tris propane (pH 6.5), and 20% (w/v) PEG 3350. For PgSGL3, a microseeding method was adopted because the crystallization was difficult to reproduce. Reservoir solution (40 mM HEPES pH 7.0, 80 mM MgCl_2_, and 10% PEG 3350) and 11.7 mg/mL of PgSGL3 (both 1 μL) were mixed and then an appropriate amount of seed crystals created by crushing a PgSGL3 crystal were added to the drop. After incubation of this hanging drop at 20°C for 10 min, crystals were formed of suitable size for data collection.

Each crystal was soaked in the reservoir solution supplemented with 12% (w/v) PEG 200, 8% glycerol, and 4% β‐1,2‐glucan (DP17.7) for the XcSGL E239Q mutant, 25% PEG 400 for EeSGL1, and 25% (w/v) PEG 400 for PgSGL3 as cryoprotectants and maintained at 100 K in a nitrogen‐gas stream during data collection. All x‐ray diffraction data were collected on beamlines (BL‐5A and NW‐12A) at Photon Factory (Tsukuba, Japan). The data reduction of the diffraction data was performed using the XDS program (Kabsch, 2010). The initial phase information was obtained by molecular replacement using the MOLREP program (Vagin & Teplyakov, 2010). The structures of XcSGL and PgSGL1 predicted by AlphaFold2 (Jumper et al., 2021) were obtained from the AlphaFold Protein Structure Database (https://alphafold.ebi.ac.uk/) (Varadi et al., 2022) (ID, AF‐Q8P8N3‐F1 and AF‐A0A0C5WQQ2‐F1, respectively) as model structures to solve the XcSGL mutant and EeSGL1 structures, respectively. For PgSGL3, the iodide single‐wavelength anomalous diffraction phasing method using the diffraction data for the iodinated PgSGL3 crystal collected at 1.8 Å was adopted (Miyatake et al., 2006). The program used was the Crank2 program (shelxc, shelxd, refmac, solomon, multicomb, buccaneer, and parrot) in ccp4 (http://www.ccp4.ac.uk/) (Agirre et al., 2023). Model building for EeSGL1 was performed using Buccaneer (Cowtan, 2006). Refinement of the structures was performed using Refmac5 (Murshudov et al., 1997) for automatic refinement and Coot for manual refinement (Emsley & Cowtan, 2004). In the case of XcSGL, the final space group was determined after trial with various space groups because *R*_work_ and *R*_free_ values were high. The reason why these values are high is described in Note 3, Supporting Information S5 (Figure S18).

### 4.11 MD simulations

For the MD simulations, Sop_8_ was placed in the substrate‐binding pockets of EeSGL1, SkSGL, and PgSGL3, referring to the complex structure of TfSGL (PDB ID, 6IMW). The crystal structures of EeSGL1 and PgSGL3, and the predicted structure of SkSGL were used. To reduce calculation costs, only chain A of the EeSGL1 dimer and a catalytic domain of SkSGL (64–545 a.a.) were used for MD. The proteins and Sop_8_ were protonated and placed in a dodecahedral box. The box size was determined so that all molecules were placed at least 1.5 nm from the box edges. The periodic boundary conditions were applied in all directions. The box was filled with water molecules. Sodium and chloride ions were added to each box to neutralize the total charge as well as to set the ion density to 10 mM. The AMBER ff14SB force field (Maier et al., 2015) and GLYCAM (Kirschner et al., 2008) were used to represent proteins and Sop_8_, respectively. The TIP3P model (Jorgensen et al., 1983) was used for water. After energy minimization, the constant‐pressure and constant‐temperature (NPT) MD simulations for equilibration were performed at 1 bar and 300 K for 200 ps. Position restraints were applied to the Cα atoms of the proteins and all heavy atoms of Sop_8_ during equilibration. The production runs were performed for 1 μs at 300 K. The C‐rescale method and Parrinello‐Rhaman method were used to maintain the pressure during the equilibrations and the production runs, respectively. The covalent bonds of hydrogen atoms were constrained using the LINear Constraint Solver method, and the integration time step was 2.0 fs. MD simulations were performed using GROMACS 2022.4 (Abraham et al., 2015).

### 4.12 Accession numbers

The atomic coordinates and structure factors (codes 8XUJ, 8XUK, and 8XUL) have been deposited in the PDB.

## AUTHOR CONTRIBUTIONS

**Masahiro Nakajima:** Conceptualization; funding acquisition; writing – review and editing; writing – original draft; investigation. **Nobukiyo Tanaka:** Investigation; writing – review and editing. **Sei Motouchi:** Investigation; writing – review and editing. **Kaito Kobayashi:** Investigation; writing – review and editing. **Hisaka Shimizu:** Investigation; writing – review and editing. **Koichi Abe:** Investigation; writing – review and editing. **Naoya Hosoyamada:** Investigation; writing – review and editing. **Naoya Abara:** Investigation; writing – review and editing. **Naoko Morimoto:** Investigation; writing – review and editing. **Narumi Hiramoto:** Investigation; writing – review and editing. **Ryosuke Nakata:** Investigation; writing – review and editing. **Akira Takashima:** Investigation; writing – review and editing. **Marie Hosoki:** Investigation; writing – review and editing. **Soichiro Suzuki:** Investigation; writing – review and editing. **Kako Shikano:** Investigation; writing – review and editing. **Takahiro Fujimaru:** Investigation; writing – review and editing. **Shiho Imagawa:** Investigation; writing – review and editing. **Yukiya Kawadai:** Investigation; writing – review and editing. **Ziyu Wang:** Investigation; writing – review and editing. **Yoshinao Kitano:** Investigation; writing – review and editing. **Takanori Nihira:** Investigation; writing – review and editing. **Hiroyuki Nakai:** Conceptualization; writing – review and editing. **Hayao Taguchi:** Investigation; writing – review and editing.

## CONFLICT OF INTEREST STATEMENT

The authors declare no competing interests.

## Supporting information


**Data S1.** Supporting Information.

**Data S2.** Supporting Information.

**Data S3.** Supporting Information.

**Data S4.** Supporting Information.

**Data S5.** Supporting Information.

## Data Availability

The data that support the findings of this study are available from the corresponding author upon reasonable request.
