# Supplementary material for: New glycoside hydrolase families of β‐1,2‐glucanases
Source: Protein Sci. 2025 May 24;34(6):e70147. doi: 10.1002/pro.70147 (PMC12102758; doi:10.1002/pro.70147)
Supplement: Supplementary file 1 — Data S1. Supporting Information. [file PRO-34-e70147-s003.docx]

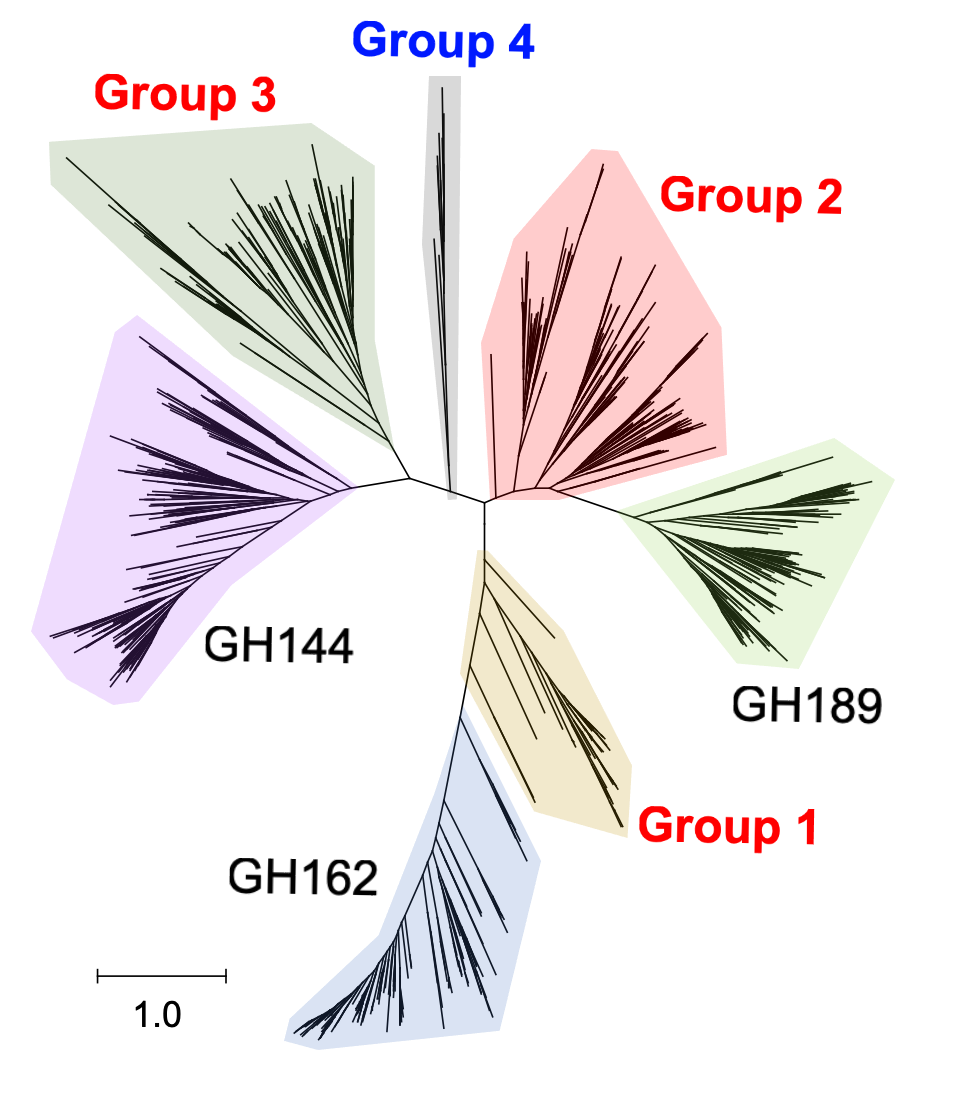


**Supplemental Data. A preliminary phylogenetic tree of the SGL clan without application of a bootstrap test**

Up to 250 proteins were collected from each group to create the phylogenetic tree using the method described in the Sequence Analysis section. Biochemically identified groups are labelled in bold red letters (Groups 1–3) and the unidentified group is in bold black letters (Group 4). Groups that have already been defined are labelled with the family numbers (GH144, GH162, and GH189). The groups are colored light purple (GH144), light blue (GH162), light green (GH189), light yellow (Group 1), light red (Group 2), dark green (Group 3), and light gray (Group 4). A scale bar is provided in the bottom left corner to show the phylogenetic distances.
