# Supplementary figures and images for: New glycoside hydrolase families of β‐1,2‐glucanases
Source: Protein Sci. 2025 May 24;34(6):e70147. doi: 10.1002/pro.70147 (PMC12102758; doi:10.1002/pro.70147)

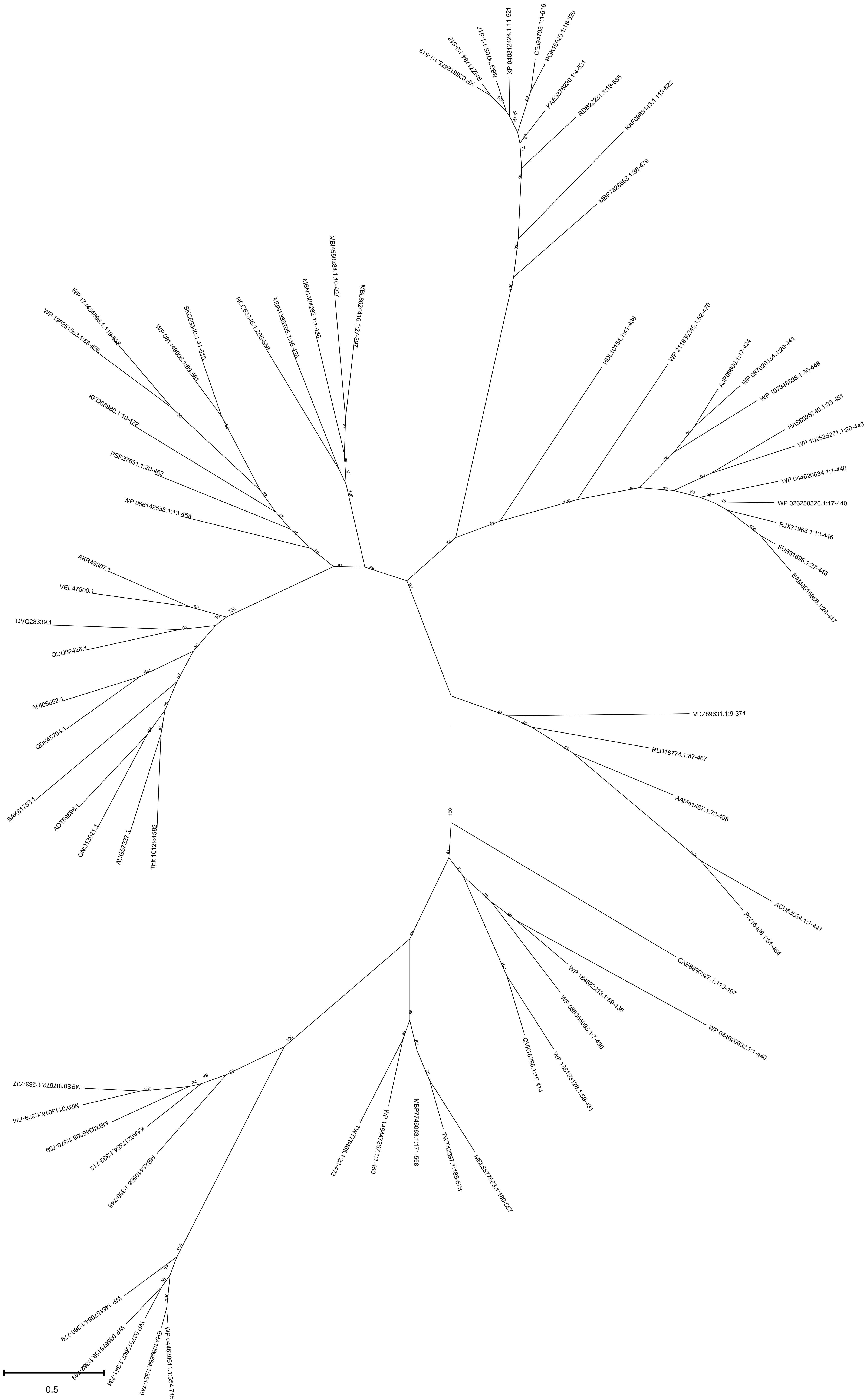

Supplement: Supplementary file 3 — Data S3. Supporting Information. [file PRO-34-e70147-s001.pdf]
