# Supplementary material for: New glycoside hydrolase families of β‐1,2‐glucanases
Source: Protein Sci. 2025 May 24;34(6):e70147. doi: 10.1002/pro.70147 (PMC12102758; doi:10.1002/pro.70147)
Supplement: Supplementary file 5 — Data S5. Supporting Information. [file PRO-34-e70147-s002.docx]

SUPPORTING INFORMATION

**New glycoside hydrolase families of β-1,2-glucanases**

Authors

Masahiro Nakajima*, Nobukiyo Tanaka, Sei Motouchi, Kaito Kobayashi, Hisaka Shimizu, Koichi Abe, Naoya Hosoyamada, Naoya Abara, Naoko Morimoto, Narumi Hiramoto, Ryosuke Nakata, Akira Takashima, Marie Hosoki, Soichiro Suzuki, Kako Shikano, Takahiro Fujimaru, Shiho Imagawa, Yukiya Kawadai, Ziyu Wang, Yoshinao Kitano, Takanori Nihira, Hiroyuki Nakai, Hayao Taguchi

* To whom correspondence should be addressed: Department of Applied Biological Science, Faculty of Science and Technology, Tokyo University of Science, 2641 Yamazaki, Noda, Chiba 278-8510, Japan; [m-nakajima@rs.tus.ac.jp](mailto:m-nakajima@rs.tus.ac.jp)

Contents:

**Supplementary Note 1**: Abbreviation of β-1,2-glucanase and β-1,2-glucooligosaccharide

**Supplementary Note 2**: Targets for analysis in the SGL clan

**Supplementary Note 3**: *R*_work_ and *R*_free_ values of the complex structure of XcSGL

**Supplementary Table S1**: Classification of putative sulfatases in *P. gaetbulicola.*

**Supplementary Table S2**: Data collection and statistics.

**Supplementary Table S3**: Residues defining the SGL clan

**Supplementary Table S4**: Primers used in the study.

**Supplementary Table S5**: Theoretical molecular masses and extinction coefficients of the recombinant proteins.

**Supplementary Figure S1**: Matrices of SGL clan

**Supplementary Figure S2**: A gene cluster of genes encoding PgSGL1, PgSGL3, and PgSGL4 from *P. gaetbulicola*

**Supplementary Figure S3**: pH and temperature profiles of PgSGL1 (Group 1)

**Supplementary Figure S4**: pH and temperature profiles of PgSGL2 (Group 1)

**Supplementary Figure S5**: pH and temperature profiles of EeSGL1 (Group 1)

**Supplementary Figure S6**: pH and temperature profiles of SkSGL (Group 2)

**Supplementary Figure S7**: pH and temperature profiles of PgSGL3 (Group 3)

**Supplementary Figure S8**: Optimal pH and temperature of XcSGL (GH144)

**Supplementary Figure S9**: Effect of NaCl on the hydrolytic activity of PgSGL2

**Supplementary Figure S10**: Size-exclusion chromatography analysis

**Supplementary Figure S11**: Superimposition of overall structures between Groups 1–4 and the known families, GH144, GH162 and GH189

**Supplementary Figure S12**: Comparison of substrate pockets between XcSGL-Sop_7_ and TfSGL-Sop_7_ complexes.

**Supplementary Figure S13**: Structures of the complexes obtained by MD simulations

**Supplementary Figure S14**: Substrate recognition by the catalytic residues in MD simulation

**Supplementary Figure S15**: Surface models of the SGL-clan proteins

**Supplementary Figure S16**: The entire multiple sequence alignment of the SGL-clan proteins

**Supplementary Figure S17**: Purification of SGL-clan enzymes

**Supplementary Figure S18**: Crystal structure of XcSGL-Sop_7_ complex

**References**

**Supplementary Note**

*1. Abbreviation of β-1,2-glucanase and β-1,2-glucooligosaccharide*

“SGL” and “Sop” are used for an abbreviation of β-1,2-glucanase and β-1,2-glucooligosaccharide, respectively, because information of linkage position can be included in abbreviations. “S” and “Sop” are derived from “sophorooligosaccharide”, an alternative name of β-1,2-glucooligosaccharide. They have been used since 1,2-β-oligoglucan phosphorylases was abbreviated as SOGP (Nakajima et al., 2014). Laminari-, cello- and gentio-oligosaccharides are β-1,3-, β-1,4- and β-1,6-glucooligosaccharides, respectively. They are often abbreviated as L, Lam; C, Cel; G, Gen, respectively. Thus, use of “SGL” and “Sop” clearly distinguish linkage positions from the other β-linked glucans.

*2. Targets for analysis in the SGL clan*

Gene clusters for potential β-1,2-glucan-associated enzymes, such as SOGPs and BGLs that prefer Sop_n_s as substrates, were found in the genes encoding the proteins in the SGL clan. Intriguingly, *Photobacterium gaetbulicola*, a bacterium isolated from mudflats (Kim et al., 2010), possesses homologs from multiple groups in the clan: PgSGL1 and PgSGL2 from Group 1; PgSGL3 from Group 3; and PgSGL4 from Group 4 (KEGG locus tags; H744_1c0224, H744_2c1936, H744_1c0222, and H744_1c0194, respectively) (Fig. S3).

The PgSGL4 gene forms a gene cluster with genes encoding a GH94 homolog (H744_1c0196, hereafter, H744_ is omitted), an ABC transporter homolog (1c0198–0200), GH3 homologs (1c0192, 1c0202, and 1c0206), a GH43 (subfamily 28) homolog (1c0207), and a Lac I family protein homolog (1c0197). Although there have been no reports on the characteristics of GH94 homologs in the 1c0196 subgroup, the structures and functions of SOGPs from *Listeria innocua* and *Lachnochlostridium phytofermentans* have been reported (Nakajima et al., 2014, 2017). SOGPs are monomeric enzymes that consist of three major domains, while 1c0196 lacks an *N*-terminal domain and is assumed to form a dimer in a similar manner to other GH94 enzymes, such as cellobiose phosphorylases and chitobiose phosphorylases (Hidaka et al., 2004; Van Hoorebeke et al., 2010).

1c0192 and 1c0202 are close homologs of GH3 BGLs from *L. innocua* and *Bacteroides thetaiotaomicron* that prefer Sop_n_s as substrates (Ishiguro et al., 2017; Nakajima et al., 2016), which suggested that these GH3 homologs from *P. gaetbulicola* were Sop_n_s-preferring BGLs. The structure of 1c0205 was the closest to the GH3 BGL from *Kluyveromyces marxianus* that has a wide substrate preference, of all the structurally available homologs (Yoshida et al., 2010).

GH43 is a large family mainly containing β-xylosidases and α-l-arabinofuranosidases, which is divided into 39 subfamilies (Flipphi et al., 1993; Mewis et al., 2016; Shallom et al., 2005). A homolog in subfamily 28 from a metagenome has been reported to show hydrolytic activity toward arabinoxylooligosaccharides (Maurício da Fonseca et al., 2020). There have been no reports on the biochemical properties of close homologs of the solute-binding subunit (1c0198) in the ABC transporter. 1c0193 and 1c0203, which are sulfatase homologs, belong to the S1 group (Table S1), suggesting that these proteins are involved in the metabolism of sulfated sugars.

The PgSGL1 and PgSGL3 genes are located near the PgSGL4 gene in the *P. gaetbulicola* genome. These genes formed a gene cluster with several putative sulfatase genes (Fig. S3), although PgSGL2 gene is located independently in the genome. According to SulfAtlas (https://sulfatlas.sb-roscoff.fr/sulfatlas/index.html) (Barbeyron et al., 2016; Stam et al., 2023), a database of sulfatases, 1c0221, 1c0223, 1c0227, and 1c0228 belong to the S1 group (Table S1). Enzymes in the S1 group are believed to be involved in the metabolism of sulfated carbohydrates to acidic sugars by acting on the sulfate groups (Bond et al., 1997; Silchenko et al., 2018). 1c0214, 1c0216, and 1c0217 also belong to the S1 group, implying that the gene cluster 1c0206–1c0219 is also involved in the metabolism of sulfated carbohydrates. However, there was no gene encoding a sulfoglycosidase homolog (GH20, GH31, GH185, or GH188) in this region (Bains et al., 2023; Kaur et al., 2023; Liu et al., 2021; Zhang et al., 2023). Overall, PgSGLs 1–4 are expected to be enzymes involved in β-1,2-glucan metabolism. Considering the large number of sulfatase homologs in the region (1c0192–1c0228), sulfated β-1,2-glucans might exist in nature, although to the best of our knowledge, there have been no reported examples.

*Endozoicomonas elysicola*, a bacterium isolated from the gastrointestinal tract of a mollusk, the sea slug *Elysia ornate*, possesses a PgSGL1 homolog (NCBI accession number, WP_026258326.1; EeSGL1) and the structure of EeSGL1 has been analyzed (Kurahashi & Yokota, 2007; Neave et al., 2017). *E. elysicola* possesses the same gene cluster, including PgSGL3 and PgSGL4 homologs, as that of *P. gaetbulicola*.

In Group 2, the function of SGR_2427 from *Streptomyces griseus* can be speculated based on the components of the gene cluster of the *SGR_2427* gene. This gene cluster includes the gene encoding SGR_2426, a putative BGL belonging to GH1, and a solute-binding protein in an ABC transporter homologous with the SO-BP from *L. innocua*. Such gene configurations are found in various genomes, including those of *Microbacterium testaceum* StLB037 and *Nakamurella multipartita* (DSM 44233). These facts suggested that SGR_2427 is likely to be a β-1,2-glucan-associated enzyme. Unfortunately, SGR_2427 could not be obtained as a soluble protein (data not shown). *Sanguibacter keddieii*, a Gram-positive bacterium, also possesses a Group2 protein (SkSGL). An SkSGL encoding gene is located independently from the putative β-1,2-glucan-associated genes. SkSGL possesses additional domains with unknown functions at the *N*- and *C*-termini. A GH144 homolog from *Parabacteroides distasonis*, a common bacterium in the large intestine (Ezeji et al., 2021), with unknown-function domains at the *N*-terminus, was found to be a novel exo-type Sop_2_-releasing enzyme. The domains played a critical role in the exolytic degradation activity of the enzyme(Shimizu et al., 2018). Therefore, SkSGL was used as a target for Group 2.

A Michaelis complex structure has been reported for GH162 but not for GH144. To compare the functions, structures, and reaction mechanisms of the homologs characterized in the present study, a GH144 enzyme from *Xanthomonas campestris* pv. *campestris*, a phytopathogen (Qian et al., 2005; Vieira et al., 2021) (KEGG locus tag, XCC2207; XcSGL), was used to obtain a Michaelis complex with β-1,2-glucans.

*3. R*_work_ *and R*_free_ *values of the complex structure of XcSGL*

Because *R*_work_ *and R*_free_ values remained high after refinement, we tried other space groups including *P*4_3_2 series, *P*4_3_, *P*4, *P*23 series, *P*222 series, *P*3, *R*3 and *P*1. However, no improvement was obtained and the original space group (*P*4_3_32) was adopted.

Electron density of the XcSGL molecule is clearly observed as shown in Fig. S18A. Solvent content is over 70% in the asymmetric unit, which means that another XcSGL molecule can be put in the asymmetric unit. However, modeled XcSGL molecules form a lattice throughout the space without any unreasonable gap disturbing formation of molecular alignment potentially as shown in Fig. S18B. This allows another XcSGL molecule to be placed in different orientations to an unmodeled electron density. Thus, it is impossible to build a model for the second XcSGL molecule, which probably makes the *R*_work_ *and R*_free_ values higher than usual.

**Table S1. Classification of putative sulfatases in *P. gaetbulicola*.**

| Family / Subfamily*^a^* | UniProt accession No. | Locus |
| --- | --- | --- |
| In the gene cluster |  |  |
| S1_8 | A0A0C5WJJ0 | H744_1c0228 |
| S1_11 | A0A0C5WJC1 | H744_1c0203 |
| S1_11 | A0A0C5WJA2 | H744_1c0193 |
| S1_11 | A0A0C5WGE1 | H744_1c0221 |
| S1_11 | A0A0C5WJI5 | H744_1c0223 |
| S1_11 | A0A0C5WDX6 | H744_1c0227 |
| S1_19 | A0A0C5WQP8 | H744_1c0214 |
| S1_19 | A0A0C5WDX0 | H744_1c0217 |
| S1_19 | A0A0C5WGD5 | H744_1c0216 |
| none | A0A0C5W1R0 | H744_1c0215 |
|  |  |  |
| The others |  |  |
| S1_9 | A0A0C5WQ05 | H744_2c1766 |
| S1_13 | A0A0C5WB34 | H744_2c2132 |
| S1_13 | A0A0C5X0G6 | H744_2c2131 |
| S1_13 | A0A0C5WTU2 | H744_1c1448 |
| S1_26 | A0A0C5WSF7 | H744_1c0940 |
| S1_27 | A0A0C5WTP4 | H744_2c1768 |
| S1_44 | A0A0C5WQ21 | H744_2c1791 |
| S3 | A0A0C5W3A8 | H744_1c0866 |
| S3 | A0A0C5WU63 | H744_1c1582 |
| none | A0A0C5W3F7 | H744_1c0941 |
| none | A0A0C5WJS1 | H744_1c1445 |
| none | A0A0C5WA56 | H744_2c1790 |
| none | A0A0C5WUR0 | H744_2c2120 |
| none | A0A0C5WVI3 | H744_2c2416 |

*^a^* Classification of the family was based on SulfAtlas (Stam et al., 2023)

**Table S2. Data collection and statistics.**

| **Data set** | **EeSGL1** | **PgSGL3** | **XcSGL (E239Q)-Sop_7_** |
| --- | --- | --- | --- |
| **Data collection** |  |  |  |
| Beamline | KEK BL-5A | KEK NW-12A | KEK BL-5A |
| Space group | *P*2_1_2_1_2 | *P*2_1_2_1_2_1_ | *P*4_3_32 |
| Unit cell parameters (Å) | *a* = 110.50  *b* = 114.91  *c* = 78.89 | *a* = 52.28  *b* = 76.60  *c* = 94.43 | *a* = *b* = *c* = 220.18 |
| Resolution (Å)*^a^* | 46.44–2.40 (2.49–2.40) | 47.21–1.20 (1.22–1.20) | 49.23–2.50 (2.56–2.50) |
| Total reflections*^a^* | 529415 (57393) | 732055 (35275) | 2469753 (176787) |
| Unique reflections*^a^* | 40027 (4131) | 114009 (5320) | 63337 (4390) |
| Completeness (%)*^a^* | 100 (100) | 96.0 (91.5) | 100 (100) |
| Multiplicity*^a^* | 13.2 (13.0) | 6.4 (6.6) | 39.0 (40.3) |
| Mean *I*/σ (*I*)*^a^* | 14.1 (2.9) | 11.6 (4.0) | 35.4 (6.0) |
| *R*_merge_ (%)*^a^* | 14.1(93.2) | 9.6 (43.3) | 11.5 (87.6) |
| *R*_pim_ (%)*^a^* | 5.7 (37.2) | 6.0 (27.1) | 2.6 (19.6) |
| *CC*_1/2_*^a^* | (0.888) | (0.904) | (0.961) |
| **Refinement** |  |  |  |
| Resolution (Å) | 46.442–2.40 | 47.213–1.200 | 49.233–2.50 |
| No. of reflections | 37877 | 108263 | 60057 |
| No. of atoms | 6738 | 3819 | 3720 |
| No. of water molecules | 9 | 332 | 22 |
| *R*_work_/*R*_free_ (%) | 22.4/28.1 | 19.1/20.8 | 33.8/36.4*^b^* |
| No. of asymmetric units | 2 | 1 | 1 |
| RMSD from ideal values |  |  |  |
| Bond lengths (Å) | 0.0053 | 0.0130 | 0.0590 |
| Bond angles (°) | 1.4197 | 1.9403 | 2.0065 |
| Average *B*-factors (Å^2^) |  |  |  |
| Protein (chain A/B) | 46.2/45.4 | 11.9 | 41.9 |
| Ligand |  |  |  |
| Sop_7_ | - | - | 50.9 |
| Solvent | 34.4 | 19.6 | 33.0 |
| Ramachandran plot (%) |  |  |  |
| Favored | 95.5 | 98.1 | 95.0 |
| Allowed | 4.5 | 1.9 | 4.5 |
| Outlier | 0.0 | 0.0 | 0.5 |
| **PDB entry** | 8XUJ | 8XUK | 8XUL |

*^a^* Values in parentheses are for outer shells.

*^b^* *R* values are large because of the disorder of the *C*-terminal region compared with the catalytic domain. However, the assignment of XcSGL molecules was conducted using only the catalytic domain.

**Table S3. Residues defining SGL clan**

| Group | Enzyme | Phe | Tyr | Glu |
| --- | --- | --- | --- | --- |
| Group 1 | EeSGL1 | F274 | Y324 | E221 |
| Group 2 | SkSGL | F318 (348)*^a^* | Y373 (403) | E246 (276) |
| Group 3 | PgSGL3 | F274 | Y333 | E214 |
| Group 4 | PgSGL4 | F241 | Y297 | E193 |
| GH144 | XcSGL | F286 (298)*^a^* | Y367 (379) | E239 (251) |
| GH144 | CpSGL | F268 | Y330 | E211 |
| GH162 | TfSGL | H316* | Y373 | E262 |
| GH189 | TiCGS_Tg_ | F1399 | Y1456 | E1356 |

*^a^* The residue numbers in parentheses are based on the sequence in the database.

* Asterisk represents that a residue type is not conserved.

**Table S4. Primers used in the study.**

| Name | Sequence (5′ to 3′)*^a^* | Restriction enzyme |
| --- | --- | --- |
| Cloning |  |  |
| PgSGL1 Fw | GTATCTCGAGCCACATAACATCCAAGTTC | XhoI |
| PgSGL1 Rv | CGGCGAATTCTTATTCGTTATTCGGAGTAAAC | EcoRI |
| PgSGL2 Fw | GATACTCGAGCTTGGTGGATGTGCATCGAGTG | XhoI |
| PgSGL2 Rv | GCCGGAATTCCTACGAAGCGTTTTCCTCACG | EcoRI |
| PgSGL3 Fw | GATGGCACATATGCAAACGGCGGCAAATG | NdeI |
| PgSGL3 Rv | GACGTCTCGAGCTCAAAGTAGTTGTTG | XhoI |
| EeSGL1 Fw | GTATCTCGAGAGCGCTGTCACACTGGATAAACTG | XhoI |
| EeSGL1 Rv | GCCGGAATTCTTACTTAGCCCGGTCGTCTTTAAAAC | EcoRI |
| PgSGL4 Fw | TATCGAAGGTAGGCATATGTGTGGTGGTGGCTCTGGC |  |
| PgSGL4 Rv | TTTAAGCAGAGATTACCTAGTAGCACTGAGCACTATTATTAC |  |
| XcSGL Fw | CTGCAAACATATGGAGGAGCCCAAG | NdeI |
| XcSGL Rv | AAGGCGCACCTCGAGCTCGGGTTTGGG | XhoI |
| SkSGL Fw | AAAAAAACATATGGGATCCCCCACCCGCGG | NdeI |
| SkSGL Rv | AATCGTGGCGGCCGCGTCCCGGCGACGCGTCCGGA | NotI |
| SkSGL Δsignal Fw | ACATATGGCGCCCACGCCCGAGCAG |  |
| SkSGL Δsignal Rv | GTGGGCGCCATATGTATATCTCCTTC |  |
| SkSGL pCold Fw | TATCGAAGGTAGGCATATGGCGCCCACGCCCGAGCAGACA |  |
| SkSGL pCold Rv | TTTAAGCAGAGATTACCTAGTCCCGGCGACGCGTCCGGA |  |
| pCold I Fw | TAGGTAATCTCTGCTTAAAAGCAC |  |
| pCold I Rv | CATATGCCTACCTTCGATATGATG |  |
|  |  |  |
| Mutants |  |  |
| PgSGL3 E214Q Fw | TTCAATCAATATATGCTTGTTGCCGGT |  |
| PgSGL3 E214Q Rv | CATATATTGATTGAACGCTTTGGTACG |  |
| XcSGL E239Q Fw | TACAACCAAGCCATGATGCTGTACATC |  |
| XcSGL E239Q Rv | CATGGCTTGGTTGTAGCCCATCCAGTC |  |
| EeSGL1 D149N Fw | GCTGTTAACAATGGCAACCTGGCGTTT |  |
| EeSGL1 D149N Rv | GCCATTGTTAACAGCTGGCGCAATTTC |  |
| EeSGL1 E221Q Fw | GCCAATCAAAGCCGACTGGCGGCACTC |  |
| EeSGL1 E221Q Rv | TCGGCTTTGATTGGCCTTGCGATCCAC |  |
| PgSGL3 D148N Fw | CCGGTTAACAGTGCCATCATGATTTAT |  |
| PgSGL3 D148N Rv | GGCACTGTTAACCGGACTCCAGTCATC |  |
| PgSGL3 E214Q Fw | TTCAATCAATATATGCTTGTTGCCGGT |  |
| PgSGL3 E214Q Rv | CATATATTGATTGAACGCTTTGGTACG |  |

*^a^* Restriction sites for NdeI and XhoI are underlined.

**Table S5. Theoretical molecular masses and extinction coefficients of the recombinant proteins.**

|  | Theoretical molecular mass (g/mol) | Extinction coefficient (A_280_ cm^−1^ M^−1^) |
| --- | --- | --- |
| PgSGL1 | 51432.45 | 72810 |
| PgSGL2 | 53698.186 | 89395 |
| EeSGL1 | 50508.48 | 99240 |
| PgSGL3 | 48951.656 | 91010 |
| SkSGLc*^b^* | 73667.049 | 118190 |
| SkSGLn*^b^* | 77634.668 | 118315 |
| XcSGL | 60466.116 | 128230 |
| PgSGL4 | 126166.348 | 164155 |


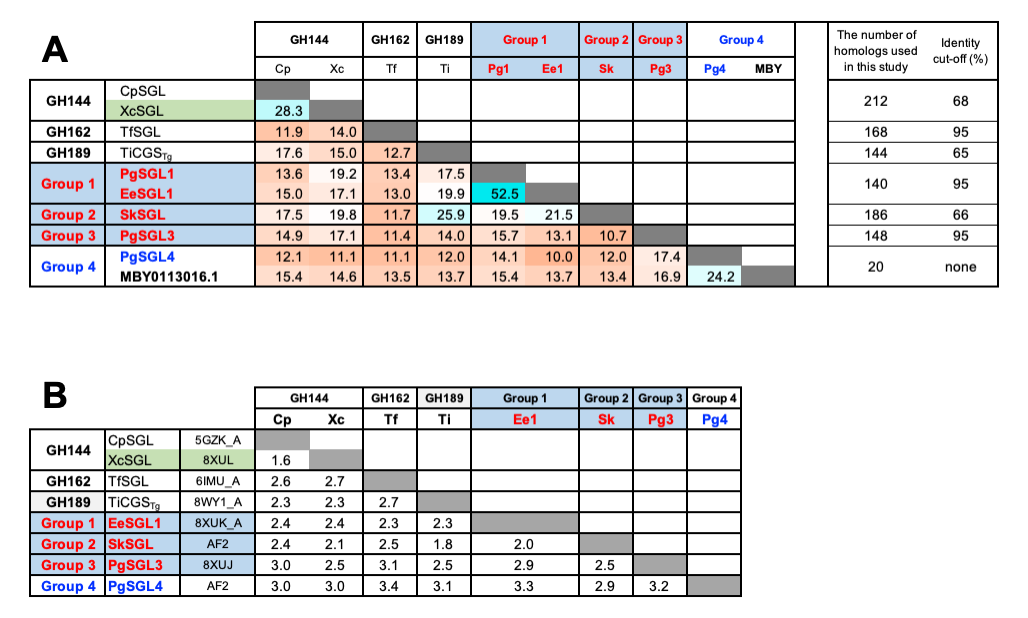


**Figure S1. Matrices of SGL clan**

The enzymes in Groups 1–3 are shown in bold red letters and are highlighted in light blue. XcSGL, a biochemically identified enzyme in this study is highlighted in light green. Shortened names are used as column names for the clan proteins. Only the (α/α)_6_-barrel domain regions were used; XcSGL, residues 72–498 a.a.; SkSGL, residues 42–514 a.a.; PgSGL4, residues 347–754 a.a. (*C*-terminus); MBY0113016.1, residues 379–774 a.a. (*C*-terminus). (A) Identity matrix of the SGL clan. Amino acid sequence identities are shown as percentages (%) in the matrix. These values are colored as a heatmap; the maximum value, cyan; 20%, white; 0%, red. (B) RMSD matrix. The RMSD values are in angstroms. These values were evaluated using the DALI server (Holm, 2022). PDB and chain IDs are shown beside the protein names. AF2 indicates that the catalytic domains of SkSGL and PgSGL4 predicted by AlphaFold Colab were used for comparison.


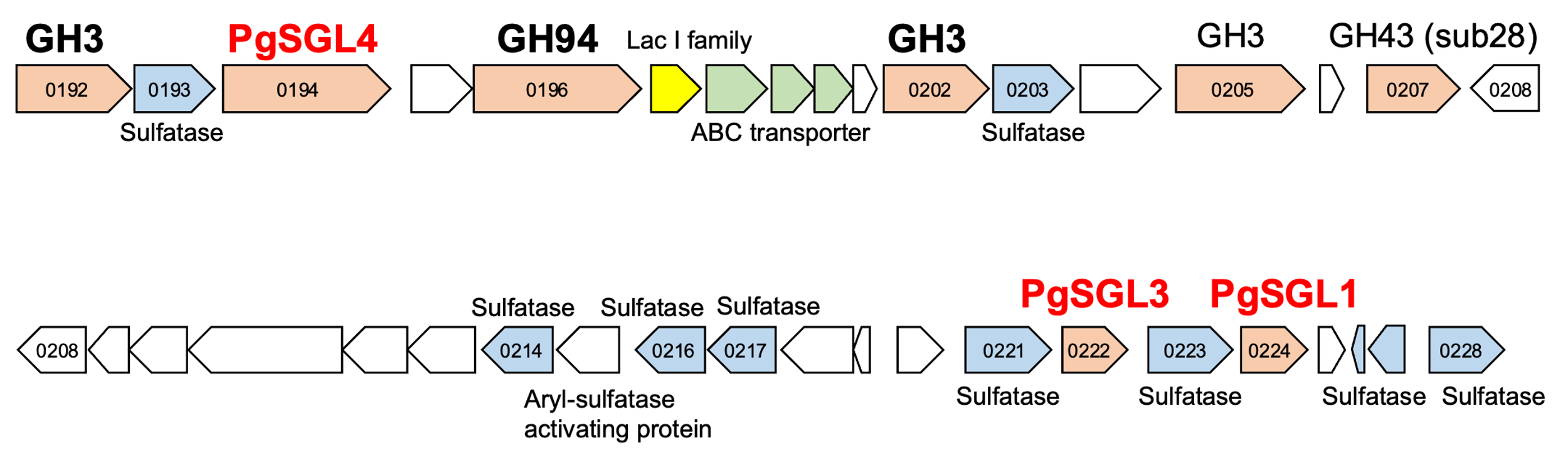


**Figure S2. A gene cluster of genes encoding PgSGL1, PgSGL3, and PgSGL4 from *P. gaetbulicola***

The directions of genes are represented as arrows. The arrow boxes for genes registered in CAZy and SulfAtlas databases (Barbeyron et al., 2016; Drula et al., 2022; Levasseur et al., 2013; Stam et al., 2023) are colored light red and light blue, respectively. The arrow boxes for putative ABC transporter genes and a putative LacI family transcriptional regulator gene are colored light green and yellow, respectively. KEGG locus tags with the “H744_1c”, common letters in the loci omitted are shown in the arrow boxes for genes registered in CAZy or SulfAtlas databases. H744_1c0208 is duplicated to show that the genes in the first and the second lines are shown in the same scale. The target proteins in this study are labelled in bold red letters. Proteins with biochemical functions confidently presumed are labelled in bold black letters.


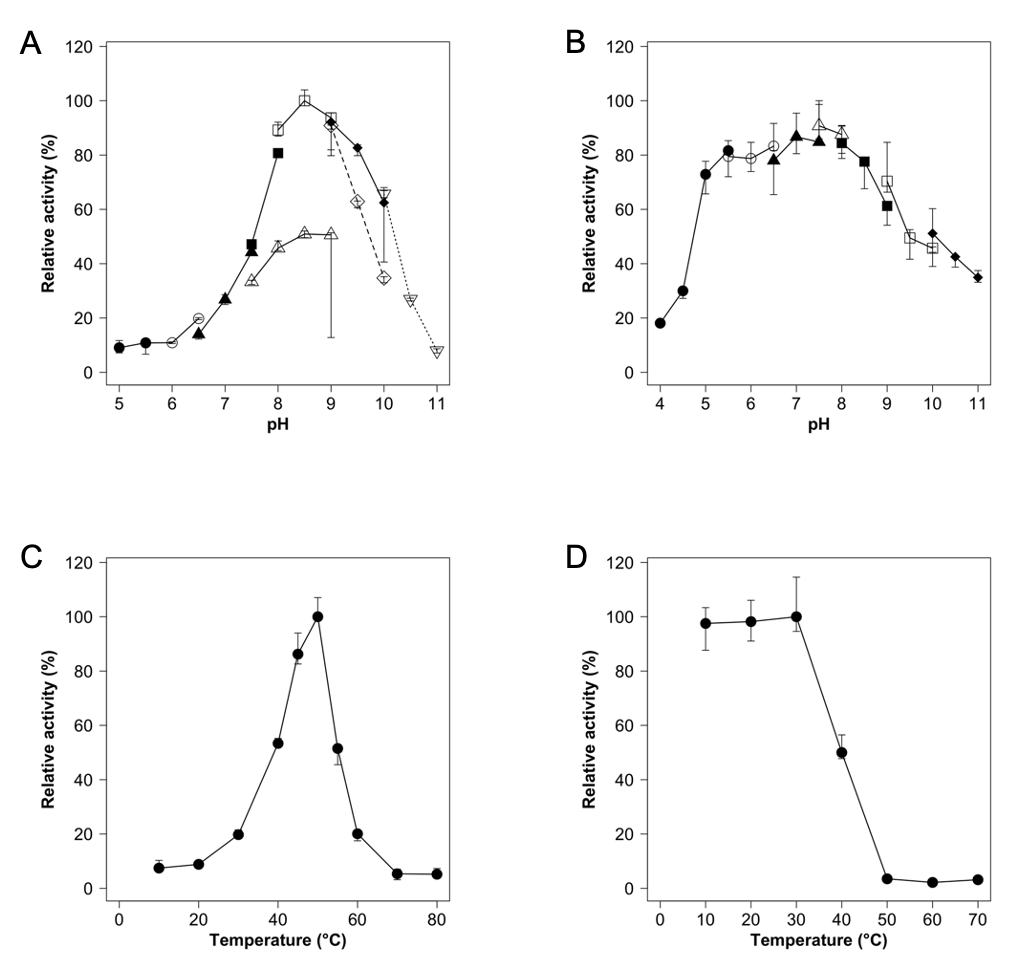


**Figure S3. pH and temperature profiles of PgSGL1 (Group 1)**

The median values of triplicate experiments are plotted as symbols and the other data are shown as error bars. (A, B) investigation of optimal pH (A) and stability (B). Symbols used for optimal pH are closed circles (sodium acetate, pH 5.0–5.5), open circles (MES-NaOH, pH 5.5–6.5), closed triangles (MOPS-NaOH, pH 6.5–7.5), open triangles (Tris-HCl, pH 7.5–9.0), closed squares (HEPES-NaOH, pH 7.5–8.0), open squares (bicine-NaOH, pH 8.0–9.0), closed diamonds (glycine-NaOH, pH 9.0–10.0), open diamonds (CHES-NaOH, pH 9.0–10.0), and open inverted triangles (CAPS-NaOH, pH 10.0–11.0). The counter-ions of the buffers are omitted hereafter. Symbols used for pH stability are closed circles (sodium acetate, pH 4.0–5.5), open circles (MES, pH 5.5–6.5), closed triangles (MOPS, pH 6.5–7.5), open triangles (HEPES, pH 7.5–8.0), closed squares (bicine, pH 8.0–9.0), open squares (glycine, pH 9.0–10.0), and closed diamonds (CAPS, pH 10.0–11.0). (C, D) Optimal temperature (C) and stability (D). Closed circles indicate the median values.


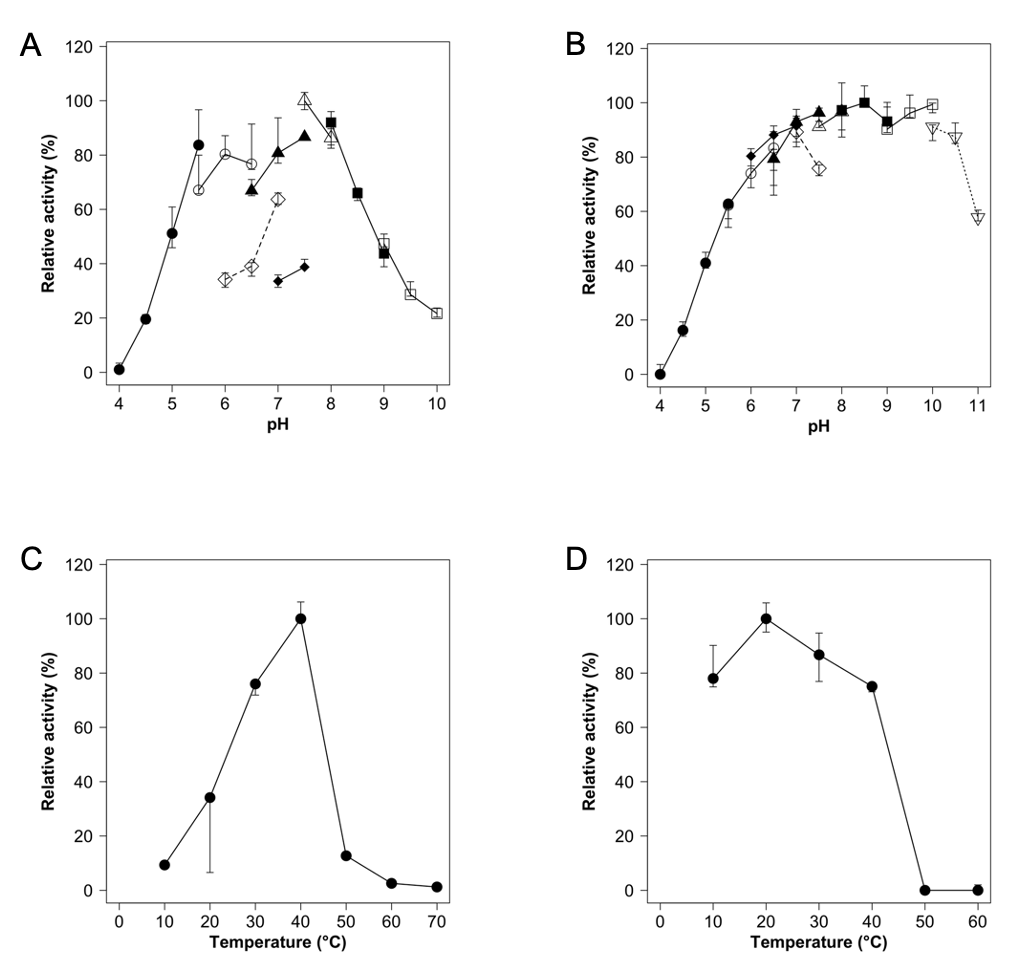


**Figure S4. pH and temperature profiles of PgSGL2 (Group 1)**

The median values of triplicate experiments are plotted as symbols and the other data are shown as error bars. (A, B) investigation of optimal pH (A) and stability (B). Symbols used are closed circles (sodium acetate, pH 4.0–5.5), open circles (MES, pH 5.5–6.5), closed triangles (MOPS, pH 6.5–7.5), open triangles (HEPES, pH 7.5–8.0), closed squares (bicine, pH 8.0–9.0), open squares (glycine, pH 9.0–10.0), closed diamonds (bis-Tris, pH 7.0–7.5), open diamonds (bis-tris propane-HCl, pH 6.0–7.0), and open inversed triangles (CAPS, pH 10.0–11.0). (C, D) Optimal temperature (C) and stability (D). Closed circles indicate the median values.


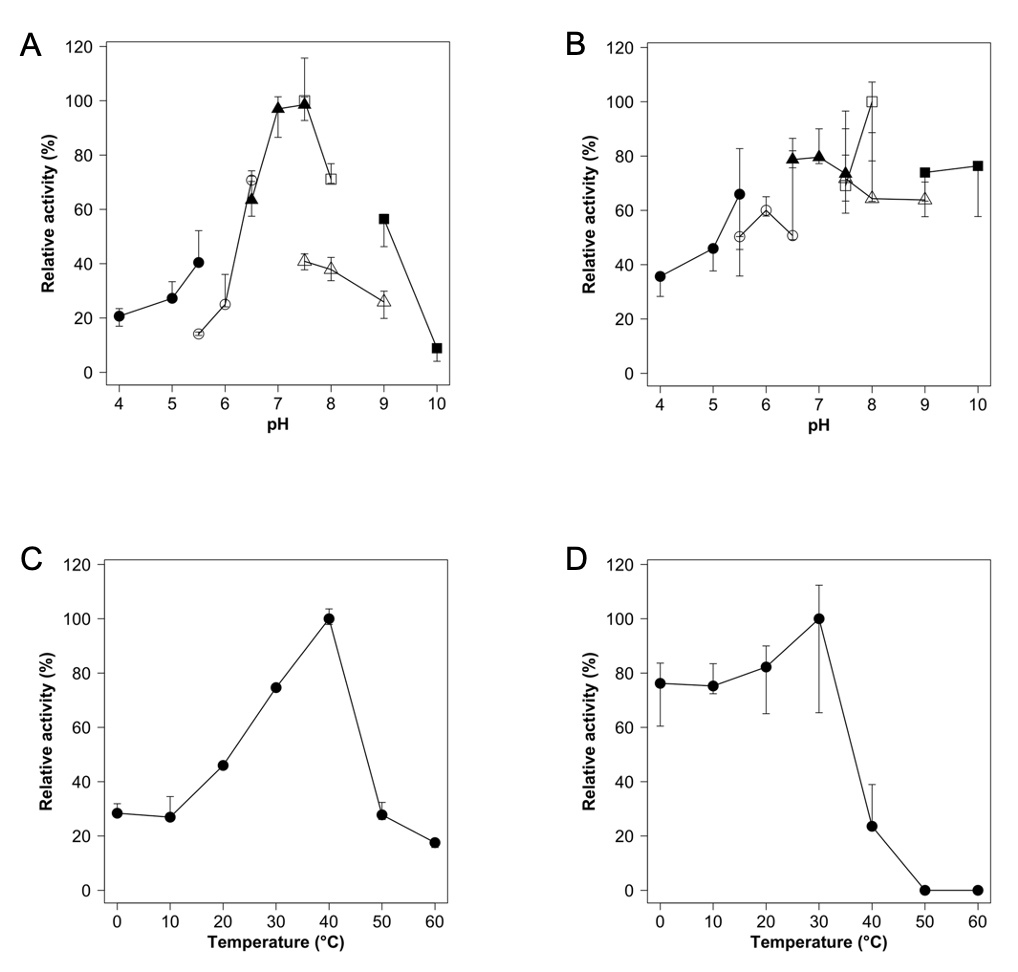


**Figure S5. pH and temperature profiles of EeSGL1 (Group 1)**

The median values of triplicate experiments are plotted as symbols and the other data are shown as error bars. (A, B) Investigation of optimal pH (A) and stability (B). Symbols used are closed circles (sodium acetate, pH 4.0–5.5), open circles (MES, pH 5.5–6.5), closed triangles (MOPS, pH 6.5–7.5), open triangles (Tris, pH 7.5–9.0), closed squares (glycine, pH 9.0–10.0), and open squares (HEPES, pH 7.5–8.0). (C, D) Optimal temperature (C) and stability (D). Closed circles indicate the median values.


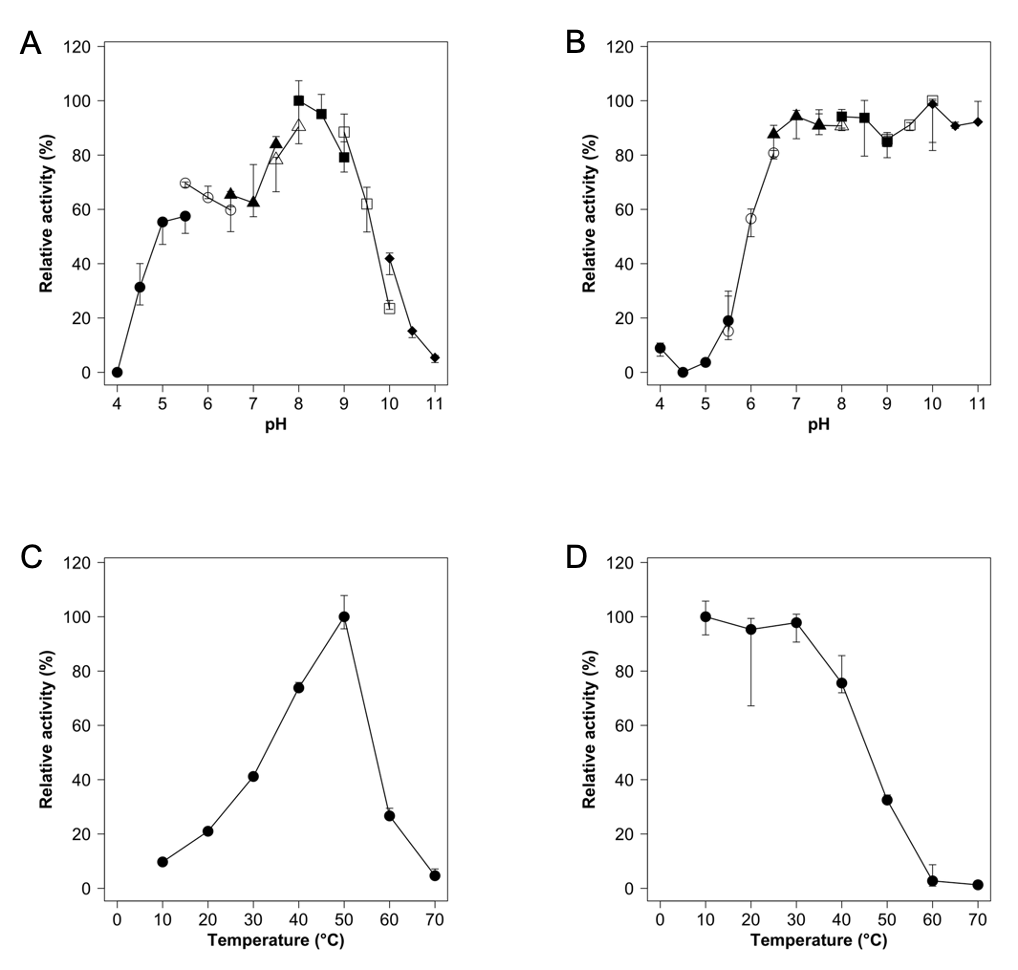


**Figure S6. pH and temperature profiles of SkSGL (Group 2)**

The median values of triplicate experiments are plotted as symbols and the other data are shown as error bars. (A, B) Investigation of optimal pH (A) and stability (B). Symbols used are closed circles (sodium acetate, pH 4.0–5.5), open circles (MES, pH 5.5–6.5), closed triangles (MOPS, pH 6.5–7.5), open triangles (HEPES, pH 7.5–8.0), closed squares (bicine, pH 8.0–9.0), open squares (CHES, pH 9.0–10.0), and closed diamonds (CAPS, pH 10.0–11.0). (C, D) Optimal temperature (C) and stability (D). Closed circles indicate the median values.


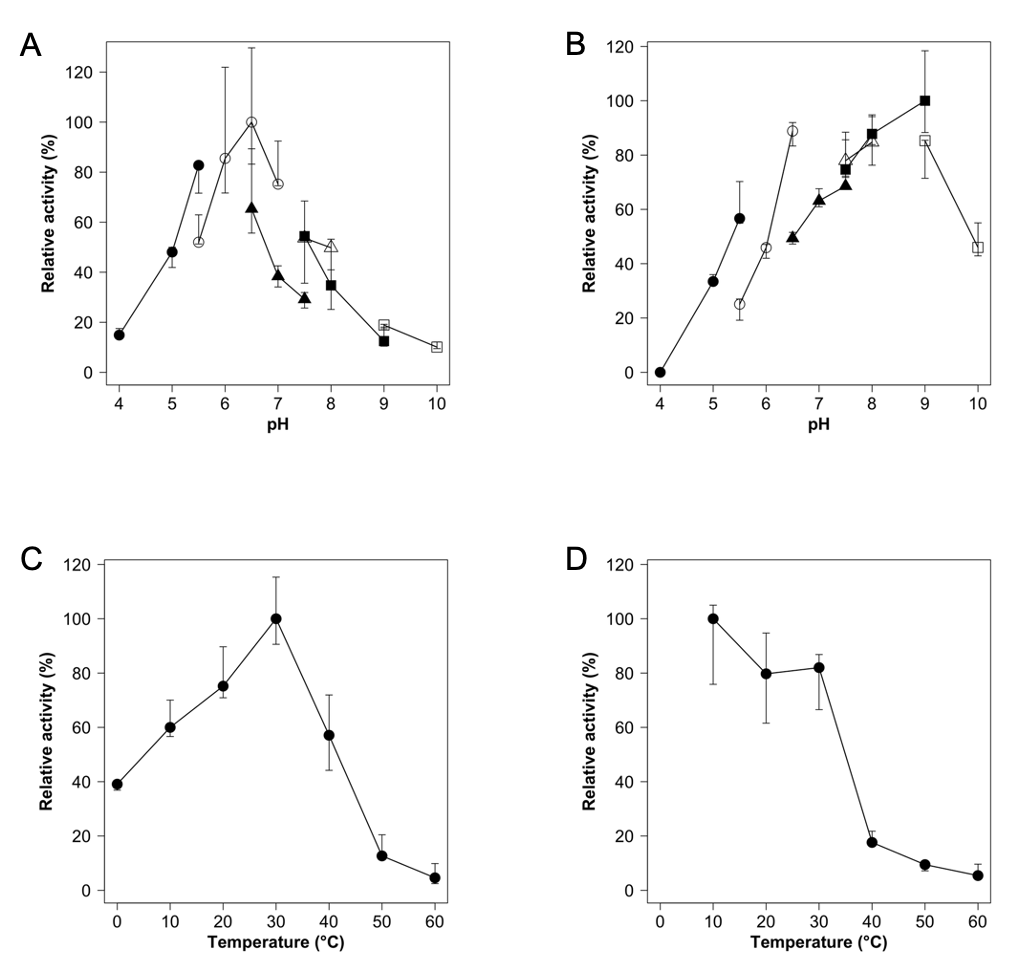


**Figure S7. pH and temperature profiles of PgSGL3 (Group 3)**

The median values of triplicate experiments are plotted as symbols and the other data are shown as error bars. (A, B) Investigation of the optimal pH (A) and stability (B). Symbols used are closed circles (sodium acetate, pH 4.0–5.5), open circles (sodium cacodylate, pH 5.5–7.0), closed triangles (MOPS, pH 6.5–7.5), open triangles (HEPES, pH 7.5–8.0), closed squares (Tris, pH 7.5–9.0), and open squares (glycine, pH 9.0–10.0). (C, D) Optimal temperature (C) and stability (D). Closed circles indicate the median values.


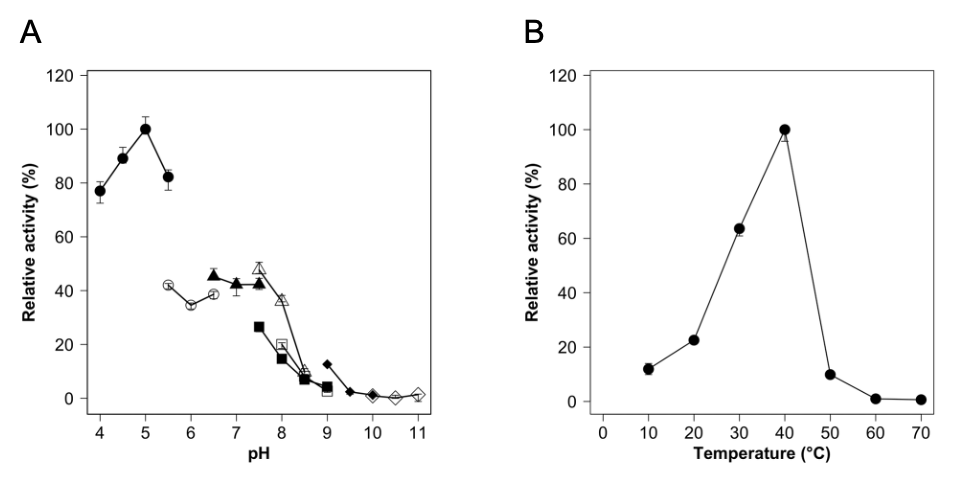


**Figure S8. Optimal pH and temperature of XcSGL (GH144)**

The median values of triplicate experiments are plotted as symbols and the other data are shown as error bars. (A) Investigation of the optimal pH. Symbols used are closed circles (sodium acetate, pH 4.0–5.5), open circles (MES, pH 5.5–6.5), closed triangles (MOPS, pH 6.5–7.5), open triangles (HEPES, pH 7.5–8.5), closed squares (Tris, pH 7.5–9.0), open squares (bicine, pH 8.0–9.0), closed diamonds (glycine, pH 9.0–10.0), and open diamonds (CAPS, pH 10.0–11.0). (B) Investigation of the optimal temperature. Closed circles indicate the median values.


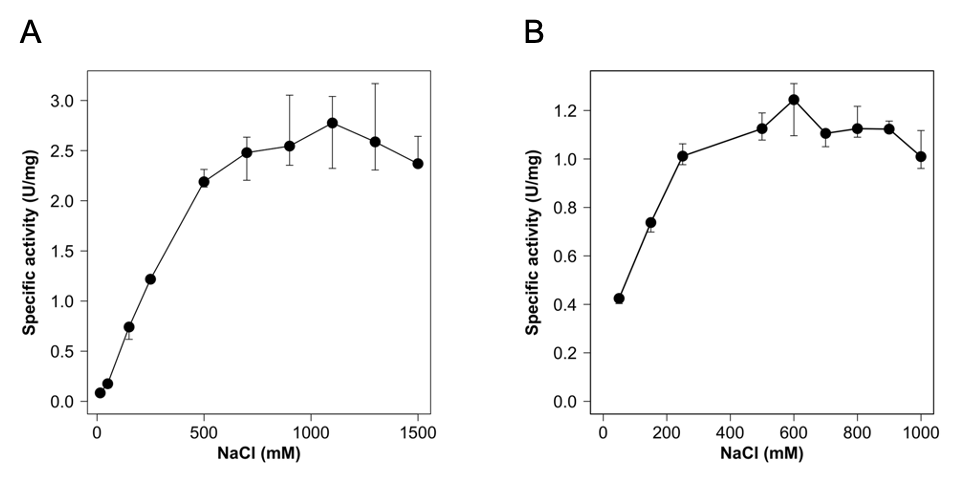


**Figure S9. Effect of NaCl on the hydrolytic activity of PgSGL2**

Hydrolytic activity (A) and stability (B) of PgSGL2 in the presence of various concentrations of NaCl. Medians in the triplicate experiments are plotted as closed circles and the other data are shown as error bars.


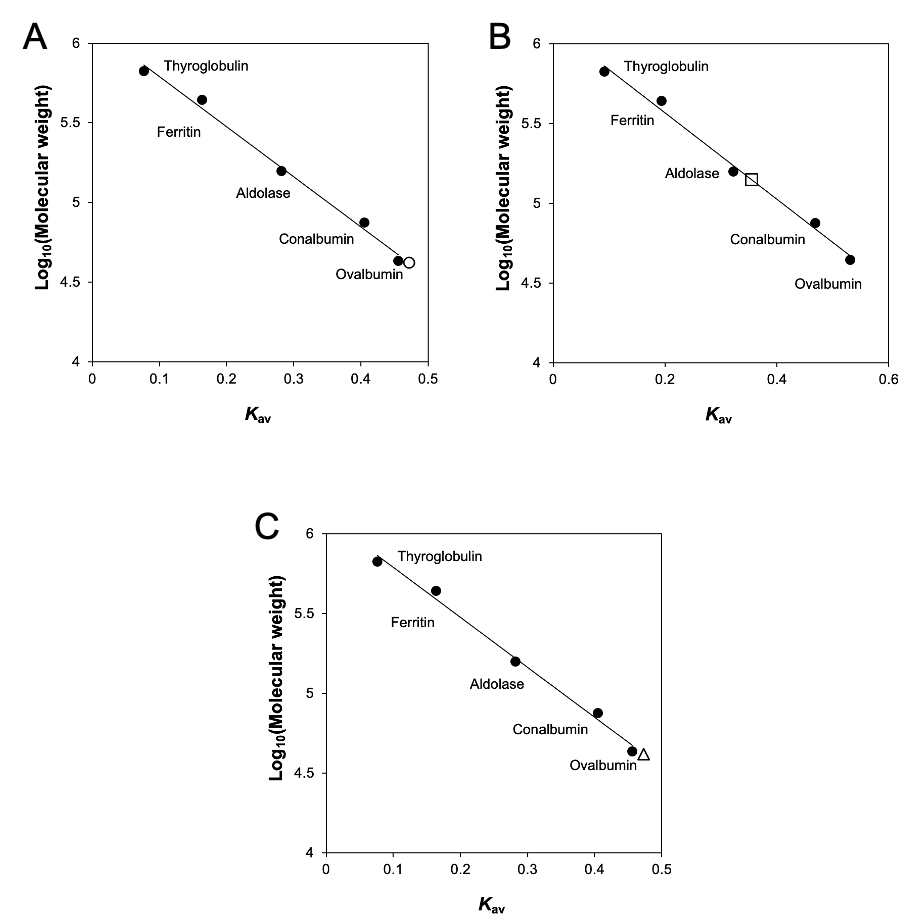


**Figure S10. Size-exclusion chromatography analysis**

(A), EeSGL1 (Group 1); (B), SkSGL (Group2); and (C), PgSGL3 (Group 3). Protein standard markers are shown as closed circles. EeSGL1, SkSGL, and PgSGL3 are shown as open circle, square, and triangle, respectively.


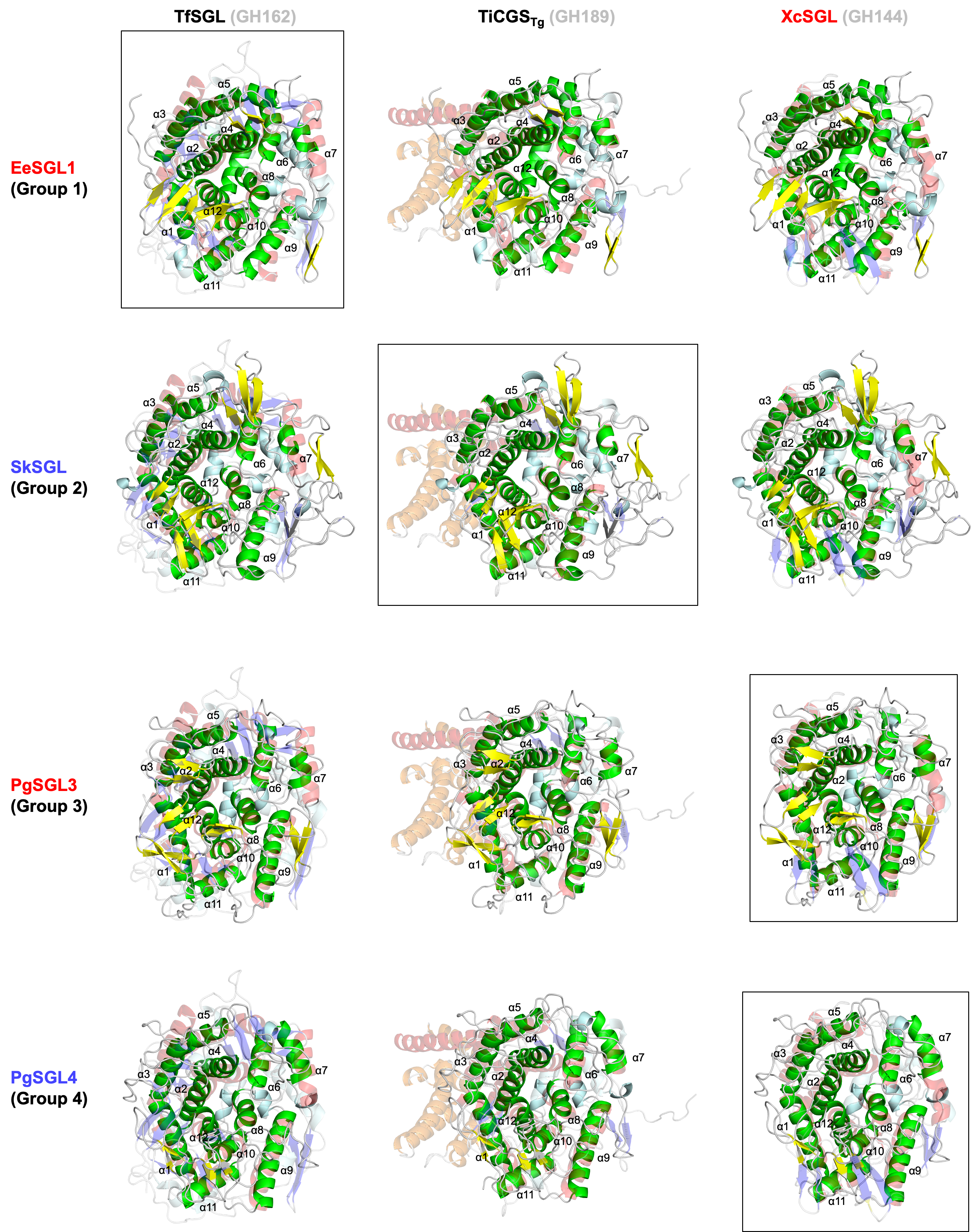


**Figure S11. Superimposition of overall structures between Groups 1–4 and the known families, GH144, GH162 and GH189**

The structures are shown in the same way as Fig. 6. The boxed structures are the same as those used in Fig. 6.


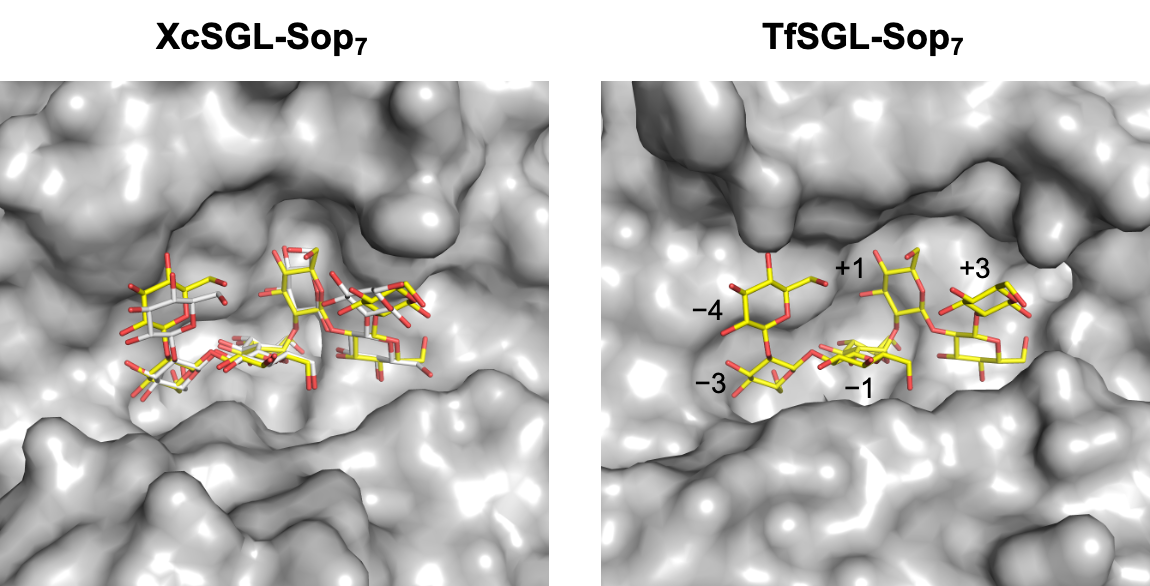


**Figure S12. Comparison of substrate pockets between XcSGL-Sop_7_ and TfSGL-Sop_7_ complexes.**

E239Q mutant of XcSGL and E262Q mutant of TfSGL are used for preparation. XcSGL and TfSGL are shown as gray surfaces. Sop_7_ molecules in XcSGL and TfSGL are shown as white and yellow sticks, respectively.


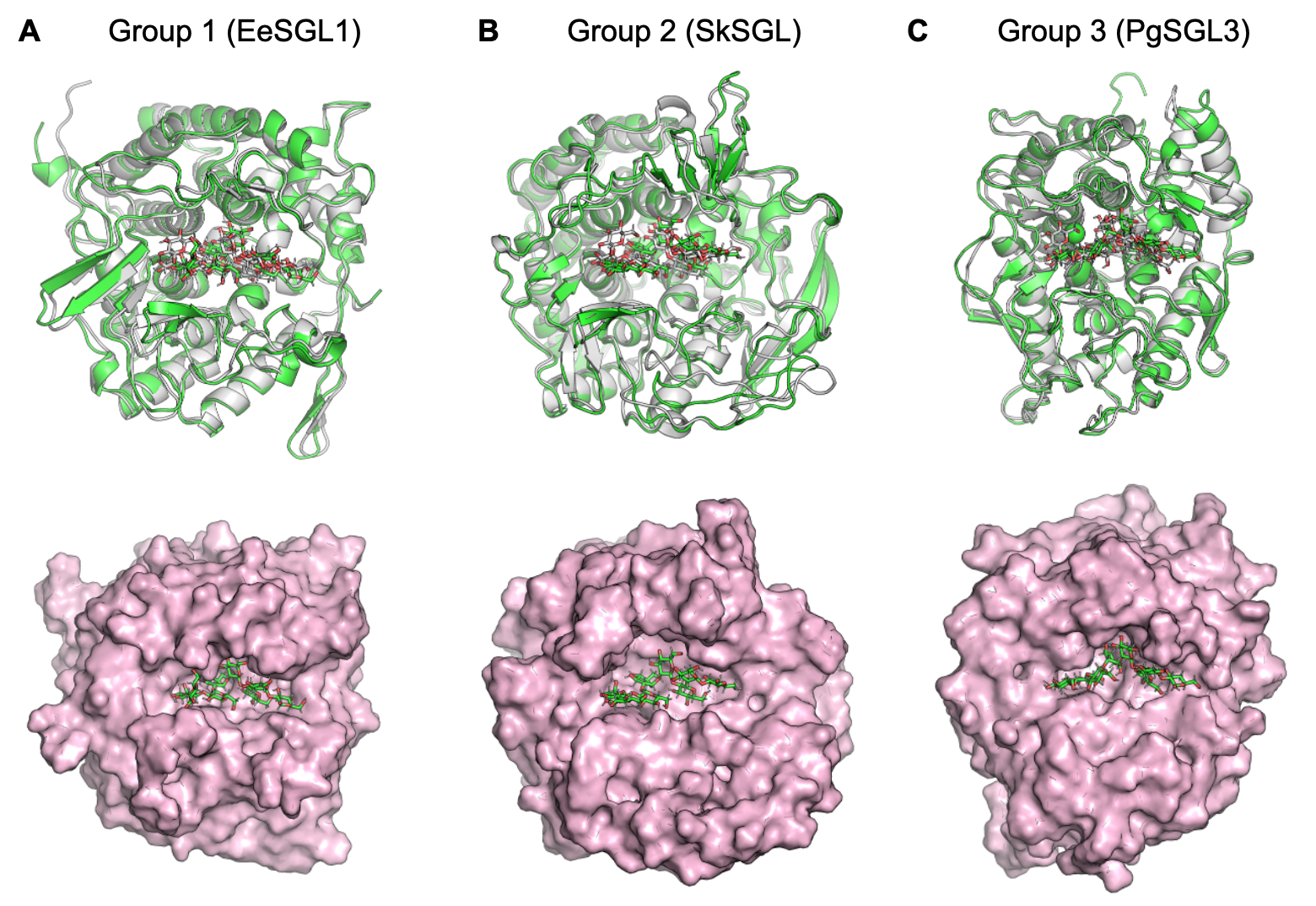


**Figure. S13. Structures of the complexes obtained by MD simulations**

(A–C) Structures before and after MD simulations are colored white and green, respectively. (top) Proteins and substrates are shown as cartoons and sticks, respectively. (bottom) Final structures in the MD simulations. The proteins and substrates are shown with pale pink surfaces and as green sticks, respectively. (C) A chloride ion is shown as a sphere.


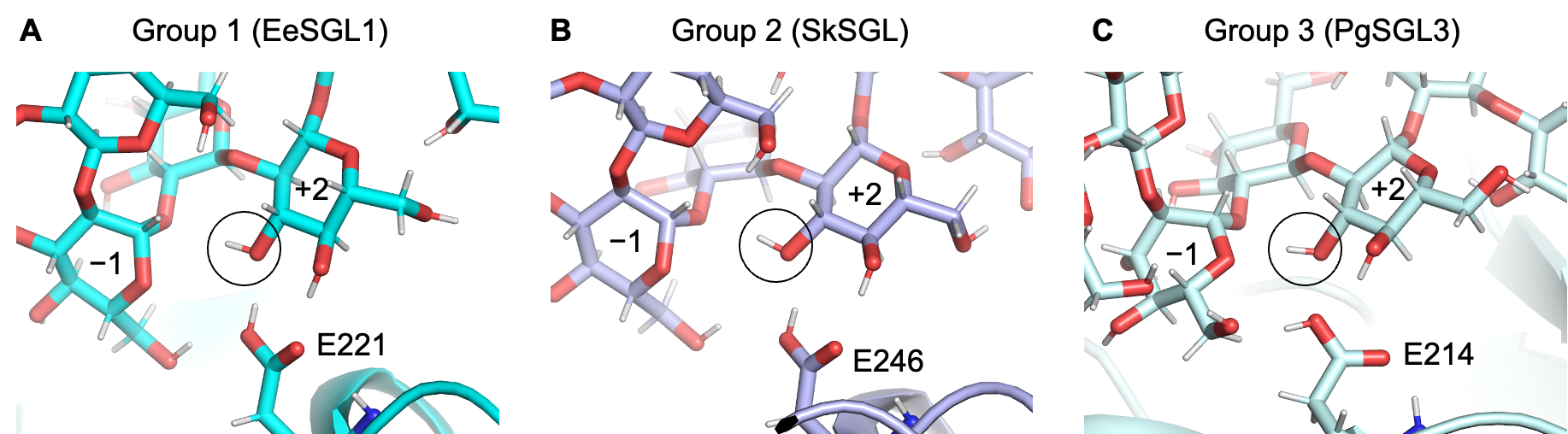


**Figure S14. Substrate recognition by the catalytic residues in MD simulation**

Substrates (Sop_8_ molecules) and proteins (A, EeSGL1; B, SkSGL; and C, PgSGL3). EeSGL1, SkSGL, and PgSGL3 are colored the same as the corresponding proteins in Fig. 5 (cyan, light purple, and pale cyan, respectively). The complex structures are the final structures in the MD simulations. 3-Hydroxy groups are indicated by black circles. The candidates for general acids are shown as sticks. Subsite numbers are shown at the substrates.


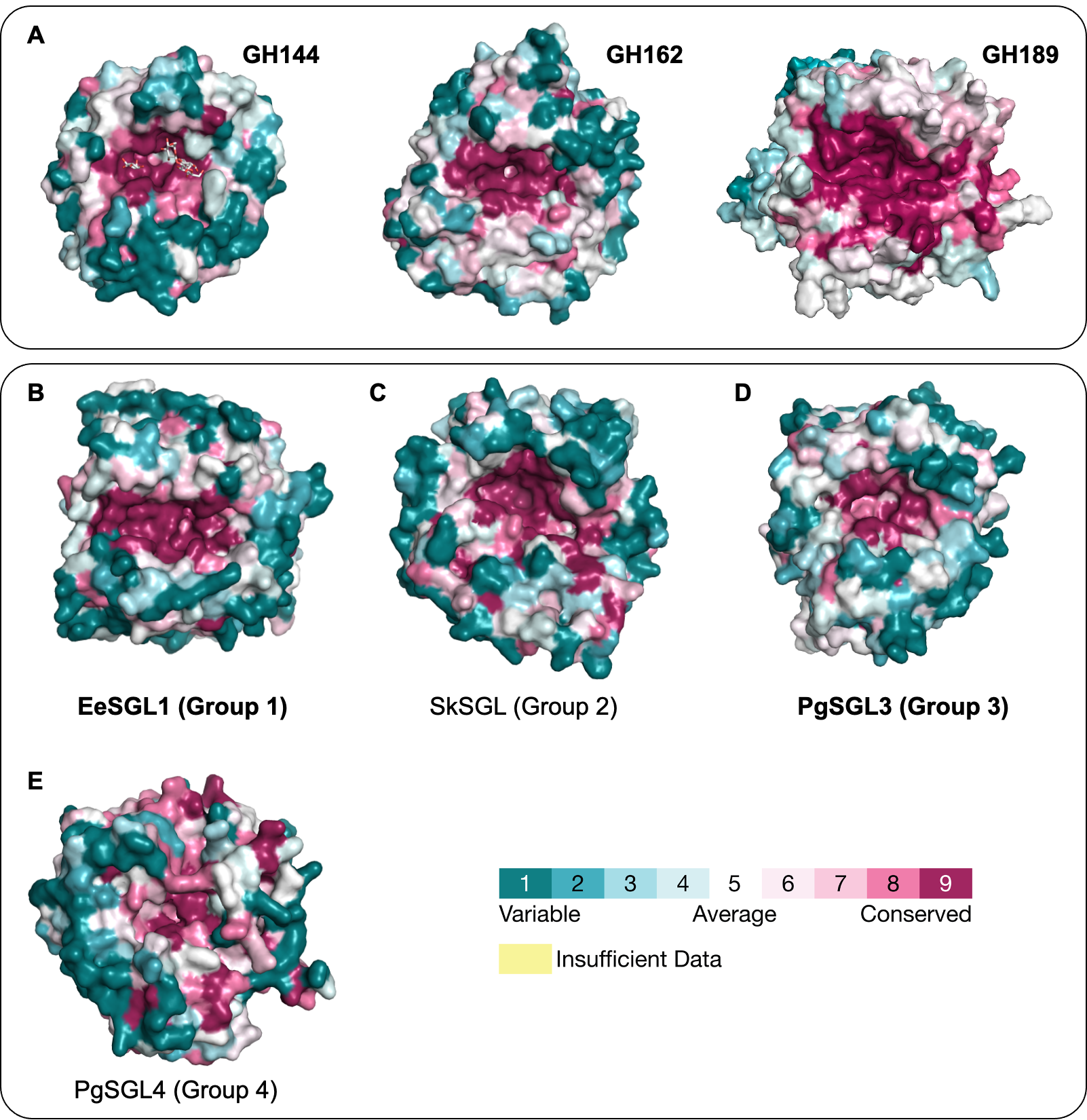


**Figure S15. Surface models of the SGL-clan proteins**

The SGL-clan proteins are shown with the surfaces colored based on the conservation scores. The conservation scores were calculated by ConSurf using the structures shown in the figure. Proteins with over 35% amino acid sequence identity with query extracted from UNIREF-90 were used for calculation of conservation. The conservation scores are colored according to the color bar provided in the ConSurf server (Ashkenazy et al., 2016; Landau et al., 2005). GH families or groups with experimentally solved structures and those with structures only predicted by AlphaFold2 are shown in bold and standard type, respectively. (A) GH families reported previously; (left) CpSGL (GH144, 5GZK), (middle) TfSGL (GH162; PDB ID, 6IMU), and (right) TiCGS_Tg_ (GH189, prediction). The root mean square deviation between the crystal structure of TiCGS_Tg_ (PDB ID, 8WY1) and the predicted structure was 0.54 Å. (B–E) Groups identified in this study. (B) EeSGL1 (Group 1, PDB ID, 8XUJ). (C) SkSGL (Group 2, prediction). (D) PgSGL3 (Group 3, PDB ID, 8XUK). (E) PgSGL4 (Group 4, prediction). “prediction” indicates query structures were predicted by AlphaFold2 (Jumper et al., 2021).


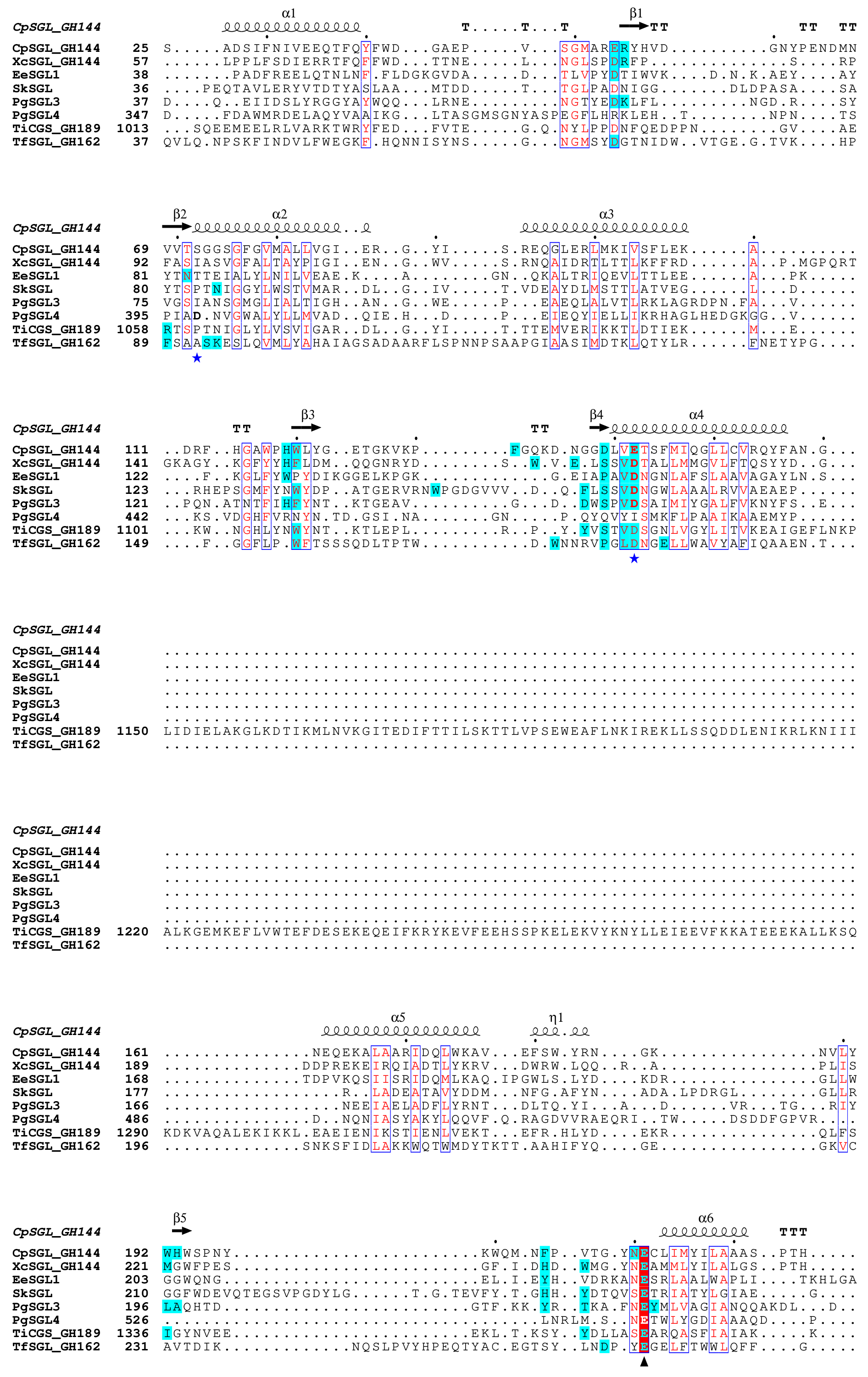


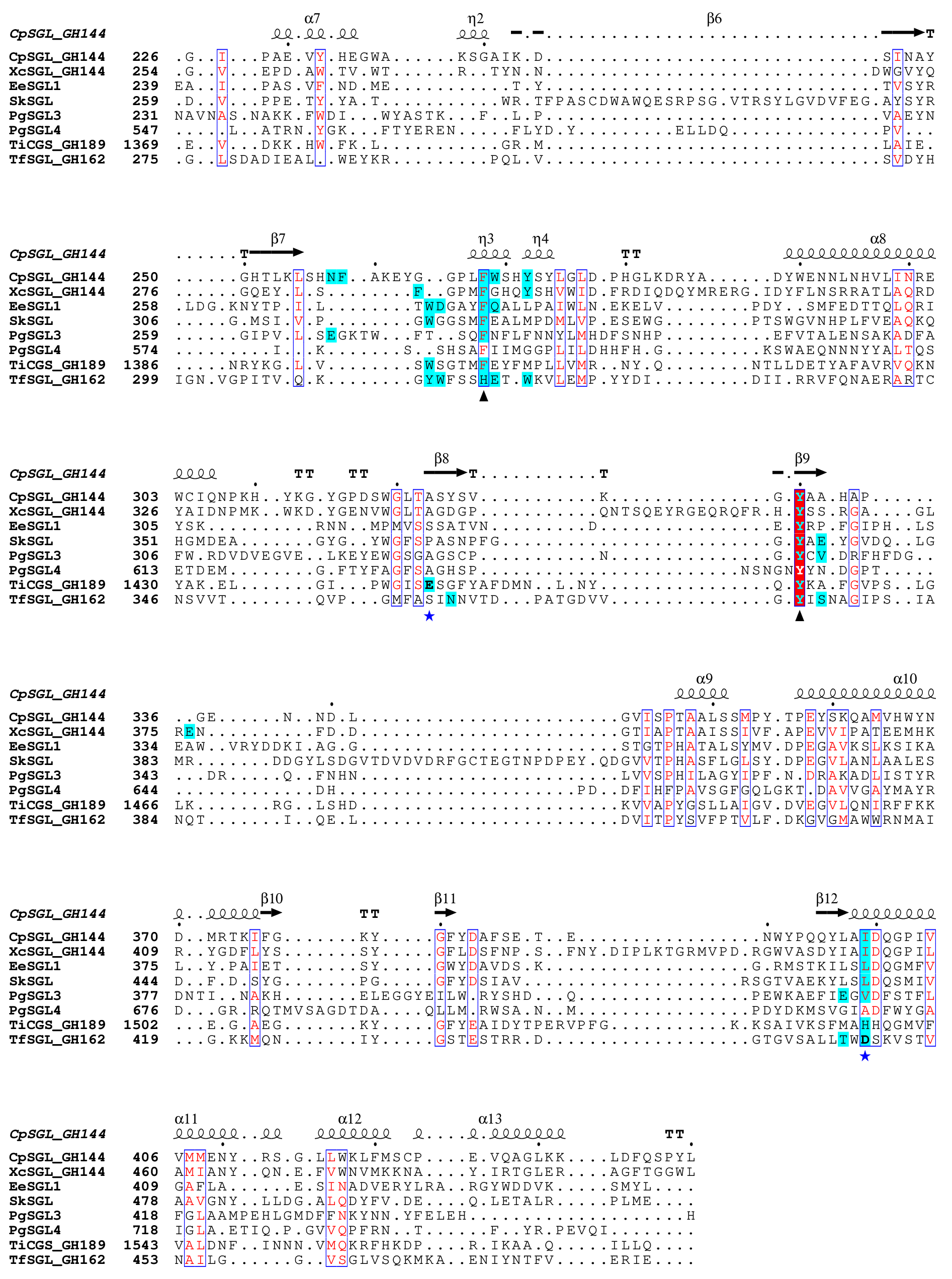


**Fig. S16. The entire multiple sequence alignment of the SGL-clan proteins**

A version of the entire multiple alignment shown in Fig. 7. Residues in bold letters labelled with a blue star are a general base catalyst for TfSGL, a nucleophile for TiCGS_Tg_, and candidate general base catalysts for the other proteins. The residues that define the SGL-clan proteins are labelled with black triangles. Residues within 4 Å of the superimposed Sop_7_ molecule from the XcSGL (E239Q)-Sop_7_ complex are colored cyan.


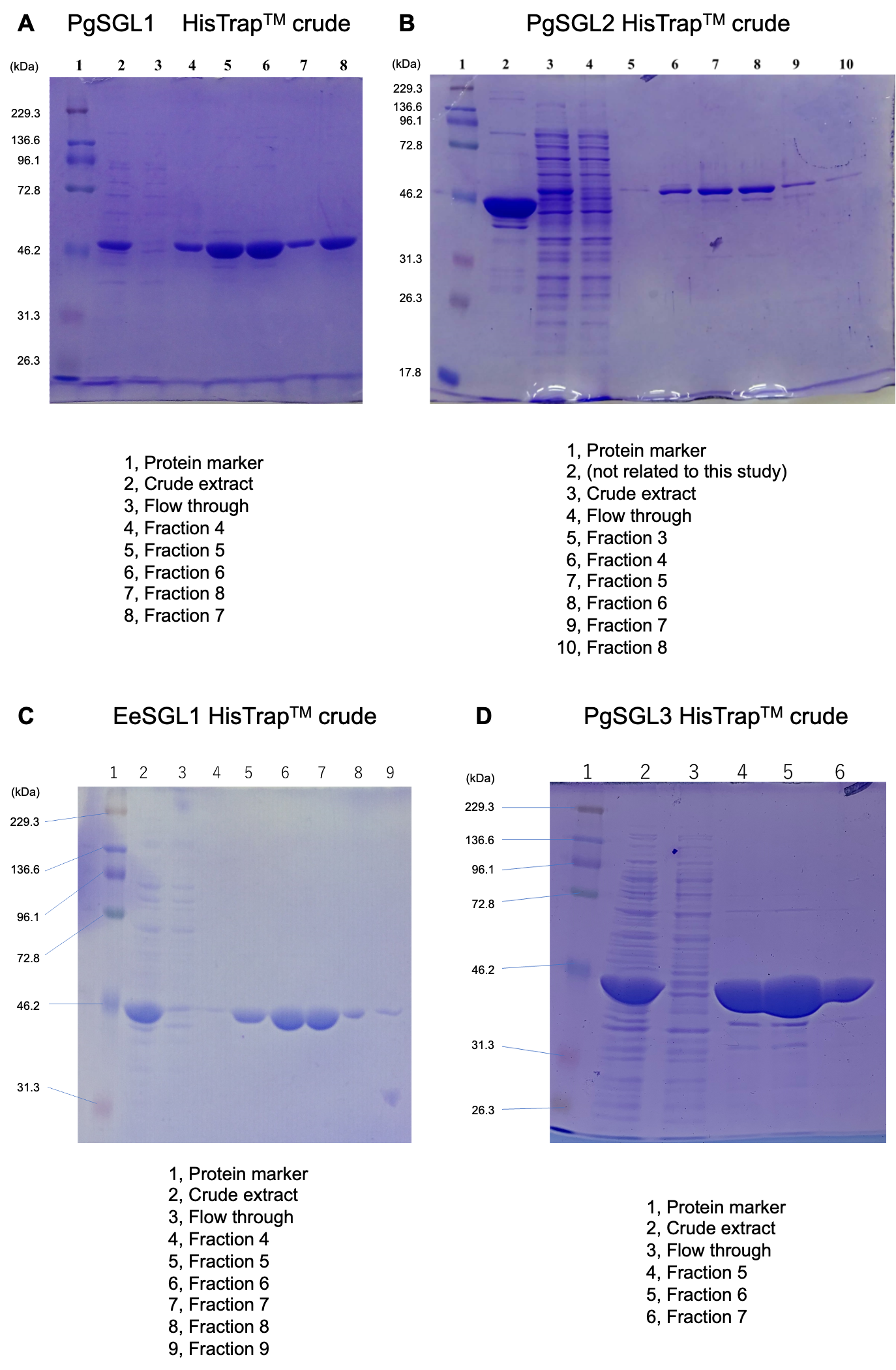


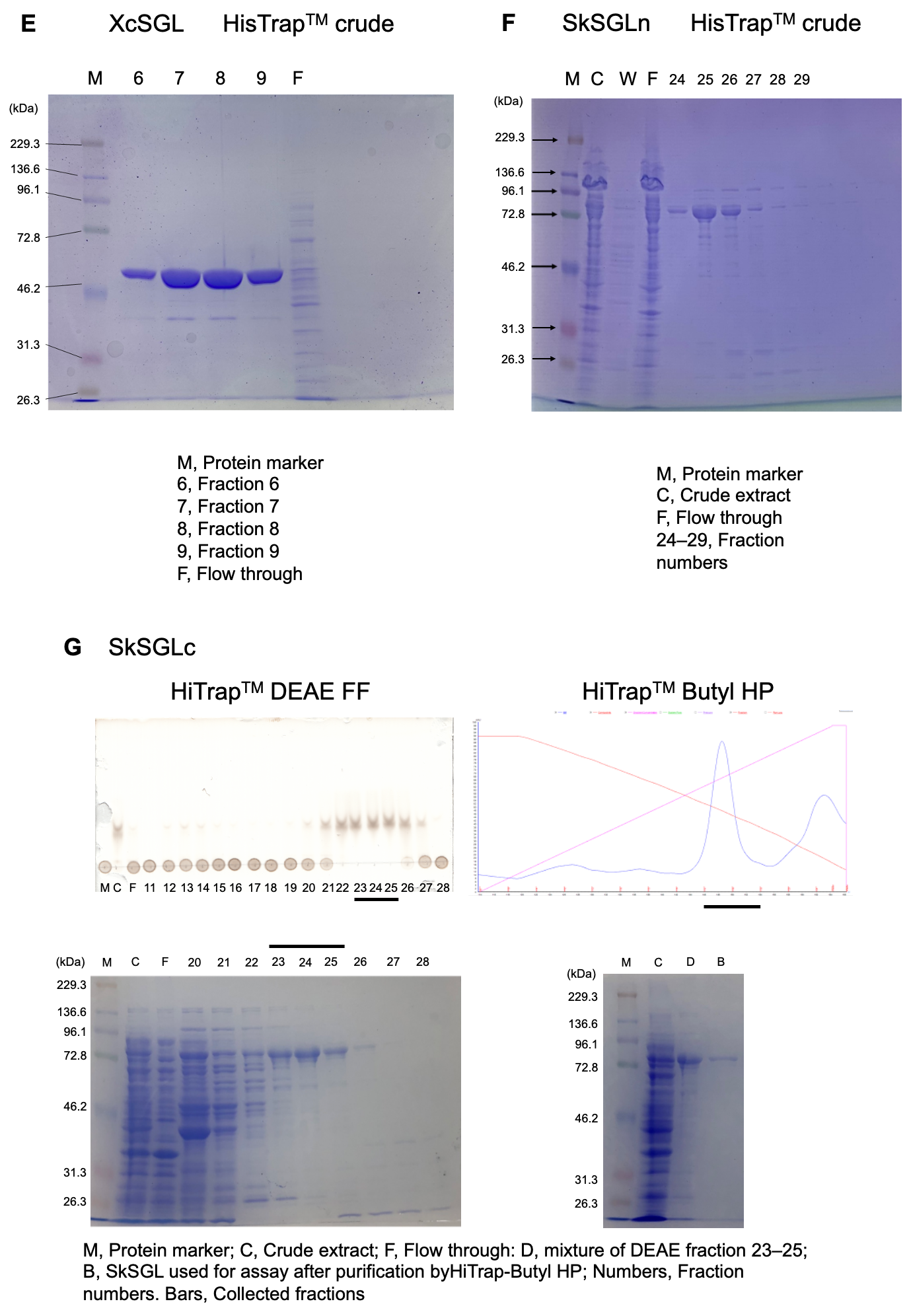


**Figure S17. Purification of SGL-clan enzymes**

The enzymes and columns used for purification are shown above the SDS-PAGE gels, TLC plate, and chromatography chart. Descriptions of the lanes for SDS-PAGE are shown below the SDS-PAGE gels. Molecular weights of the protein markers are shown beside the SDS-PAGE gels. (G) (top left) TLC analysis of fractions. The reactions were performed at 30 °C for 3 h in solutions (10 µl) containing 0.5% β-1,2-glucan (DP121), 50 mM HEPES (pH 8.0), and 1 µl of each fraction. Each reaction solution (1 µl) and a marker were spotted on the TLC plate. Lane M, 0.5% β-1,2-glucan (DP121) as a marker. (top right) Chromatography chart. The blue, red, and magenta lines represent the UV absorbance, conductivity, and percentage of elution buffer, respectively, according to ÄKTA™ prime (Cytiva).


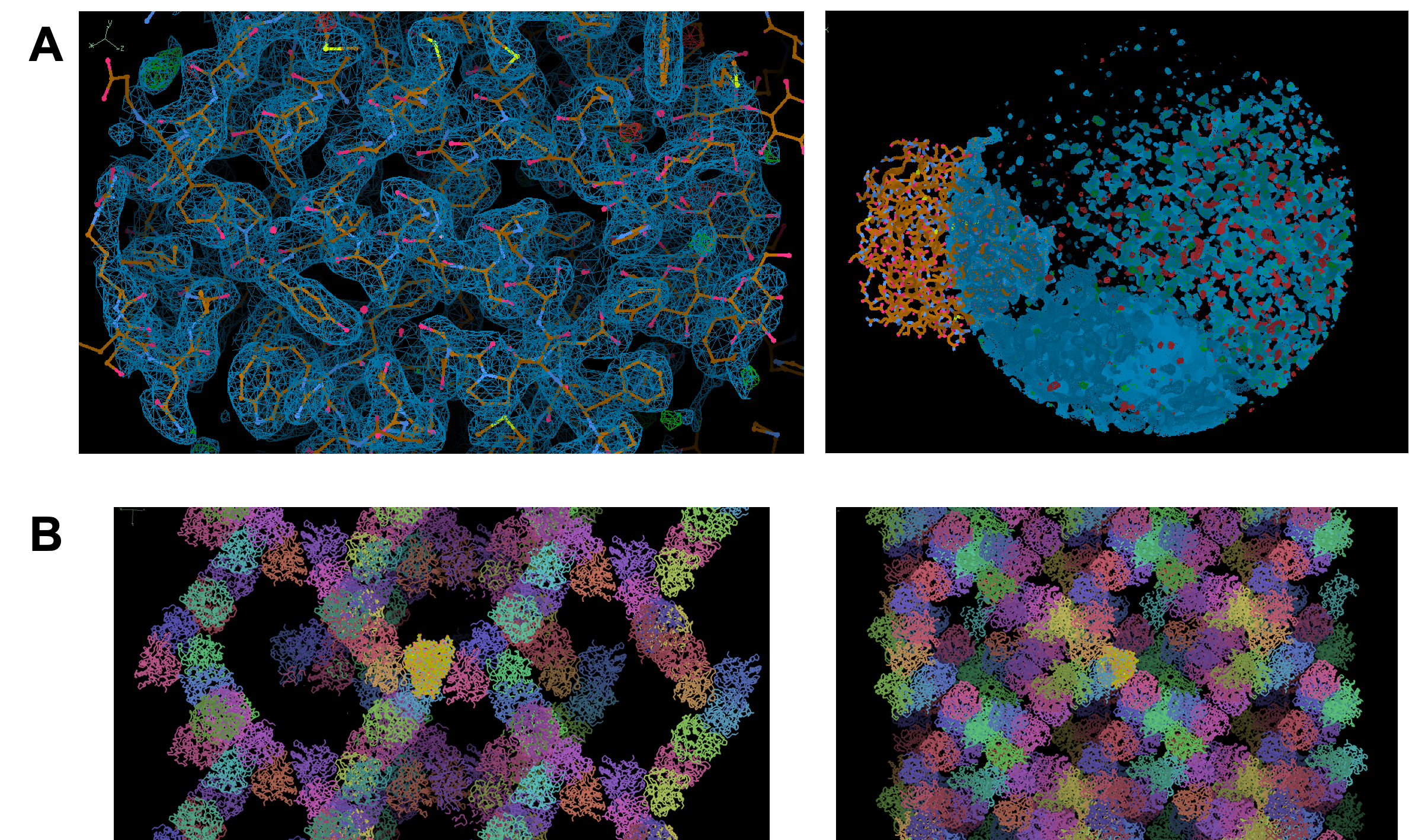


**Figure S18. Crystal structure of XcSGL-Sop_7_ complex**

Figures are prepared by coot (A) and PyMOL (B). (A) Electron density of XcSGL-Sop_7_ complex. (right) Unmodeled electron density is shown in green. (B) Alignment of XcSGL molecules in a crystal. XcSGL molecules are depicted with various colors for visuality.

**References**

Ashkenazy, H., Abadi, S., Martz, E., Chay, O., Mayrose, I., Pupko, T., & Ben-Tal, N. (2016). ConSurf 2016: an improved methodology to estimate and visualize evolutionary conservation in macromolecules. *Nucleic Acids Res*, 44(W1). https://doi.org/10.1093/NAR/GKW408

Bains, R. K., Nasseri, S. A., Liu, F., Wardman, J. F., Rahfeld, P., & Withers, S. G. (2023). Characterization of a new family of 6-sulfo-*N*-acetylglucosaminidases. *J Biol Chem*, 299(10), 105214. https://doi.org/10.1016/j.jbc.2023.105214

Barbeyron, T., Brillet-Guéguen, L., Carré, W., Carrière, C., Caron, C., Czjzek, M., Hoebeke, M., & Michel, G. (2016). Matching the Diversity of Sulfated Biomolecules: Creation of a Classification Database for Sulfatases Reflecting Their Substrate Specificity. *PLOS ONE*, 11(10), e0164846. https://doi.org/10.1371/journal.pone.0164846

Bond, C. S., Clements, P. R., Ashby, S. J., Collyer, C. A., Harrop, S. J., Hopwood, J. J., & Guss, J. M. (1997). Structure of a human lysosomal sulfatase. *Structure*, 5(2), 277–289. https://doi.org/10.1016/s0969-2126(97)00185-8

Drula, E., Garron, M. L., Dogan, S., Lombard, V., Henrissat, B., & Terrapon, N. (2022). The carbohydrate-active enzyme database: Functions and literature. *Nucleic Acids Res*, 50(D1), D571–D577. https://doi.org/10.1093/nar/gkab1045

Ezeji, J. C., Sarikonda, D. K., Hopperton, A., Erkkila, H. L., Cohen, D. E., Martinez, S. P., Cominelli, F., Kuwahara, T., Dichosa, A. E. K., Good, C. E., Jacobs, M. R., Khoretonenko, M., Veloo, A., & Rodriguez-Palacios, A. (2021). *Parabacteroides distasonis*: intriguing aerotolerant gut anaerobe with emerging antimicrobial resistance and pathogenic and probiotic roles in human health. *Gut Microbes*, 13(1), 1922241. https://doi.org/10.1080/19490976.2021.1922241

Flipphi, M. J., Visser, J., van der Veen, P., & de Graaff, L. H. (1993). Cloning of the *Aspergillus niger* gene encoding α-l-arabinofuranosidase A. *Appl Microbiol Biotechnol*, 39(3), 335–340. https://doi.org/10.1007/BF00192088

Hidaka, M., Honda, Y., Kitaoka, M., Nirasawa, S., Hayashi, K., Wakagi, T., Shoun, H., & Fushinobu, S. (2004). Chitobiose phosphorylase from *Vibrio proteolyticus*, a member of glycosyl transferase family 36, has a clan GH-L-like (α/α)_6_ barrel fold. *Structure*, 12(6), 937–947. https://doi.org/10.1016/j.str.2004.03.027

Holm, L. (2022). Dali server: structural unification of protein families. *Nucleic Acids Res*, 50(W1), W210–W215. https://doi.org/10.1093/nar/gkac387

Ishiguro, R., Tanaka, N., Abe, K., Nakajima, M., Maeda, T., Miyanaga, A., Takahashi, Y., Sugimoto, N., Nakai, H., & Taguchi, H. (2017). Function and structure relationships of a β-1,2-glucooligosaccharide-degrading β-glucosidase. *FEBS Lett*, 591(23), 3926–3936. https://doi.org/10.1002/1873-3468.12911

Jumper, J., Evans, R., Pritzel, A., Green, T., Figurnov, M., Ronneberger, O., Tunyasuvunakool, K., Bates, R., Žídek, A., Potapenko, A., Bridgland, A., Meyer, C., Kohl, S. A. A., Ballard, A. J., Cowie, A., Romera-Paredes, B., Nikolov, S., Jain, R., Adler, J., … Hassabis, D. (2021). Highly accurate protein structure prediction with AlphaFold. *Nature*, 596(7873), 583–589. https://doi.org/10.1038/s41586-021-03819-2

Kaur, A., Pickles, I. B., Sharma, M., Madeido Soler, N., Scott, N. E., Pidot, S. J., Goddard-Borger, E. D., Davies, G. J., & Williams, S. J. (2023). Widespread family of NAD^+^-dependent sulfoquinovosidases at the gateway to sulfoquinovose catabolism. *J Am Chem Soc*, 145(51), 28216–28223. https://doi.org/10.1021/jacs.3c11126

Kim, Y. O., Kim, K. K., Park, S., Kang, S. J., Lee, J. H., Lee, S. J., Oh, T. K., & Yoon, J. H. (2010). *Photobacterium gaetbulicola* sp. nov., a lipolytic bacterium isolated from a tidal flat sediment. *Int J Syst Evol Microbiol*, 60(Pt 11), 2587–2591. https://doi.org/10.1099/ijs.0.016923-0

Kurahashi, M., & Yokota, A. (2007). *Endozoicomonas elysicola* gen. nov., sp. nov., a gamma-proteobacterium isolated from the sea slug *Elysia ornata*. *Syst Appl Microbiol*, 30(3), 202–206. https://doi.org/10.1016/j.syapm.2006.07.003

Landau, M., Mayrose, I., Rosenberg, Y., Glaser, F., Martz, E., Pupko, T., & Ben-Tal, N. (2005). ConSurf 2005: The projection of evolutionary conservation scores of residues on protein structures. *Nucleic Acids Res*, 33(SUPPL. 2). https://doi.org/10.1093/nar/gki370

Levasseur, A., Drula, E., Lombard, V., Coutinho, P. M., & Henrissat, B. (2013). Expansion of the enzymatic repertoire of the CAZy database to integrate auxiliary redox enzymes. *Biotechnol Biofuels*, 6(1), 1. https://doi.org/10.1186/1754-6834-6-41

Liu, J., Wei, Y., Ma, K., An, J., Liu, X., Liu, Y., Ang, E. L., Zhao, H., & Zhang, Y. (2021). Mechanistically diverse pathways for sulfoquinovose degradation in bacteria. *ACS Catal*, 11(24), 14740–14750. https://doi.org/10.1021/acscatal.1c04321

Maurício da Fonseca, M. J., Armstrong, Z., Withers, S. G., & Briers, Y. (2020). High-Throughput Generation of Product Profiles for Arabinoxylan-Active Enzymes from Metagenomes. *Appl Environ Microbiol*, 86(23). https://doi.org/10.1128/AEM.01505-20

Mewis, K., Lenfant, N., Lombard, V., & Henrissat, B. (2016). Dividing the Large Glycoside Hydrolase Family 43 into Subfamilies: a Motivation for Detailed Enzyme Characterization. *Appl Environ Microbiol*, 82(6), 1686–1692. https://doi.org/10.1128/AEM.03453-15

Nakajima, M., Tanaka, N., Furukawa, N., Nihira, T., Kodutsumi, Y., Takahashi, Y., Sugimoto, N., Miyanaga, A., Fushinobu, S., Taguchi, H., & Nakai, H. (2017). Mechanistic insight into the substrate specificity of 1,2-β-oligoglucan phosphorylase from *Lachnoclostridium phytofermentans*. *Sci Rep*, 7, 42671. https://doi.org/10.1038/srep42671

Nakajima, M., Toyoizumi, H., Abe, K., Nakai, H., Taguchi, H., & Kitaoka, M. (2014). 1,2-β-oligoglucan phosphorylase from *Listeria innocua*. *PLOS ONE*, 9(3), e92353. https://doi.org/10.1371/journal.pone.0092353

Nakajima, M., Yoshida, R., Miyanaga, A., Abe, K., Takahashi, Y., Sugimoto, N., Toyoizumi, H., Nakai, H., Kitaoka, M., & Taguchi, H. (2016). Functional and structural analysis of a β-glucosidase involved in β-1,2-glucan metabolism in *Listeria innocua*. *PLOS ONE*, 11(2), e0148870. https://doi.org/10.1371/journal.pone.0148870

Neave, M. J., Michell, C. T., Apprill, A., & Voolstra, C. R. (2017). *Endozoicomonas* genomes reveal functional adaptation and plasticity in bacterial strains symbiotically associated with diverse marine hosts. *Sci Rep*, 7, 40579. https://doi.org/10.1038/srep40579

Qian, W., Jia, Y., Ren, S. X., He, Y. Q., Feng, J. X., Lu, L. F., Sun, Q., Ying, G., Tang, D. J., Tang, H., Wu, W., Hao, P., Wang, L., Jiang, B. L., Zeng, S., Gu, W. Y., Lu, G., Rong, L., Tian, Y., … He, C. (2005). Comparative and functional genomic analyses of the pathogenicity of phytopathogen *Xanthomonas campestris* pv. *campestris*. *Genome Res*, 15(6), 757–767. https://doi.org/10.1101/gr.3378705

Shallom, D., Leon, M., Bravman, T., Ben-David, A., Zaide, G., Belakhov, V., Shoham, G., Schomburg, D., Baasov, T., & Shoham, Y. (2005). Biochemical characterization and identification of the catalytic residues of a family 43 β-d-xylosidase from *Geobacillus stearothermophilus* T-6. *Biochemistry*, 44(1), 387–397. https://doi.org/10.1021/bi048059w

Shimizu, H., Nakajima, M., Miyanaga, A., Takahashi, Y., Tanaka, N., Kobayashi, K., Sugimoto, N., Nakai, H., & Taguchi, H. (2018). Characterization and structural analysis of a novel *exo*-type enzyme acting on β-1,2-glucooligosaccharides from *Parabacteroides distasonis*. *Biochemistry*, 57(26), 3849–3860. https://doi.org/10.1021/acs.biochem.8b00385

Silchenko, A. S., Rasin, A. B., Zueva, A. O., Kusaykin, M. I., Zvyagintseva, T. N., Kalinovsky, A. I., Kurilenko, V. V, & Ermakova, S. P. (2018). Fucoidan Sulfatases from Marine Bacterium *Wenyingzhuangia fucanilytica* CZ1127T. *Biomolecules*, 8(4). https://doi.org/10.3390/biom8040098

Stam, M., Lelièvre, P., Hoebeke, M., Corre, E., Barbeyron, T., & Michel, G. (2023). SulfAtlas, the sulfatase database: state of the art and new developments. *Nucleic Acids Res*, 51(D1), D647–D653. https://doi.org/10.1093/nar/gkac977

Van Hoorebeke, A., Stout, J., Kyndt, J., De Groeve, M., Dix, I., Desmet, T., Soetaert, W., Van Beeumen, J., & Savvides, S. N. (2010). Crystallization and X-ray diffraction studies of cellobiose phosphorylase from *Cellulomonas uda*. *Acta Crystallogr F: Struc Biol Cryst Commun*, 66(3), 346–351. https://doi.org/10.1107/S1744309110002642

Vieira, P. S., Bonfim, I. M., Araujo, E. A., Melo, R. R., Lima, A. R., Fessel, M. R., Paixão, D. A. A., Persinoti, G. F., Rocco, S. A., Lima, T. B., Pirolla, R. A. S., Morais, M. A. B., Correa, J. B. L., Zanphorlin, L. M., Diogo, J. A., Lima, E. A., Grandis, A., Buckeridge, M. S., Gozzo, F. C., … Reid, J. S. (2021). Xyloglucan processing machinery in *Xanthomonas* pathogens and its role in the transcriptional activation of virulence factors. *Nat Commun*, 12(1), 1–15. https://doi.org/10.1016/s0021-9258(18)68930-6

Yoshida, E., Hidaka, M., Fushinobu, S., Koyanagi, T., Minami, H., Tamaki, H., Kitaoka, M., Katayama, T., & Kumagai, H. (2010). Role of a PA14 domain in determining substrate specificity of a glycoside hydrolase family 3 β-glucosidase from *Kluyveromyces marxianus*. *Biochem J*, 431(1), 39–49. https://doi.org/10.1042/BJ20100351

Zhang, Z., Dong, M., Zallot, R., Blackburn, G. M., Wang, N., Wang, C., Chen, L., Baumann, P., Wu, Z., Wang, Z., Fan, H., Roth, C., Jin, Y., & He, Y. (2023). Mechanistic and structural insights into the specificity and biological functions of bacterial sulfoglycosidases. *ACS Catal*, 13(1), 824–836. https://doi.org/10.1021/acscatal.2c05405
